# Estimating the similarity of alternative Affymetrix probe sets using transcriptional networks

**DOI:** 10.1186/1756-0500-6-107

**Published:** 2013-03-21

**Authors:** Michel Bellis

**Affiliations:** 1 CNRS, CRBM, UMR-5237, 1919 Route de Mende, Montpellier 34293, France; 2 UMSF, UMR-5237, Montpellier, France

**Keywords:** Bioinformatics, Microarrays, Transcriptional networks, 3’ Alternative poly-adenylation, Alternative probe sets, Python, Matlab

## Abstract

**Background:**

The usefulness of the data from Affymetrix microarray analysis depends largely on the reliability of the files describing the correspondence between probe sets, genes and transcripts. Particularly, when a gene is targeted by several probe sets, these files should give information about the similarity of each alternative probe set pair. Transcriptional networks integrate the multiple correlations that exist between all probe sets and supply much more information than a simple correlation coefficient calculated for two series of signals. In this study, we used the PSAWN (Probe Set Assignment With Networks) programme we developed to investigate whether similarity of alternative probe sets resulted in some specific properties.

**Findings:**

PSAWNpy delivered a full textual description of each probe set and information on the number and properties of secondary targets. PSAWNml calculated the similarity of each alternative probe set pair and allowed finding relationships between similarity and localisation of probes in common transcripts or exons. Similar alternative probe sets had very low negative correlation, high positive correlation and similar neighbourhood overlap. Using these properties, we devised a test that allowed grouping similar probe sets in a given network. By considering several networks, additional information concerning the similarity reproducibility was obtained, which allowed defining the actual similarity of alternative probe set pairs. In particular, we calculated the common localisation of probes in exons and in known transcripts and we showed that similarity was correctly correlated with them. The information collected on all pairs of alternative probe sets in the most popular 3’ IVT Affymetrix chips is available in tabular form at http://bns.crbm.cnrs.fr/download.html.

**Conclusions:**

These processed data can be used to obtain a finer interpretation when comparing microarray data between biological conditions. They are particularly well adapted for searching 3’ alternative poly-adenylation events and can be also useful for studying the structure of transcriptional networks. The PSAWNpy, (in Python) and PSAWNml (in Matlab) programmes are freely available and can be downloaded at http://code.google.com/p/arraymatic. Tutorials and reference manuals are available at BMC Research Notes online (Additional file 1) or from http://bns.crbm.cnrs.fr/softwares.html.

## Findings

### Introduction

Since its introduction in the 90’s, laboratories and companies involved in the development of the microarray technology have produced many different types of microarray platforms. Affymetrix is the most frequently referenced platform and has designed several chip models to quantify the expression level of transcripts by probing their 3’ end (3’-IVT format). These chips show a complicated relationship between probes and genes. Specifically, probes are redundant because in every chip layout a gene is always targeted by a group of 11 or 16 different probes that form a probe set. Probe sets also are redundant as a significant portion of probed genes are targeted by several probe sets. This is the consequence of using full length transcripts and/or expressed sequence tags (EST) to design probe sets.

Soon after the first chips were released, many researchers noticed some inconsistencies in the probe set definitions (i.e., the set of probes considered as targeting the same gene(s) or transcript(s)) and descriptions (i.e., the information indicating which gene(s) or transcript(s) are targeted) and asked for improvements [1,2]. Researchers engaged in this effort mainly used genomic and/or transcript sequences to redefine the probe sets in order to optimize the quality of results by ensuring that the newly defined probe sets targeted only one gene or even one transcript, if alternative transcripts exist [3]. The main drawback of this approach is that the cdf files that combine the probes to define each probe set and that are used by the algorithms which calculate the signal from raw data must be redefined as well. As several groups have developed their own cdf files in addition to the official ones delivered by Affymetrix, users can encounter difficulties in selecting the best fitting cdf file for their needs. Another problem is the difficulty of using the results delivered by modified cdf files. For example, some specialized software that uses the Affymetrix probe set names in the entry might not recognize proprietary probe set names and thus not allow comparing results obtained with different cdf files. Finally, for those interested in massive analysis that relies on files stored in repositories, such as Gene Expression Omnibus (GEO, http://www.ncbi.nlm.nih.gov/geo/) or ExpressArray (http://www.ebi.ac.uk/arrayexpress/), it would be impossible to use results that do not come with the raw files to recalculate signals with modified cdf files. For all these reasons, it seems more sensible to maintain the original definition of probe sets as designed by Affymetrix and to focus on improving their description, as already done in several published studies [4,5].

From this perspective, it is of paramount importance to determine whether alternative probe sets (i.e., sets that map to different regions of the same gene) target the same transcript(s) [6]. Currently, very few methods are available to answer this question, apart from the proposal of using sequence information, which, however, does not allow reaching a conclusion in all circumstances. Indeed, in the absence of exhaustive knowledge on all splice variants, it is not possible with this method to exclude completely that two probe sets located in different exons of the same gene do not target different transcripts. To circumvent this difficulty, it has been proposed to consider two alternative probe sets as targeting the same transcript(s) only when their signals measured in a large number of different biological conditions are highly correlated [7]. We have retained this idea, but propose to develop it in another direction. Indeed, this method can only deliver a binary yes/no answer because it considers pairs of alternative probe sets as either similar or dissimilar according to their Pearson’s correlation coefficient value relative to a predetermined threshold and therefore does not reflects the complex reality of transcriptional regulation. For instance, two genes can be positively correlated in a subset of biological conditions and negatively correlated in another [8,9], and this phenomenon has also been observed using alternative probe sets [6,10-13]. The necessity of preserving the dual correlation that may exist for most pairs of genes prompted us to develop a new method based on another paradigm that instead of considering comparisons between signals (i.e., a co-expression approach that can only detect positive or negative correlations), relies on the signal variation in a large series of comparisons between different biological conditions (i.e., a co-variation approach that can detect both positive and negative correlations between probe sets) [14]. Specifically, this method calculates positive and negative correlation between pairs of probe sets by using two strings that describe the direction of the significant variations observed for each probe set in a large series of comparisons. For instance, the string INNDIDi…, which indicates that a given probe set i is increased in the first and fifth comparison, decreased in the fourth and sixth and unchanged in the second and third comparison, can be used with the corresponding string for probe set j (e.g., IINDDDj…) to calculate positive and negative correlation coefficients between the probe sets i and j. This method applied to all the possible probe set pairs results in two covariation matrices (one for positive and one for negative correlation coefficients) that can be seen as a transcriptional network. We have showed using random pairs of probe sets that the Pearson’s correlation is less efficient than our method to select positively and negatively correlated pairs of probe sets [14]. We propose to use these covariation matrices to ascertain whether alternative probe sets are similar (i.e., they respond identically because they probably target the same transcript(s)) in most of the different biological conditions used to construct the network. Additionally, by using in the same chip model several networks based on different sets of biological conditions, the occurrence of a similarity in different networks can be estimated.

We called this method PSAWN (for Probe Set Assignment With Networks) and its development resulted in a program composed of two parts. One developed in Python (PSAWNpy) recovers and organizes all the probe set information by interrogation of the Ensembl (http://www.ensembl.org/index.xhtml) and AceView (http://www.ncbi.nlm.nih.gov/IEB/Research/Acembly/[15]) databases. The other was developed in Matlab (PSAWNml) to find complex relationships between probe sets and genes and allows estimating the level of similarity of each existing pair of alternative probe sets. Both programs’ output results in tab-separated text files. We applied these programs to all the available human, mouse and rat 3’-IVT Affymetrix chips where it was possible to construct more than 10 networks and thus created a new resource that allows users to download description files generated by these programs and that correspond to the most popular Affymetrix chips (http://bns.crbm.cnrs.fr/download.html).

### Methods

#### Construction of networks

Networks were constructed as indicated [14] and are based on series of experiments (GSE) downloaded from GEO in September 2007. In order to take into account sampling effects that could bias the network structure, we decided to randomly partition all couples of GEO samples (GSM) belonging to the same experimental condition (i.e., biological condition) in groups of 30 conditions and to construct several networks by comparing any two groups of biological conditions, thus generating 30×30 = 900 comparisons each time. As a given group can be used in several comparisons, networks are not fully independent. First, the RDAM algorithm [16] was used to classify each gene as increased (I), decreased (D) or not changed (N) in a given comparison. As a result, an ordered string of 900 symbols (e.g.. IDDNNIDID....DNNNNNNID) was attached to each probe set. Then, positive and negative correlations for all pair of probe sets were calculated by comparing the strings of symbols by calculating the percentage of concordant (i.e. II or DD) or discordant (i.e. ID or DI) positions, after elimination of the non-informative positions (e.g., positions where both strings have a N). The same procedure was applied to random pairs of probe sets to calculate the p-values and to eliminate the values that were not statistically significant. Table 1 lists the Affymetrix chips we used in this study, with the different names associated to them.

**Table 1:** Chips used

**Affymetrix name**	**GEO name**	**Our name**
Human Genome U95A	GPL91	m2
Human Genome U133A	GPL96	m3
Murine Genome U74 Version 2	GPL81	m5
Mouse Genome 430 2.0	GPL1261	m8
Mouse Expression 430A	GPL339	m27
Rat Genome U34	GPL85	m6

GEO: Gene Expression Omnibus.

#### Recovering probe and probe set information

PSAWNpy is a Python program which allows importing users’ data registered in tabular format as well as public data stored in the Ensembl (release 62, April 2011) and AceView databases into local Berkeley databases as explained in the corresponding tutorial (Additional file 1: Figure S1-Figure S10). Data processing allows finding correspondences between probes and genes and to generate text tables that list the probe sets which target a given gene with at least one probe. These tables also display other information about the targeting probes, targeted and not targeted known transcripts and probe location (in exons, introns, and upstream or downstream of a gene within a limit of 2 kb) (Additional file 1: Figure S12 and S13). As some probes were not located in known genes, we created a special category called GOP (group of probes), in which the interval between a probe and its direct neighbour is less or equal to 2 kb.

#### Class and gene assignment of probe sets - similarity of alternative probe sets

Data generated by PSAWNpy were processed by PSAWNml, a Matlab program that constructs biclusters (groups containing two types of entities) of probe sets and genes by applying a clustering algorithm and assigns them to different classes, according to the complexity of the relationship between the number of targeted genes and of targeting probe sets. PSAWNml assigns each probe set to a single gene. When several genes are targeted, PSAWNml selects successively genes with the following characteristics until only one gene is left: the highest number of targeting probes, the best target type (probes in exons > probes in introns), the maximal ratio between number of targeting probe sets and number of groups of probe sets targeting the same transcript(s), the minimal number of targeting probe sets, the gene source (Ensembl > AceView > GOP) and finally the first gene in alphabetic order. The main task of PSAWNml is to study, within a series of networks, some characteristics of alternative probe sets in order to ascertain the similarity of alternative probe sets in a given network (Additional file 1: Figures S16 to S53). The percentage of networks in which a particular pair of alternative probe sets is similar is used to classify alternative probe sets in several classes of similarity. For instance, alternative probe sets that are similar in at least 25% of networks, but not in 50% of networks, have their similarity set to 25%. Then, in each of these classes, all similar probe sets are grouped, thereby defining a group of probe sets that is considered to target a group of transcript(s), and enabling biclustering between probe sets and known/unknown transcripts. Finally, PSAWNml outputs all this information in text files, in a form that allows users to select probe sets with a wide choice of parameters (Additional file 1: Figure S54 and S55).

### Results and discussion

#### Definition of probe set classes

Usually, probe sets are classified in probe sets that target only one gene and probe sets that target several genes. In fact, all relationships between probe sets and genes can be coded in an adjacency matrix, where the cell (i,j) is set to one if gene j is targeted by probe set i, or to zero if not. Application of a bi-clustering method to this matrix will generate biclusters containing all the probe sets and genes that share a type of relationship. As a probe set can target a single gene or several genes and, symmetrically, a gene can be targeted by a single or multiple probe sets, we assumed *a priori* that the actual relationships between probe sets and genes amounted to four classes that are abbreviated as follows: SS, SM, MS and MM by indicating successively the number (single (S) or multiple (M)) of probe set(s) and of gene(s) in a particular bicluster.

**Figure 1:**
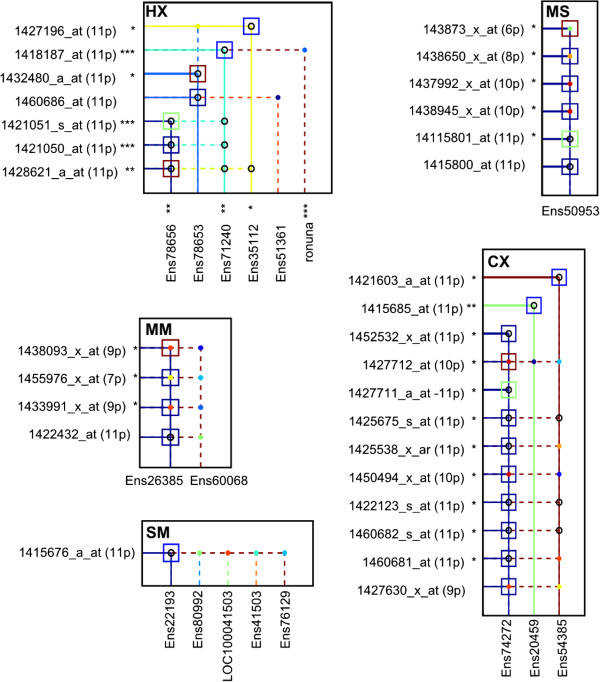
**Classification of biclusters of genes and probe sets (m27-1p).** In each bicluster that exemplifies a particular class, a small circle located at the intersection of the horizontal and vertical lines indicates that the corresponding genes (vertical lines) are targeted by the corresponding probe sets (horizontal lines). In each panel, the probe set name without asterisk is the one used to find genes and probe sets which are related to it. The number of asterisks indicates the depth at which the recursive algorithm found the related gene(s) and probe set(s). A continuous line joins each probe set to the gene it is assigned to by PSAWNml (a thicker horizontal line indicates a probe set which targets a single gene). Interrupted lines join probe sets to all other targeted, but not assigned genes. Empty black and filled coloured round symbols at the intersection between horizontal (probe set) and vertical (gene) lines indicate, respectively, that the gene is targeted by the maximum possible number of probes, or by a smaller number of probes. For genes that are not targeted by the maximum possible number of probes, the change in line colour (from warm towards cool) indicates that the number of targeting probes decreases. If several probe sets are assigned to the same gene, as in class MS, MM, CX and HX, probe sets which are considered as similar by the PSAWN method are boxed by a large square of identical colour. Gene names starting with ‘Ens’ correspond to shortened Ensembl IDs (for instance, Ens78653 corresponds to ENSMUG00000078653); other names are AceView Ids.

However, practical considerations added another layer of complexity. Specifically, as the construction of biclusters was based on the application of a recursive algorithm, we could then distinguish biclusters according to the recursion depth required to reach the stop condition. Accordingly, SS, SM and MS biclusters required only one step (depth equal to one), whereas biclusters that included multiple probe sets and multiple genes could need one step (class MM), two steps (class CX, for complex) or more than two steps (class HX, for hyper-complex). According to the number of recursive steps needed, biclusters had different point densities. They could be saturated (SS not shown, SM, MS and MM classes), or to have more than 50% (class CX) or less than 30% (class HX) of their intersections corresponding to a relationship between a probe set and a gene (Figure 1 and the “O” columns in Table 2). Differently from the MM and CX classes, the HX class could contain biclusters of very large size.

**Table 2:** Biclusters, probe sets and gene frequency

				**NG**	**SSS**	**SM**		**MS**		**MM**			**CX**				**HX**				**R**	**%**
**Sp**	**Ch**	**NN**	**PN**	**P**	**BPG**	**BP**	**G**	**BG**	**P**	**B**	**P**	**G**	**B**	**P**	**G**	**O**	**B**	**P**	**G**	**O**	**P**	**R**
**Hs**	**m2**	21	1	143	4471	1535	4170	1034	2447	134	294	295	869	2586	4273	65	22	1149	6119	21	4834	38
			7	287	5537	1105	2785	1319	3104	130	277	282	692	2188	3452	66	13	127	403	31	4743	38
	**m3**	18	1	503	5117	1010	2719	2132	5346	173	406	382	1127	3770	4324	65	11	6131	19473	25	12506	56
			7	1125	7366	594	1447	3612	9268	161	402	349	970	3420	3135	66	9	108	116	29	12146	55
**Mm**	**m5**	35	1	1278	4680	1306	3692	871	1912	112	235	253	721	1991	3354	66	8	1086	10485	26	3698	30
			7	1736	5680	944	2579	970	2144	75	157	167	551	1662	3077	67	6	165	1138	18	3204	26
	**m8**	15	1	962	8282	1242	3336	5167	14953	264	620	573	2797	11258	8965	63	49	7784	22325	27	31044	69
			7	2004	10043	784	1959	7050	20947	180	425	398	2345	9777	7126	65	37	1121	2937	25	30064	67
	**m27**	21	1	445	5485	1145	3097	2927	7256	257	586	569	1669	5609	6563	65	22	2164	12513	28	13639	60
			7	909	6651	708	1823	3794	9502	171	407	378	1227	4136	4517	67	13	377	1337	25	13202	58
**Rn**	**m6**	15	1	635	2806	709	2546	1049	2523	69	148	147	442	1361	3040	65	10	617	6851	18	4046	46
			7	914	3370	379	1291	1255	3033	60	128	143	283	882	1640	66	5	93	509	17	3888	44

NG: probe sets that do not target any known gene; SS, SM, MS, MM, CX and HX: probe set classes defined in the text; R: alternative probe sets; Sp: species; Ch: our chip model name (see Table 1); NN: number of networks; PN: probe number limit; P: probe set; B: bicluster; G: gene; O: mean density of points in biclusters (%); %R: percentage of alternative probe sets.

It must, however, be emphasized that the classification depends greatly on the probe number limit (i.e. the minimal number of targeting probes required to consider that a probe set detects a gene). For example, the bicluster constructed from 1415856_at in chip m27, with a probe number limit equal to one (m27-1p), belonged to the HX class and had 22350 filled intersections corresponding to 1943 probe sets and 10053 genes. On the other hand, when the probe number limit was increased to seven (m27-7p), the corresponding bicluster was classified as CX and contained only six filled intersections corresponding to four probes and three genes. As shown in Table 2, increasing the probe number limit from one to seven had a drastic influence on the distribution of biclusters, probe sets and genes in the different classes. Indeed, the size of the SS and MS classes increased while it decreased for the other classes. By considering only probe sets that targeted a single gene to eliminate the effect of possible cross-hybridization with other targeted sequences, we observed that the number of similar probe sets was smaller than expected, when one of the two probe sets targeted the common gene with less than seven probes (Additional file 1: Figure S29). In this category of probe sets, the probes that did not target the assigned gene did not *a priori* target anything else in the transcriptome and they could be more subject to random hybridization, which blurs the signal. We therefore considered that a probe number limit set to seven maintained a good balance between specificity (if the probe number limit was too small, the number of genes considered as targets, even if they did not really participate in the signal, was too high) and sensitivity (if the probe number limit was too high, the number of discarded genes, even if they really participated in the signal, was too high).

#### PSAWN method

Column %R of Table 2 shows that up to 69% of probe sets of a chip were alternative (i.e., they mapped to different regions of the same gene). For all analysed chips, with a probe number limit equal to 7, the average was 42%. Such a high proportion of alternative probe sets requires a method to process them in order to know which are similar. We thus devised an original method (PSAWN) based on specific features of these alternative probe set in networks to address this question.

**Figure 2:**
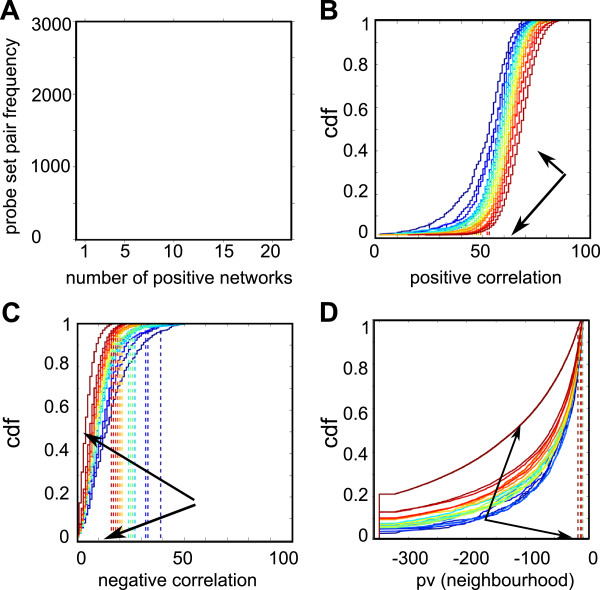
**Distribution of probe set pair properties (m27-1p). A** – Frequency of probe set pairs that belong to the SS class and are positively correlated in 1 to 21 networks. **B** – Distribution of positive correlations between alternative probe sets. **C** – Distribution of negative correlations between alternative probe sets. **D** – Distribution of the neighbourhood overlap p-values of alternative probe sets. In B, C and D, there are 21 curves, one for each subset of alternative probe sets that were positively correlated in 1 to 21 networks (change from cool to warm colours indicates that the number of networks is progressively increasing). The curves corresponding to the alternative probe sets that were positively correlated in all networks and their 5^th^ (B) or 95^th^ (C,D) percentiles (which are used as limits to test whether alternative probe sets must be considered as similar in a given network) are indicated by arrows.

Two probe sets assigned to the same gene may have different positive and negative correlation values in networks constructed from different set of biological conditions. This is also true for the similarity of their neighbourhood, measured by the p-value of occurrence, based on the hypergeometric distribution hypothesis, of an overlap of size equal to or higher than N, given the sizes N1 and N2 of each probe set neighbourhood. We reasoned that *bona fide* similar alternative probe sets should be positively correlated in all networks. A first indication of this was provided by the frequency distribution of alternative probe sets which were positively correlated in a given number of networks (21 in this example). As shown in Figure 2A, the highest frequency corresponded to alternative probe sets that were correlated in all 21 networks. A second indication was that the distributions of positive (Figure 2B) and negative correlations (Figure 2C) and of the p-values of neighbourhood overlap (Figure 2D) for probe set that were correlated in 1 to 20 of the 21 networks formed an ordered group of more or less regularly spaced curves, which were clearly separated from the curve for the probe sets that were correlated in all 21 networks. We therefore chose this special distribution to calculate the 5th percentile of positive correlations (corr^5th^) and the 95th percentile of negative correlations (anti^95th^) and p-value of neighbourhood overlap (overlap^95th^), the limits of which were used to devise a test that classifies a probe set pair j as similar in a network i, if and only if:

$${}_{j}\mathrm{cor}r^{i}⩾\mathrm{cor}r^{5\mathrm{th}}\;{\mathrm{and}\;}_{j}\mathrm{ant}i^{i}⩽\mathrm{ant}i^{95\mathrm{th}}\;\mathrm{and}{\;}_{j}\mathrm{overla}p^{i}⩽\mathrm{overla}p^{95\mathrm{th}}.$$

**Figure 3:**
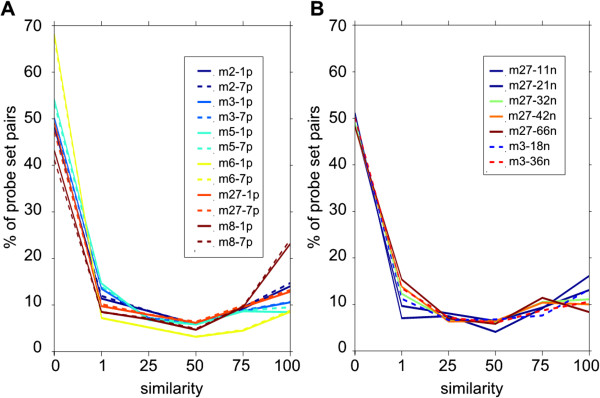
**Similarity distribution. A** – Effect of shifting from one (continuous lines) to seven (interrupted lines) the minimum number of probes that a probe set must have in a gene to be considered as targeting that gene. **B** – Effect of the number of used networks on similarity.

We found that these test limits were specific for each chip model and were largely independent of the number of networks considered, further proving the general validity of our method. Table 3 shows that the stability of the test limits was observed in 11 to 63 networks for chip m27, and in 18 to 36 networks for chip m3. Similarly, changing the biological conditions used to construct the different networks had no effect on these values, as shown for the m27 values which were derived from four different series of 21 networks (Table 3).Alternative probe sets which are similar in one network are not necessarily similar in another one, given the different combination of biological conditions used to construct each network. We therefore listed all pairs of alternative probe sets that were similar in at least a predetermined percentage of networks in five similarity classes: 1% (in fact 1 network, as less than 100 networks have been used to date), 25%, 50% 75% and 100% (Additional file 1: Figure S54 and S55). Then, we defined the similarity of a given pair of alternative probe sets as the highest predetermined number of networks positive for that pair. In other words, a probe sets pair with a similarity of 75% is similar in at least 75% of networks, but not in all of them and a probe sets pair with 0% similarity is not similar in any network. Table 4 and Figure 3 show that most alternative probe set pairs (52% on average) were not similar. Concerning the probe set pairs that were similar, they were distributed more or less equally between the five similarity classes (from 6 to 14% on average as shown in Table 4). Moreover, the frequency of each similarity classes was independent of the tested probe number limit (1 or 7) (Figure 3A), whereas it was slightly affected by the number of considered networks. As expected, with higher numbers of networks, the probability of finding a single network in which a given pair of probe sets was similar or dissimilar was higher, thus increasing the percentage of probe sets in the 1% or 75% similarity classes and decreasing the fraction in the 0% and 100% similarity classes (Figure 3B). Finally, we checked whether the similarity of a specific alternative probe set pair was reproducible when it was calculated using different chip models and/or different number of networks. To do this, we considered the two most frequent similarity classes (0% and 100%) and took into account that a difference of a single positive network is sufficient to shift a particular probe set pair from similarity class 0% to similarity class 1% and from similarity class 100% to similarity class 75%. We therefore counted the percentage of alternative probe sets with a similarity of 0% (or 100%) in one condition that had in another condition an equivalent similarity (0%-1% or 75%-100%). We confirmed that the intra-chip similarity reproducibility was very high (on average 95% for the 0% similarity class and 98% for the 100% similarity class) when the number of networks used varied from 11 to 63, as shown for chip m27 (first line of Table 5 averages 28 comparisons between the eight m27-1p lists of alternative probe sets as indicated in Table 3). We then tested the reproducibility of the similarity classification between chip models (m8 and m27) that had common probe sets (22690 probe sets). On average 90% of alternative probe set pairs in the 0% similarity class and 79% of those in the 100% similarity class in one chip model were also classified in the equivalent class in the other chip model (Table 5). Finally we compared different chip models that had no probe set in common. To do this, we listed all genes that were targeted only by probe set pairs classified in one of the two tested similarity class (0% or 100%) in the control chip (e.g., m2) and also by alternative probe sets in the test chip (e.g., m3). We quantified the similarity reproducibility by calculating the percentage of genes that had all their alternative probe set pairs classified in equivalent similarity classes in the test chip. As shown in Table 5, this procedure applied to genes in the comparison between m8 and m27 gave results that were similar to those obtained when probe sets were compared directly. We found that similarity was still conserved, but changing targeting probe sets had a marked effect because the mean reproducibility for the 0% and 100% similarity classes decreased to 73% and 47%, respectively (m5 vs m8 values of Table 5).

**Table 3:** Limits used to test whether alternative probe set pairs are similar

**Species**	**Chip**	**NetNb**	**ProbeNb**	**CORR**	**ANTI**	**PV**
**Human**	**m2**	21	1	60	10	-10
		21	7	60	10	-10
		18	1	59	8	-10
	**m3**	18	7	60	7	-9
		36	1	61	7	-11
		36	7	61	7	-11
**Mouse**	**m5**	35	1	62	8	-6
		35	7	62	8	-6
	**m8**	15	1	61	16	-32
		15	7	61	16	-33
	**m27**	11	1	61	12	-9
		21	1	62	12	-10
		21	1	62	12	-10
		21	1	62	12	-10
		21	1	62	12	-11
		32	1	63	11	-11
		42	1	64	11	-11
		63	1	65	11	-12
		21	7	62	12	-10
**Rat**	**m6**	15	1	59	8	-17
		15	7	59	8	-17

Chip: our chip model name (see Table 1); NetNb: number of networks used to calculate the limits; ProbeNb: minimal number of probes that a probe set must have in a gene to be considered as targeting that gene; CORR: positive correlation limit; ANTI: negative correlation limit; PV: neighbourhood overlap limit (logarithm of p-value). The m27 limits for ProbeNb = 1 and NetNb = 21 were calculated on networks constructed using different combinations of biological conditions.

**Table 4:** Number of alternative probe set pairs

			**All probe sets**		**Redundant probe sets**				**Similarity**					
**Species**	**Chip**	**NetNb**	**PsNb**	**GeneNb**	**PairNb**	**%Gene**	**%Ps**	**Ps/Gene**	**0%**	**1%**	**25%**	**50%**	**75%**	**100%**
**Human**	**m2**	21	12626	9719	3984	21	38	2,38	48	12	9	6	10	15
	**m3**	36	22283	14132	12385	34	56	2,60	50	15	8	7	10	12
**Mouse**	**m5**	35	12488	9198	2642	18	30	2,23	54	15	7	6	9	9
	**m8**	15	45101	23419	44052	44	69	3,03	68	8	6	3	5	9
	**m27**	21	22690	13996	13133	38	60	2,56	49	11	9	7	10	14
**Rat**	**m6**	15	8799	5759	3596	29	46	2,45	43	9	7	5	10	25
					**Mean**	31	50	2,5	52	12	8	6	9	14

Chip: our chip model name (see Table 1); NetNb: number of networks used; PsNb: total number of probe sets; GeneNb: total number of assigned genes; PairNb: number of alternative probe set pairs; %Gene: percentage of genes targeted by these pairs; %Ps: percentage of alternative probe sets; Ps/Gene: mean number of alternative probe sets per gene; 0%, 1%, 25%, 50%, 75%, 100%: percentage of alternative probe set pairs in the different classes of similarity. These results were obtained with a probe number limit set to 1 and they did not change significantly when the probe number limit was set to 7.

**Table 5:** Reproducibility of similarity

					**Sim 0%**		**Sim 100%**	
**Common**	**Species**	**Comparison**	**NetNb1**	**NetNb2**	**#Tested**	**Reprod**	**#Tested**	**Reprod**
**Probe set pairs**	**Mouse**	m27 vs m27	11 to 63	11 to 63	6311	98	1588	95
		m8 vs m27	15	11	4705	89	1560	75
				21	4705	89	1231	77
				32	4702	90	1042	80
				42	4708	91	946	81
				63	4708	91	803	83
				11to63	4706	89	1159	79
**Genes**	**Human**	m2 vs m3	21	36	409	89	88	69
	**Mouse**	m5 vs m8	35	15	410	73	85	47
		m5 vs m27	35	21	357	77	89	42
		m8 vs m27	15	21	1547	87	493	75

Common: category used to calculate reproducibility, Comparison: chips that are compared, NetNb1, NetNb2: number of networks used respectively in the first chip and in the second chip, #Tested: number of alternative probe sets pairs or genes tested, Reprod: reproducibility of similarity (%), Sim0%: 0% similarity class, Sim100%: 100% similarity class.

As we wanted to group in each bicluster all alternative probe sets that were similar, and not simply to consider each pair of alternative probe sets independently, we developed the following method. First, we searched all probe set triangles built by three pairs of similar probe sets (for instance, the similar (A,B), (B,C) and (C,D) pairs could generate a triangle (ABC)), then we aggregated all triangles with a common edge. In this process, a pair of dissimilar probe sets was sometimes added. For instance, the triangles (ABC) and (ABD) can be aggregated to form the probe set group (ABCD); however, if the triangle (ACD) or (BCD) does not exist, a probe set pair (CD), which is not similar, is *de facto* introduced in the group. We considered this exception as acceptable because the distribution of these added pairs showed that most of them were similar in a high number of networks (see statistics on “bad links” in Additional file 1: Figure S43). However, two triangles could also have only one probe set in common. In this case this probe set was considered as a pivot and was kept apart in a special list that indicated its relationships with the other pairs (Additional file 1: Figure S54).

#### Relationship between similarity and localisation of probes in common transcripts or exons

**Figure 4:**
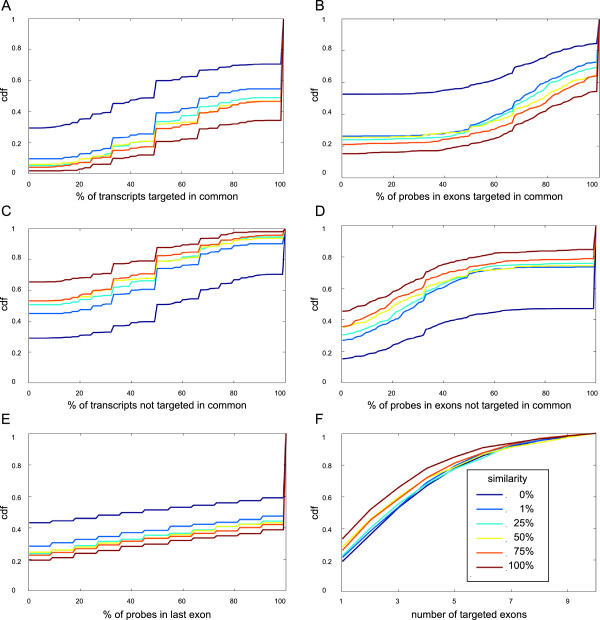
**Relationships between similarity and localisation of probes in common exons or transcripts.** Pairs of alternative probe sets which target a single gene with at least eleven probes in m27-1p were partitioned according to their similarity to assess: **A** - The percentage of transcripts targeted by both probe sets. **B** - The percentage of probes located in exons that are targeted by both probe sets. **C** - The percentage of transcripts targeted by only one of the two probe sets. **D** - The percentage of probes located in exons that are targeted by only one of the two probe sets. **E** - The percentage of probes in one probe set that are located in the last exon. **F** - The number of exons targeted by both probe sets.

In our method, the assessment of similarity of a pair of alternative probe sets depends exclusively on the values of three parameters (positive and negative correlations and neighbourhood overlap) measured in several transcriptomic networks and does not take into account additional information. However, a relationship between similarity and any information on the localisation of probes in transcripts or in exons might exist. Indeed, we observed in all the statistics we studied that the distribution curves were ordered according to the similarity (Figure 4 and also Figure S56 in Additional file 1). For example, 65% of alternative probe set pairs with 100% similarity targeted exactly the same known transcripts, whereas this was true for only 30% for the pairs with 0% similarity (Figure 4A). Similarly, more than 50% of alternative probe sets with 0% similarity did not target any common exon, whereas this value decreased to 20% for probe set pairs with 100% similarity (Figure 4D).

We observed that a high fraction of the studied probe set pairs had all their probes located in the last exon (Figure 4E), which is not surprising considering the 3-IVT format of the studied chips. The last exon is generally complex because it may contain coding and untranslated sequences (3’-UTR), which are often of variable size due to the presence of alternative poly-adenylation sites. Several studies have shown that the expression of different mRNA isoforms characterized by a variable 3’-UTR size is context-sensitive, for example, during spermatogenesis [17] or in cancer cell lines [18]. The differential expression of mRNA isoforms with 3’-UTR of different sizes can be detected thanks to the modification of the signal ratio between probes or probe sets that target both long and short isoforms and those targeting exclusively the long isoform [12,19]. It is therefore likely that similarity as calculated with our program reflects the frequency of alternative post-transcriptional regulations in a large series of biological conditions. More precisely, we postulate that alternative probe sets with 100% similarity map to genes that are seldom prone to this type of regulation. Conversely, alternative probe sets located in genes characterized by two mutually exclusive isoforms would have a similarity of 0% and we hypothesize that an intermediate similarity value indicates the simultaneous presence in variable proportions of two isoforms.

#### Comparison with the results by Elbez and co-workers

In order to compare our results with those already published, we constructed two sets of alternative probe sets that could be assimilated to the ‘good’ and ‘bad’ categories defined by Elbez and colleagues [7]. More precisely, we considered as good all the alternative probe sets that had 100% similarity and as bad all the alternative probe sets with 0% similarity. Elbez et al. defined a third category that grouped all non-informative pairs (NI in Table 6). These pairs have at least one probe set which is considered as absent or with a fold change (defined as the ratio between its signal and the mean signal calculated for the whole dataset) higher than 2 in less than 10% of the experimental conditions in all datasets. Using our approach we did not detect this kind of non-informative probe sets and thus we grouped all alternative probe sets with intermediate similarity (greater than 0% and smaller than 100%) in an ‘intermediate’ category (Inter in Table 6). This approach allowed qualifying all the results based on a binary classification by introducing the probabilistic notion of similarity: only 22% of the alternative probe sets defined as good in the Elbez’ classification were always considered as similar, while 50% of them targeted the same transcript(s) only in specific circumstances. Similarly, most of the bad pairs (77%) according to Elbez and colleagues were also classified as having 0% similarity by our approach; however, 22% of them had probe sets that were similar in some circumstances. Finally, our approach, by considering far more biological samples, allowed us to determine the nature of pairs considered as non-informative by Elbez: 67% of these pairs were bad and 31% intermediate.

**Table 6:** Comparison between classifications obtained using the Pearson’s correlation coefficient or the PSAWN method (m3-1p)

			**Elbez (11722 pairs)**		
			**Good**	**Bad**	**NI**
		**10565 in common**	**46**	**12**	**42**
PSAWN (12385 pairs)	Good	11	91 | 22	1 | 1	8 | 2
	Bad	50	25 | 28	19 | 77	56 | 67
	Inter	39	60 | 50	7 | 22	33 | 31

In common: probe set pairs belonging to the categories defined respectively in our study and in the work by Elbez and co-workers. Numbers placed before (or after) the vertical lines sum up to 100 % horizontally (or vertically) and indicate how probe sets belonging to a particular category in one study were distributed in the three categories of the other study.

#### Rank difference distributions

**Figure 5:**
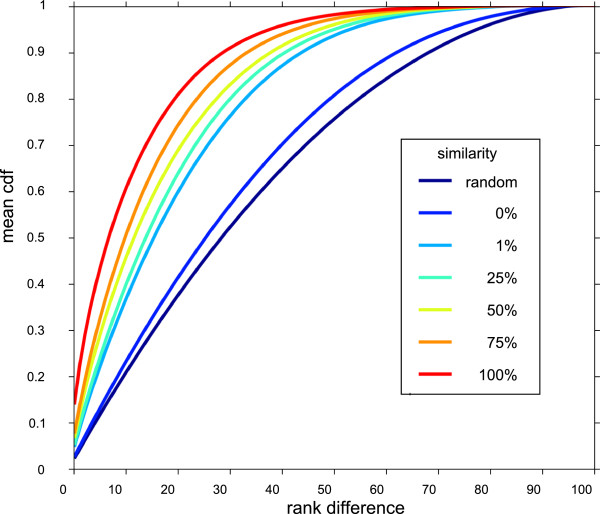
**Distribution of the absolute values of rank difference of alternative probe sets (m27-1p).** All alternative probe sets in m27-1p were partitioned according to their similarity (random indicates a list of randomly paired probe sets). For each pair belonging to a given similarity class the cumulative distribution of the absolute values of rank difference was calculated in all biological conditions and the mean of all cdf was plotted.

The method we have developed delivers information that is probabilistic by nature (and we are aware that the probability that a given pair of probe sets is similar in for instance 50% of networks does not mean that this pair is similar in 50% of biological conditions). However, there should be a link between the similarity value of an alternative probe set pair and its propensity to be similar in a given biological condition. We first normalized each biological condition by replacing each raw signal sorted for a biological condition by its rank (expressed on a 0-100 scale). Then, we used the absolute value of rank difference between alternative probe sets as an indication of their similarity in a given biological condition. We observed that the mean values of all distribution curves calculated for each pair in all biological conditions were ordered according to the similarity value (Figure 5). This observation paves the way for developing new methods to assess the similarity of alternative probe sets in a given experimental condition, a question for which no answer exists to date.

#### Distribution of positively and negatively correlated pairs of alternative probe sets in a large series of comparisons

**Figure 6:**
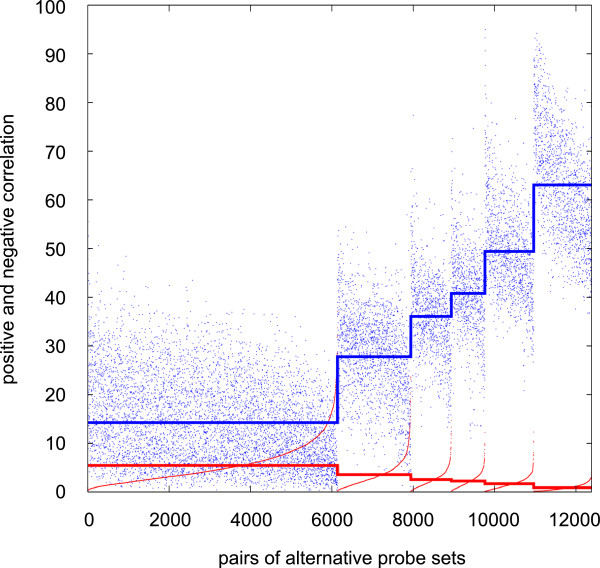
**Distribution of positive and negative correlations for pairs of alternative probe sets in a series of comparisons (m3).** Positive and negative correlations are plotted in blue and red, respectively. Each pair of alternative probe sets is represented by two points aligned vertically. In each level of similarity, all the probe set pairs are ordered relative to their negative correlation. The horizontal lines indicate the mean value of the correlations in each level of similarity.

**Figure 7:**
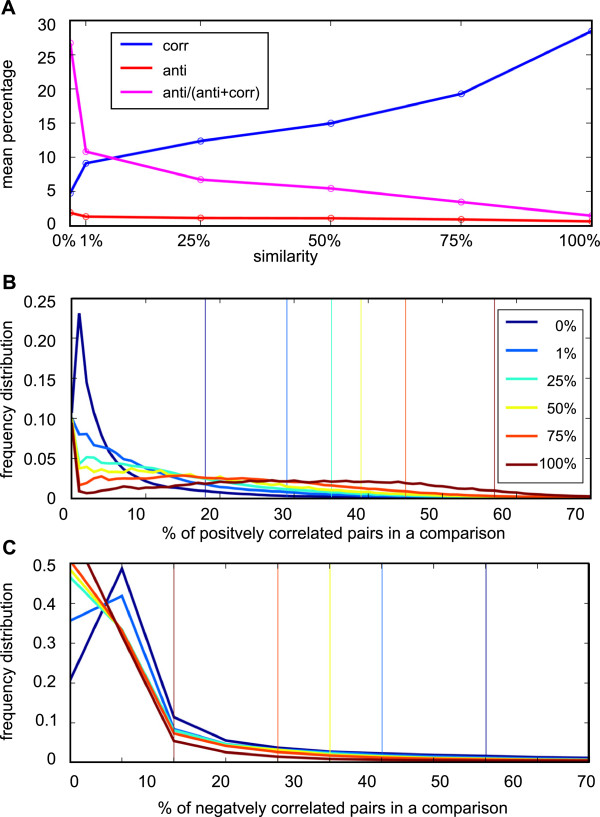
**Percentage of positively and negatively correlated pairs of alternative probe sets in a single comparison (m3). A** – The mean percentage of positively and negatively correlated pairs of probe sets in each similarity class is displayed in blue and red, respectively. The percentage of negatively correlated pairs relative to the sum of positively and negatively correlated pairs is indicated in magenta. **B** - Distribution of the percentage of positively correlated pairs in 279378 comparisons for each class of similarity. **C** - Distribution of the percentage of negatively correlated pairs in 279378 comparisons for each class of similarity. In B and C, the position of the 95^th^ percentile is indicated by a vertical line.

Recently, several algorithms have been developed to find alternative probe sets that are differentially regulated in two biological conditions and some have been tested in specific models, for instance to characterize isoform-specific degradation during oocyte maturation [12], or disturbance of alternative splicing regulation following transfection with oncogenes [13]. This prompted us to study the similarity distribution of positively and negatively correlated pairs of alternative probe sets in a large series of comparisons. For example, for chip m3, in our database there are 748 biological conditions that we used to construct the corresponding networks. More specifically, all possible comparisons (748*747/2 = 279378) were analysed by applying the RDAM algorithm [16] and the networks constructed by selecting appropriate subsets of these comparisons. We then used these already processed data to recover for each of the 12385 pair of alternative probe sets found in m3 the positive and negative correlation values, as explained in the Method section. The different levels of similarity defined in the networks correspond to specific combinations of positive and negative correlation values (Figure 6). These values were calculated for all chip models used in this study and can be useful for estimating whether the positive or negative correlations observed for a particular pair of alternative probe sets in a given comparison between two biological conditions deviate significantly from what observed usually. We also computed for each level of similarity the percentage of alternative probe set pairs that were positively or negatively correlated in each comparison. The mean values (in percentage) of each level of similarity in chip m3 (Figure 7A) indicated that there was an almost linear relationship between the percentage of positively or negatively correlated pairs of alternative probe sets and similarity > 0%. Conversely, alternative probe sets belonging to the similarity class 0% had a much lower percentage of positively correlated probe set pairs (4.5%) and a higher percentage of negatively correlated probe set pairs (1.6%). The mean relative percentage of negatively correlated pairs of probe sets inside a comparison allowed characterizing each level of similarity by a single value, underlining the importance of both positive and negative correlations for defining the similarity classes (magenta curve in Figure 7A). Finally, we considered the real distribution of these percentages in the whole set of comparisons and found that the percentage of positively or negatively correlated pairs of alternative probe sets in a given comparison was highly variable (Figure 7B and 7C). The 95^th^ percentiles of these distributions are indicated in Table 7 and can be used to estimate whether the frequency of positively or negatively correlated pairs of alternative probe sets belonging to a particular similarity class is abnormal in a given comparison.

**Table 7:** **95**^**th **^**percentile of the distribution of the percentage of positively and negatively correlated pairs**

		**Similarity level**				
**Chip**	**Statistics**	0	1	25	50	75
**m2**	**Corr**	30	39	46	51	54
	**Anti**	17	13	10	11	9
	**Anti/(Corr+Anti)**	47	31	25	21	18
**m3**	**Corr**	31	41	47	52	56
	**Anti**	18	13	11	11	9
	**Anti/(Corr+Anti)**	48	30	24	21	17
**m5**	**Corr**	31	40	47	50	55
	**Anti**	21	14	14	13	11
	**Anti/(Corr+Anti)**	46	33	29	25	20
**m6**	**Corr**	26	43	48	52	60
	**Anti**	17	17	14	17	12
	**Anti/(Corr+Anti)**	44	39	31	31	25
**m8**	**Corr**	35	48	55	59	65
	**Anti**	28	24	23	22	19
	**Anti/(Corr+Anti)**	48	37	33	32	28
**m27**	**Corr**	41	54	57	61	67
	**Anti**	30	25	22	20	18
	**Anti/(Corr+Anti)**	47	37	32	29	24

#### Clustering of transcriptional networks

**Figure 8:**
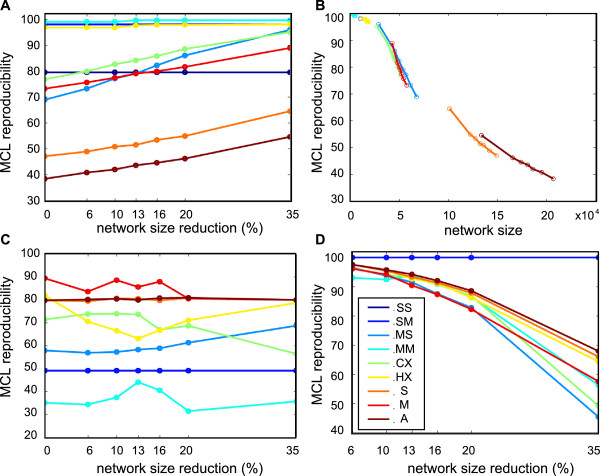
**Effect of probe set classes and similarity on MCL clustering (m8 and m27). A** – Changes in the pruning score relative to the probe set class (classes are colour-coded as indicated in panel D), and to the network size used for the clustering process. The abscissa indicates the network size reduction (in percentage) obtained by merging the alternative probe sets with a similarity ranging from 100% (6%) to 0% (35%). Size reduction 0% corresponds to the full network. **B** – Same data as in A, but the abscissa indicates the real size of the sub-networks used for clustering. For each probe set class, the different subsets corresponding to the different used networks are indicated by small circles (from the full network, on the right, to the network with 35% reduction, on the left). **C** – Inter-chip reproducibility of MCL clustering in m8 and m27. The reproducibility of one cluster corresponds to the number of common probe sets divided by the geometric mean of the cluster sizes in m8 and m27. The reproducibility of clustering is the weighted mean of the ten first clusters (the weights are the inverse of the geometric mean of the cluster sizes). **D** – Intra-chip reproducibility of MCL clustering in m27 in networks that were reduced by probe set merging relative to the full network.

Once a transcriptional network has been constructed, it is of paramount importance to understand its structure. This can be done by permuting the lines and columns of the matrix in which the network is coded in order to bring together genes that work together (i.e., clusterization). The Markov clustering algorithm (MCL) has been extensively used and is very efficient in finding large regions that contain hundreds of genes [20,21]. We have already shown that this method gives reproducible results when applied to networks constructed using different chip models and from different species through the constant delimitation of large regions corresponding to six well defined physiological functions: nuclear processes, development, immunity, nervous system and general and energetic metabolism [16]. Having defined six classes of probe sets (SS to HX) and several levels of similarities for alternative probe sets, we investigated the results of clusterization using MCL when different subsets of probe sets were used. To do this, we started from the full network that contained all probe sets of the m27 and m8 chip models and constructed six networks that were characterized by different levels of probe set merging. The merging process consisted in replacing several probe sets by a single probe set for which the positive and negative correlations with other probe sets were the mean of the respective values of the replaced probe sets. We used the different level of similarity defined for alternative probe sets to implement this process. For example, all the pairs of alternative probe sets that targeted the same group of transcripts and had a similarity of 50% were merged, thus reducing by 13% the number of probe sets. We calculated that the size reduction of networks constructed by using a similarity level of 100%, 75%, 50%, 25%, 1% and 0% was 6%, 10%, 13%, 16%, 20% and 35%, respectively. We then tested the effect of the multiplicity of probe set classes and levels of similarity on three outputs: the efficiency of memory utilisation by MCL, the inter-chip and the intra-chip reproducibility of clustering. MCL simulates random walks within a graph. During the expansion step, the power of the initial matrix using the normal product matrix must be calculated. This may not be possible due to memory limitations when working with large networks. Therefore, to work with a sparse matrix and to limit the quantity of memory, MCL removes the smallest entries of the matrix, a procedure called pruning. At the end of the clustering process, MCL delivers a pruning score that ranges from 0 (worst) to 100 (best) to indicate how much data was sacrificed in order to make clustering possible. When MCL was used with the not merged network, the pruning score was highly variable, ranging from 40, when all probe sets were used, to a perfect score of 100 for the SM, MM and HX classes. However, we observed that the score increased in all classes (except for classes SS and SM where probe sets cannot be merged) concomitantly with the reduction of the network size (and thus higher merging levels) (Figure 8A). The variations of the pruning score can be mainly explained by the different size of the considered classes, from few hundreds (class SS, MM and HX) to more than 20 000 probe sets (all probe sets) (Figure 8B). As the clusters with pruning scores of 80 or less may significantly differ from clusters obtained with a perfectly computed MCL process (without pruning), it is generally suggested to get higher scores by spending more memory resources. This is not always possible. Therefore, to assess the quality of the clusters obtained with pruning scores lower than 100, we compared the clusters obtained in the same conditions but in two different chips (m8 and m27, Figure 8C) and found that the pruning score had not direct effect on the reproducibility of clustering. On the other hand, the size of the subset of probe sets used for MCL clustering induced indirectly an inverse relationship between reproducibility and pruning score. Indeed, as a rule, extracting a subset of any network may degrade the quality of clustering, especially if the subset is small. Moreover, we observed that also the probe set class had some influence. For example, class HX, which is smaller than class MS, had a better reproducibility (Figure 8C). Similarly, class M (all probe sets that target more than one gene) showed the best reproducibility, although its size was similar to that of the classes MM and CX and much smaller than that of class S (all the probe sets that target only one gene) and A (all probe sets). We then compared the results obtained for m27 after different levels of size reduction and the results obtained with the full network. The reproducibility of clustering was roughly independent of the probe set class, but decreased steadily with the reduction of the network size: from 98% with a size reduction of 6% to 92% for a 20% size reduction (Figure 8D). However, the decrease was not linear and became more and more pronounced as the merging process involved alternative probe set with lower similarity. These results shows that i) low pruning scores are not a real concern in this case, although it cannot be excluded that using more memory resources might improve the reproducibility of results obtained with large subsets (classes A, S and M) and ii) MCL clustering is sensitive to the level of similarity used to reduce the size of networks but can tolerate a rather important level of size reduction (<=20%).

### Conclusion

Our work shows that transcriptional networks that integrate both positive and negative correlations for all possible pairs of probe sets are a powerful tool for assessing the similarity of alternative probe sets. We have extended the measure of the similarity between two alternative probe sets beyond its original formulation based on a binary classification to be closer to the biological reality by considering it as a probability. We demonstrate that each class of similarity corresponds to a particular combination of positive and negative correlation levels and we show that the method is robust and that similar results are obtained when using different chip models or different numbers of networks. Moreover, we found that the different levels of similarity we defined were correlated as expected with all the tested independent measures concerning the localisation of probe sets in exons and in known transcripts. For the analysis we set up a new classification of probe sets relative to the number of genes they target, and showed that these classes behave differently in MCL clustering.

Our work has led to the development of a complete set of open source tools in Python and Matlab that allow the complete analysis of the probe set characteristics within transcriptional networks. The software we developed delivers a full textual description of each probe set (which genes, exons and transcripts are targeted by a given number of probes) as well as information on the number and properties of secondary targets (Additional file 1: Figures S12, S13, S14, S15 and Figure S54). The software outputs another file which gives the similarity of each pair of alternative probe sets and the probability that the pair is positively or negatively correlated in a huge series of comparisons between two biological comparisons (observed probabilities). We also added several scores that allow to find relationships between similarity and localisation of probes in common transcripts or exons (Additional file 1: Figure S55), making these data particularly suitable for the study of 3’ alternative transcripts. The study of the transcriptome structure is another field that could benefit from these data as shown with MCL clustering. In all these studies, and particularly if interested in the fine structure of networks, it is important to verify that similar results are obtained with different models of chips. The comparison between the chip models m8 and m27 was straightforward because all the probe sets of m27 are included in m8, but it is an isolate case, and one-to-one correspondence between the probe sets is needed. The study of probe set neighbourhood is certainly a good approach to solve this problem.

Many Affymetrix users carry out simple analyses mostly to detect the more varying genes. Users interested in 3’ alternative poly-adenylation (the main alternative type of transcripts targeted by 3-IVT chips) will find in the tables we generated the information on alternative probe sets that target the genes which are in their top list. For instance, if one alternative probe set is up-regulated and the other down-regulated, the level of similarity and the percentage of positive and negative correlations indicate whether this event is unexpected and deserves more investigation.

Finally these data and these programs could be used to solve related questions. As already mentioned, the fact that the rank difference between two alternative probe sets is correlated with their similarity (Figure 5) is a strong indication that it could be possible to determine whether they target the same group of transcripts in a given biological condition. Gene Set Enrichment Analysis (GSEA) is a promising method that does not test each gene individually, but is interested in determining whether a known gene set (e.g., a Kegg pathway, or a network module) is recruited in a particular biological condition [22]. For this probe set results are converted into gene results and alternative probe sets are replaced by the one with the highest signal. This practice has not been thoroughly tested and our tools and data would allow verifying whether this is true for all level of similarity and also whether better merging schemes could be devised.

## Availability and requirements

•Project name: PSAWN

•Project home page: https://github.com/mbellis/

•Operating system: Platform independent

•Programming language: Python, Matlab

•License: CeCILL

### Availability of supporting data

The data sets supporting the results of this article are available in the CRBM repository in http://bns.crbm.cnrs.fr/download.html)

## Abbreviations

GOP: Group of probes; GSEA: Gene set enrichment analysis; MCL: Markov clustering; PSAWN: Probe set assignment with networks; RDAM: Rank difference analysis of microarrays.

## Competing interests

The author declares that he has no competing interests.

## Supplementary Material

Additional file 1 **PSAWN manual.** Tutorial and reference for PSAWNpy and PSAWNml. Click here for file
