# Supplementary material for: Estimating the similarity of alternative Affymetrix probe sets using transcriptional networks
Source: BMC Res Notes. 2013 Mar 21;6:107. doi: 10.1186/1756-0500-6-107 (PMC3630002; doi:10.1186/1756-0500-6-107)
Supplement: Additional file 1 — PSAWN manual. Tutorial and reference for PSAWNpy and PSAWNml. [file 1756-0500-6-107-S1.pdf]

# PSAWN Documentation

***Release 1.0e***

**Michel Bellis**

December 4, 2012

# CONTENTS


**1 TUTORIALS** **3**
- 1.1 Installation . . . 3
- 1.2 Needed packages . . . 4
- 1.3 External data . . . 4
- 1.4 User data . . . 8
- 1.5 **PSAWNpy** . . . 9
- 1.6 **PSAWNml** . . . 25

**2 REFERENCE MANUALS** **71**
- 2.1 **PSAWNpy** . . . 71
- 2.2 **PSAWNml** . . . 89

**Python Module Index** **105**

**Index** **107**

* PSAWN is the acronym of **Probe Set Assignation With Networks**.

* Microarrays are devices which allow to measure simultaneously the expression
level of thousands of genes in a particular biological condition. Transcription products derived from the genes (transcripts or mRNA) are recognized by a set of probes (a probeset in Affymetrix technology) that match some transcript sequence(s). In some chips, some genes are targeted by several probesets. As the mRNA derived from a particular gene can be different (alternative transcription), the signals delivered by these probesets are not always consistent.

* PSAWN uses the information contained in transcription networks, to group
or probesets that really target the same group of transcript(s).

* PSAWN works with two pieces of software. The first one is PSAWNpy, a Python package that
recovers gene and transcript definitions, and probe localisation from specialized databases (Ensembl, AceView). The second one is PSAWNml, a Matlab application which uses this information to construct different classes of probesets based on combinations between multiplicity of probesets and multiplicity of genes.

These scripts where developped in Python 2.6 and Matlab R12. Package numpy which is used by PSAWNpy is not supported by Python version above 2.6.

CHAPTER

ONE

# TUTORIALS

## 1.1 Installation

Create a main directory to unzip program and data files. For example create:

**psawn** main PSAWN directory

### 1.1.1 PSAWNpy

Unzip PSAWNpy_V1.0c.zip in this directory. Python scripts are located in:

**psawn/prog/psawn_py** Python scripts

Several subdirectories are created to receive data used or created by the programs:

**psawn/data/affydata** Affymetrix probe sequence files (FIG1,2)

**psawn/data/acedata** AceView files (FIG3,4,5)

**psawn/data/ensdata** Ensembl files (FIG6,7)

**psawn/data/pydata** data bases created and used by PSAWNpy

**psawn/data/rawdata** text files describing chip models and listing their probesets (FIG8,9,10)

Modify setenviron.py module in psawn/psawn_py to update corresponding environment variables values.

### 1.1.2 PSAWNml

Unzip PSAWNml_V1.0c.zip in this directory.

Matlab scripts are located in: :psawn/prog/psawn_ml: Matlab scripts Update the Matlab path to add this new program directory.

Several subdirectories are created to receive data used or created by the programs: :psawn/data/net/m8/: download inside this folder, all networks :psawn/data/net/m8/data/experiment: download inside this folder ranked signals :psawn/data/pydata/mouse/txt: data created by PSAWNpy :psawn/data/rawdata: text files describing chip models and listing their probesets (may be already created if

PSAWNpy is installed, data are identical and may be overwritted) (FIG8,10,11)

## 1.2 Needed packages

Install Numpy to allow vector manipulation in PSAWNpy.

Ensembl data stored in remote MySql tables are queried from inside PSAWNpy by using a MySql connection. To do this MySqlDb package must be installed (Windows installers can be found here).

Merge_ps in PSAWNml needs *Cliquer* which is a set of C routines, developed by Patric Östergård, for finding cliques in an arbitrary weighted graph (downloaded from http://users.tkk.fi/pat/cliquer.html).

## 1.3 External data

Some external data must be loaded, in particular if one wants to use AceView informations.

Developped example concerns the Affymetrix Mouse Genome 430 2.0 Array ‘ referenced in GEO as GPL1261 platform and as m8 in our nomenclature. Affymetrix probe definition files must be downloaded from their site as shown in the following figure.

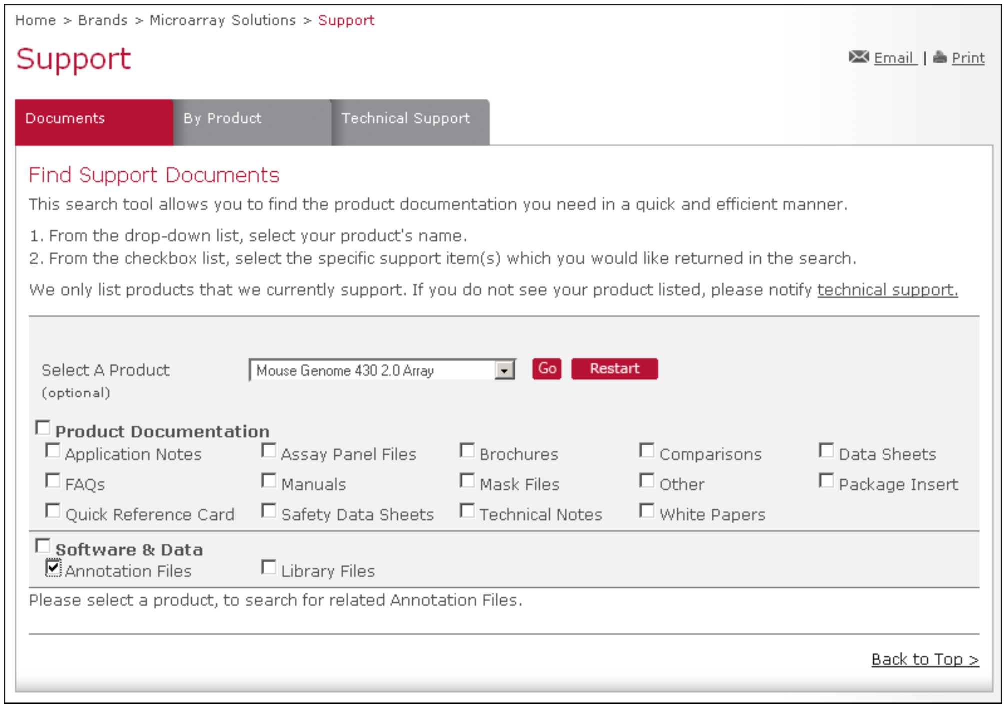


**fig.1** Select the right chip model in the ‘Select A Product’ drop-down list, check the ‘Annotation Files’ item in ‘the Software & Data’ checkbox list, click on Go, and download the file marked ‘Mouse430A_2 Probe Sequences, Tabular (4.0 MB, 8/20/08)’ into data/affydata.

| Probe Set Name | Probe X | Probe Y | Probe Interrogation Position | Probe Sequence | Target Strandedness |
|---|---|---|---|---|---|
| 1415670_at | 269 | 753 | 2436 | GGCTGATCACATCCAAAAAGTCATG | Antisense |
| 1415670_at | 486 | 557 | 2513 | GAGGAAACGTTCACCCTGTCTACTA | Antisense |
| 1415670_at | 780 | 603 | 2521 | GTTCACCCTGTCTACTATCAAGACA | Antisense |
| 1415670_at | 479 | 899 | 2533 | TACTATCAAGACACTCGAAGAGGCT | Antisense |
| 1415670_at | 653 | 375 | 2556 | CTGTGGGCAATATTGTGAAGTTCCT | Antisense |
| 1415670_at | 70 | 475 | 2583 | GAATGCATCCTTGTGAGAGGTCAGA | Antisense |
| 1415670_at | 169 | 549 | 2597 | GAGAGGTCAGACAAAGTGCCAGAAA | Antisense |

**fig.2** Affymetrix Mouse430_2.probe_tab file (the first line belongs to the file).

AceView data must be downloaded. Only genes_gff and mrnas_fasta files are used.

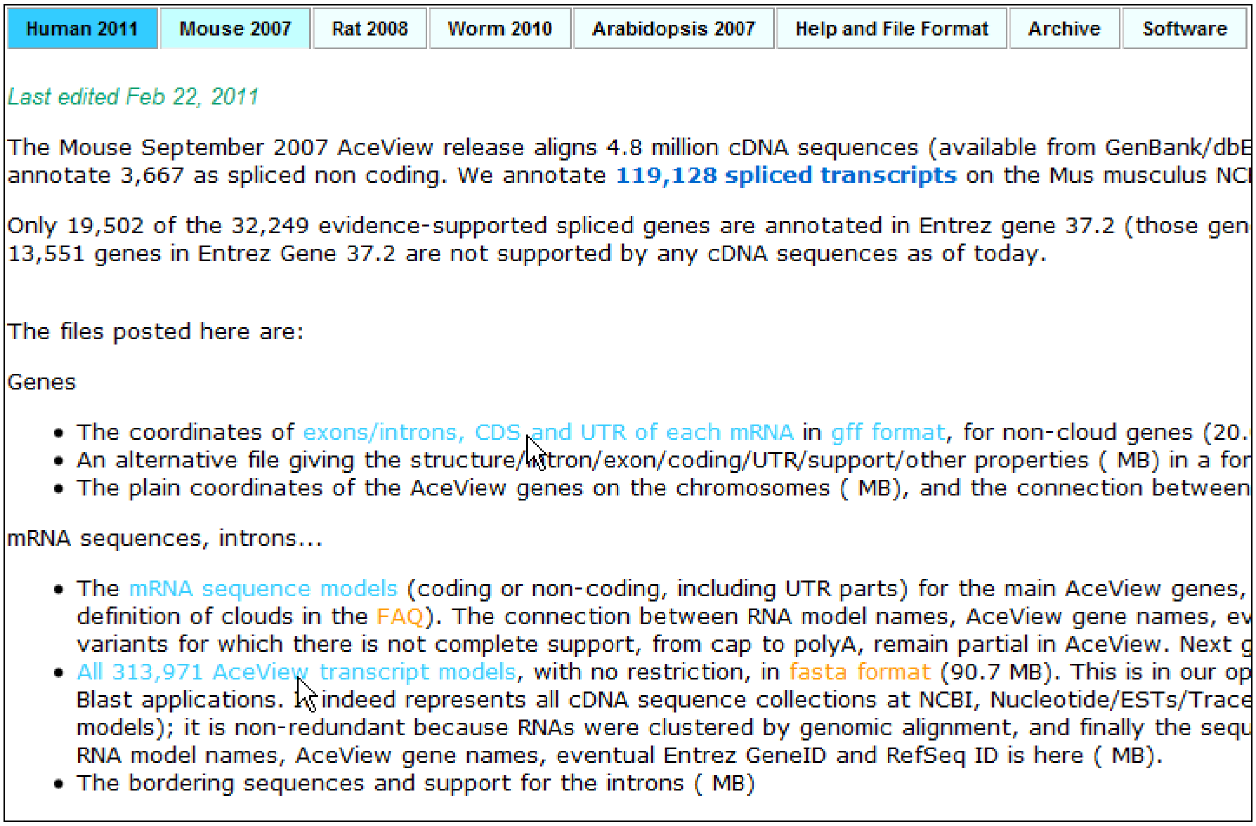
Human 2011 | Mouse 2007 | Rat 2008 | Worm 2010 | Arabidopsis 2007 | Help and File Format | Archive | Software

*Last edited Feb 22, 2011*

The Mouse September 2007 AceView release aligns 4.8 million cDNA sequences (available from GenBank/dbE
annotate 3,667 as spliced non coding. We annotate **119,128 spliced transcripts** on the Mus musculus NC

Only 19,502 of the 32,249 evidence-supported spliced genes are annotated in Entrez gene 37.2 (those gen
13,551 genes in Entrez Gene 37.2 are not supported by any cDNA sequences as of today.

The files posted here are:

Genes

- The coordinates of exons/introns, CDS and UTR of each mRNA in gff format, for non-cloud genes (20.
- An alternative file giving the structure/intron/exon/coding/UTR/support/other properties ( MB) in a for
- The plain coordinates of the AceView genes on the chromosomes ( MB), and the connection between

mRNA sequences, introns...

- The mRNA sequence models (coding or non-coding, including UTR parts) for the main AceView genes, definition of clouds in the FAQ). The connection between RNA model names, AceView gene names, ev variants for which there is not complete support, from cap to polyA, remain partial in AceView. Next g
- All 313,971 AceView transcript models, with no restriction, in fasta format (90.7 MB). This is in our op Blast applications. It indeed represents all cDNA sequence collections at NCBI, Nucleotide/ESTs/Trace models); it is non-redundant because RNAs were clustered by genomic alignment, and finally the sequ RNA model names, AceView gene names, eventual Entrez GeneID and RefSeq ID is here ( MB).
- The bordering sequences and support for the introns ( MB)

**fig.3** Download the two files indicated by an arrow (AceView.mm_37.genes_gff.tar.gz and AceView.mm_37.all_mrnas_fasta.tar.gz) into data/acedata and decompress them.

| chromosome | source | feature | start | end | score | strand | frame | group |
|---|---|---|---|---|---|---|---|---|
| 1 | AceView | exon | 66863948 | 66864160 | . | -0 | -0 | gene_id 1110028C15Rik; Gene_type Main; transcript_id 1110028C15Rik.aSep07; exon_number 1 |
| 1 | AceView | intron | 66854179 | 66863947 | . | -0 | -0 | gene_id 1110028C15Rik; Gene_type Main; transcript_id 1110028C15Rik.aSep07; type gc_ag |
| 1 | AceView | exon | 66854099 | 66854178 | . | -0 | -0 | gene_id 1110028C15Rik; Gene_type Main; transcript_id 1110028C15Rik.aSep07; exon_number 2 |
| 1 | AceView | intron | 66848743 | 66854098 | . | -0 | -0 | gene_id 1110028C15Rik; Gene_type Main; transcript_id 1110028C15Rik.aSep07; type gt_ag |
| 1 | AceView | start_codon | 66848711 | 66848713 | . | -0 | -0 | gene_id 1110028C15Rik; Gene_type Main; transcript_id 1110028C15Rik.aSep07; product_id 1110028C15Rik.aSep07; |
| 1 | AceView | CDS | 66847623 | 66848713 | . | -0 | -0 | gene_id 1110028C15Rik; Gene_type Main; transcript_id 1110028C15Rik.aSep07; product_id 1110028C15Rik.aSep07; exon_number 3 |
| 1 | AceView | exon | 66847623 | 66848742 | . | -0 | -0 | gene_id 1110028C15Rik; Gene_type Main; transcript_id 1110028C15Rik.aSep07; exon_number 3 |
| 1 | AceView | intron | 66824683 | 66847622 | . | -0 | -0 | gene_id 1110028C15Rik; Gene_type Main; transcript_id 1110028C15Rik.aSep07; type gt_ag |

**fig.4** AceView x1.genes_gff.1.gff file in gff format (the first line does not belong to the file).

```
>MRNA:0610010K06Rik.aSep07
tggggaccccaggctacatctctagggcccagacagaagctcaagagcca
tccaaccagagtctcagctttgctttgcagaagtctaatctggaagccct
gcagtgtcagcacctcaggcttctcccacagactgagcacattttgtgaa
gatgacccattggtttcataggaacccattaaaagctacagctcccgtgt
cttttaactactatggcatgatcactggtcctcccgcttcaaagatttgc
...
>MRNA:0610010K06Rik.bSep07
gggccgccatagctacagtggcattcggccttggtttggggaccccaggc
tacatctctagggcccagacagaagctcaagaggtaagagcagacgaagt
...
```

**fig.5** AceView x1.all_mrnas_fasta.1.fasta file.

Download ensemlb cDNA file from Ensembl ftp site into data/ensdata and unzip it.

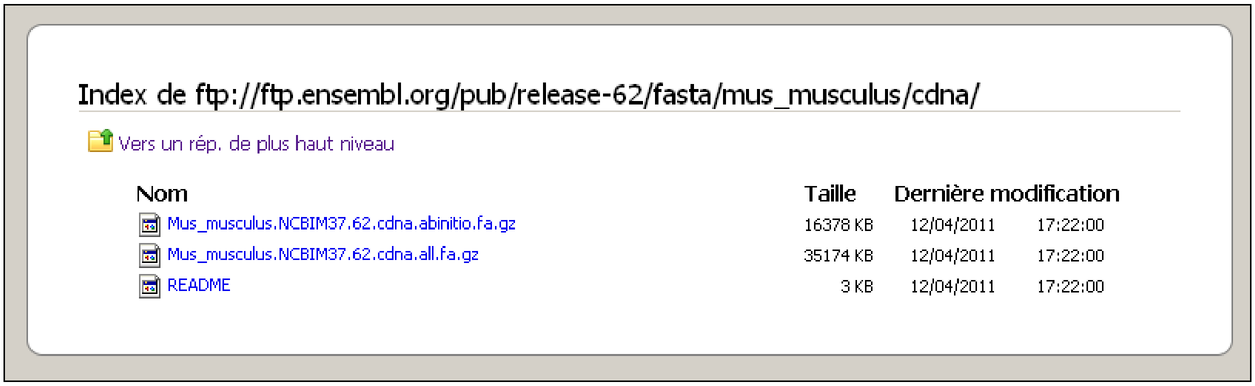


**fig.6** Download file Mus_musculus.NCBIM37.cdna.all.fa.gz from Ensembl ftp server.

```
>ENSMUST00000081908 cdna:known chromosome:NCBIM37:12:67384368:67385388:-1 gene:ENSMUSG00000060499
GCGGCTGGCGGTGGGCACGCACGGCTGAGGTTCCTGCTGTCGCCATGGGTCGTCGTCCGG
CTCGCTGTTACCGGTACTGTAAGAACAAGCCGTACCCAAAGTCCCGTTTCTGCCGAGGCG
TCCCCGATGCGAAGATCCGCATCTTTGACCTGGGTCGGAAGAAGGCGAAGGTGGATGAGT
TTCCACTCTGCGGCCACATGGTGTCGGACGAGTATGAGCAGCTCTCCTCGGAAGCCCTGG
AGGCCGCACGCATCTGCGCCAACAAGTACATGGTGAAGAGCTGTGGCAAGGACGGCTTCC
ACATCCGCGTGCGGCTGCACCCCTTCCATGTCATCCGCATCAACAAGATGCTGTCGTGCG
CAGGTGCCGACAGGCTCCAGACCGGCATGCGAGGCGCCTTCGGGAAACCACAAGGTACGG
TGGCGAGAGTCCACATCGGCCAGGTCATCATGTCCATCCGCACCAAGCTGCAGAACAAGG
AGCACGTGATCGAGGCCCTACGCCGAGCCAAATTCAAGTTCCCCGGCCGCCAGAAGATCC
```

**fig.7** Ensembl Mus_musculus.NCBIM37.cdna.all.fa.gz file in version 62.

## 1.4 User data

Some information on chips and on probes or on probesets must be given by user. A rather complete txt file is supplied which can be edited for specific purpose:

| myName | name | shortName | mySpeciesName | ProbeSetNb | probeNb | compName | ens47Name | ens48Name | geoName |
|---|---|---|---|---|---|---|---|---|---|
| m1 | Human Full Length HuGeneFL Array | HuGeneFL | human | 7129 | 20 | Affymetrix | HuGeneFL | HuGeneFL | GPL80 |
| m2 | Human Genome U95A Array | HG_U95A | human | 12626 | 16 | Affymetrix | HG_U95A | HG-U95A | GPL91 |
| m3 | Human Genome U133A Array | HG-U133A | human | 22283 | 11 | Affymetrix | HG_U133A | HG-U133A | GPL96 |
| m4 | Murine 11K SubA Array | Mu11ksubA | mouse | 6584 | 20 | Affymetrix | Mu11ksubA | Mu11ksubA | GPL75 |
| m5 | Murine Genome U74 Version 2 Array | MG_U74Av2 | mouse | 12488 | 16 | Affymetrix | MG_U74Av2 | MG-U74Av2 | GPL81 |
| m6 | Rat Genome U34 Array | RG_U34A | rat | 8799 | 16 | Affymetrix | RG-U34A | RG-U34A | GPL85 |
| m8 | Mouse Genome 430 2.0 Array | Mouse430_2 | mouse | 45101 | 11 | Affymetrix | Mouse430_2 | Mouse430_2 | GPL1261 |
| m10 | Arabidopsis Genome Array | AG | arabidopsis | 8297 | 16 | Affymetrix | AG | AG | GPL71 |
| m11 | Arabidopsis ATH1 Genome Array | ATH1-121501 | arabidopsis | 22810 | 11 | Affymetrix | ATH1-121501 | ATH1-121501 | GPL198 |
| m13 | C. elegans Genome Array | Celegans | worm | 22625 | 11 | Affymetrix | Celegans | Celegans | GPL200 |
| m14 | Drosophila Genome Array | DrosGenome1 | fly | 14010 | 14 | Affymetrix | DrosGenome1 | DrosGenome | GPL72 |
| m15 | Drosophila Genome 2.0 Array | Drosophila_2 | fly | 18955 | 14 | Affymetrix | Drosophila_2 | Drosophila_2 | GPL1322 |
| m17 | E. coli Genome Array | Ecoli | escherichia | 7312 | 15 | Affymetrix | Ecoli | Ecoli | GPL73 |
| m18 | E. coli Antisense Genome Array | Ecoli_ASv2 | escherichia | 7312 | 11 | Affymetrix | Ecoli_ASv2 | Ecoli_ASv2 | GPL199 |
| m21 | Human HG-Focus Target Array | HG-Focus | human | 8793 | 11 | Affymetrix | HG_Focus | HG-Focus | GPL201 |
| m22 | Human Genome U133A 2.0 Array | HG-U133A_2 | human | 22277 | 11 | Affymetrix | HG_U133A_2 | HG-U133A_2 | GPL571 |
| m24 | Human Genome U133 Plus 2.0 Array | G-U133_Plus_ | human | 54675 | 11 | Affymetrix | G_U133_Plus_ | G-U133_Plus_ | GPL570 |
| m26 | Murine Genome U74A Array | MG-U74A | mouse | 12654 | 16 | Affymetrix | MG_U74A | MG-U74A | GPL32 |
| m27 | Mouse Expression 430A Array | MOE430A | mouse | 22690 | 11 | Affymetrix | MOE430A | MOE430A | GPL339 |

**fig.8** chip.txt file supplied in data/rawdata. The first line is given to indicate the column order that must be respected, but does not belong to the file. All the fields are recovered into Chip class, but only the blue marked ones are important and used by the program. myName must respect the syntax given here (mx) (any name in a future version), but shortName must be the name used in the probe sequence file obtained from the supplier (e.g. Mouse430_2.probe_tab).

Take care to fill correctly the chip names used by Ensembl which are dependant of the version number. The following MySQL commands allow to recover the names used in Ensembl tables.

```
**up to version nb 47**
USE mus_musculus_core_47_37;
SELECT name,type FROM oligo_array
ORDER BY name

**from version nb 48**
USE mus_musculus_funcgen_62_37o;
SELECT name,class FROM array
ORDER BY name
```

Another file must describe species names.

| myName | officialName |
|---|---|
| arabidopsis | Arabidopsis thaliana |
| escherichia | Escherichia coli |
| fly | Drosophila melanogaster |
| human | Homo sapiens |
| mouse | Mus musculus |
| rat | Rattus norvegicus |
| rice | Oryzum sativa |
| worm | Caenorhabditis elegans |
| yeast | Saccharomyces cerevisiae |

**fig.9** species.txt file supplied in data/rawdata. myName must match mySpeciesName used in chip.txt. The first line is given to indicate the column order that must be respected, but does not belong to the file.

Each used chip model must have a file listing its probesets. For example probeset of m8 chip model are listed in m8_probeset.txt in data/rawdata.

```
1415670_at
1415671_at
1415672_at
1415673_at
1415674_a_at
1415675_at
1415676_a_at
...
```

**fig.10** Probe set ids in m8_probeset.txt.

Finally, the file signal_vs_rank.txt is needed to convert ranks (the unit spanning range 0-100 used to store chip results in our system) into signals, needed to calculate Pearson's correlation coefficients between probesets targeting the same gene in PSAWNml.

| rank | signal |
|---|---|
| 0.00000 | 0.005 |
| 0.00801 | 0.300 |
| 1.00889 | 2.300 |
| 2.00977 | 3.300 |
| 3.01065 | 4.200 |
| ... | ... |
| 97.09344 | 2078.200 |
| 98.09432 | 2870.000 |
| 99.09520 | 4485.400 |
| 99.99199 | 26347.000 |
| 100.00000 | 876749.800 |

**fig.11** signal_vs_rank.txt file supplied in data/rawdata. The first line is given to indicate the column order that must be respected, but does not belong to the file.

## 1.5 PSAWNpy

All the application can be run in command line by using psawn.py. At each step control can be made with ps_test.py.

### 1.5.1 Importation of user data

- construct chip database (=> data/pydata/common/chip.bkdb)

```
python psawn.py -a 1

python ps_test.py -a 1 -i "['m4','m5','m8','m26','m27','m48','m49','m50','m53','m54','m62','m65','m6

***** chip m4 (rank 21)
        myName: m4
        name: Murine 11K SubA Array
        shortName: Mu11ksubA
        mySpeciesName: mouse
        probesetNb: 6584
        probeNb: 20
```

```
        compName: Affymetrix
        ens47Name: Mu11ksubA
        ens48Name: Mu11ksubA
        geoName: GPL75

***** chip m5 (rank 29)
        myName: m5
        name: Murine Genome U74 Version 2 Array
        shortName: MG_U74Av2
        mySpeciesName: mouse
        probesetNb: 12488
        probeNb: 16
        compName: Affymetrix
        ens47Name: MG_U74Av2
        ens48Name: MG-U74Av2
        geoName: GPL81

***** chip m8 (rank 50)
        myName: m8
        name: Mouse Genome 430 2.0 Array
        shortName: Mouse430_2
        mySpeciesName: mouse
        probesetNb: 45101
        probeNb: 11
        compName: Affymetrix
        ens47Name: Mouse430_2
        ens48Name: Mouse430_2
        geoName: GPL1261

.....
```

- construct species database (=> data/pydata/common/species.bkdb)

```
python psawn.py -a 2

python ps_test.py -a 2 -i "['mouse','human']" -r ""

***** species mouse (rank 4)
        myName: mouse
        officialName: Mus musculus

***** species human (rank 3)
        myName: human
        officialName: Homo sapiens
```

- construct probeset databases (=> data/pydata/common/chip_probeset.bkdb & => data/pydata/mouse/ensembl/['m4','m5','m8','m26','m27','m48','m49','m50','m53','m54','m62','m65','m67','m70']_probeset.b

```
python psawn.py -a 3 -i ['m4','m5','m8','m26','m27','m48','m49','m50','m53','m54','m62','m65','m67','

python ps_test.py -a 3 -c m8 -i "['1415670_at']" -r ""

+++++ probeset 1415670_at (rank 0)

        probesetID: 1415670_at
        probesetIndex: 0
```

### 1.5.2 Importation of species information

#### Importation of Ensembl data

- Construct probe database (=> data/pydata/mouse/ensembl/m8_probes.bkdb)

```
python psawn.py -a 4 -c m8 -e 62_37o

python ps_test.py -a 4 -c m8 -i "['0683:0877']"

***** probe 0683:0877
        probeID: 0683:0877
        ensemblID: 3958134
        ensProbesetID: 100405
```

- Fill probeset with Ensembl gene identificator(s) (=> data/pydata/mouse/ensembl/m8_probeset.bkdbk)

```
python psawn.py -a 5 -c m8 -e 62_37o

python ps_test.py -a 5 -c m8 -i "['1415670_at']" -r ""

+++++ probeset 1415670_at (rank 0)

        probesetID: 1415670_at
        probesetIndex: 0
        ensGeneIDs: None
```

- Import Affymetrix informations (=> data/pydata/mouse/ensembl/m8_probeset.bkdb & => data/pydata/mouse/ensembl/m8_probe.bkdb)

```
python psawn.py -a 6 -c m8

python ps_test.py -a 6 -c m8 -i 1415670_at
or (because only one probeset is displayed)
python ps_test.py -a 6 -c m8 -r 0

+++++ probeset 1415670_at (rank 0)

        probesetID: 1415670_at
        probesetIndex: 0
        affyGeneIDs: none
        probeNb: 11
        probeIDs: ['0269:0753', '0486:0557', '0780:0603', '0479:0899', '0653:0375', '0070:0475', '01
        ensemblProbeIDs: [2991140L, 2991142L, 2991137L, 2991136L, 2991139L, 2991144L, 2991141L, 29911
        probeIndexes: [0, 1, 2, 3, 4, 5, 6, 7, 8, 9, 10]
        probesetTargetLength: 545
        probesetTargetStart: 2424
        probesetTargetEnd: 2968


***** probe 0269:0753
        probeID: 0269:0753
        ensemblID: 2991140
        ensProbesetID: 3770
        probesetID: 1415670_at
        index: 0
        sequence: GGCTGATCACATCCAAAAAGTCATG
        targetPosition: 2436
```

```
***** probe 0486:0557
        probeID: 0486:0557
        ensemblID: 2991142
        ensProbesetID: 3770
        probesetID: 1415670_at
        index: 1
        sequence: GAGGAAACGTTCACCCTGTCTACTA
        targetPosition: 2513

***** probe 0780:0603
        probeID: 0780:0603
        ensemblID: 2991137
        ensProbesetID: 3770
        probesetID: 1415670_at
        index: 2
        sequence: GTTCACCCTGTCTACTATCAAGACA
        targetPosition: 2521

.....
```

- Import Ensembl list of transcripts for each exon (=> data/pydata/m8/ensembl/m8_transcripts_by_exon.bkdb)

```
python psawn.py -a 7 -s mouse -e 62_37o

python ps_test.py -a 7 -s mouse -i "['ENSMUSE00000738026']" -r ""


***** exon ENSMUSE00000738026
        Transcript IDS: ['ENSMUST00000133383']
```

- Import Ensembl list of exons by gene (=> data/pydata/mouse/ensembl/mouse_exons_by_gene.bkdb)

```
python psawn.py -a 8 -s mouse -e 62_37o

python ps_test.py -a 8 -i "['ENSMUSG00000000049']" -s mouse -r ""

***** gene ENSMUSG00000000049:
        exon.IDs = ['ENSMUSE00000738026', 'ENSMUSE00000821986', 'ENSMUSE00000837885', 'ENSMUS[illegible]
        exon.indexes = [0, 1, 2, 3, 4, 5, 6, 7, 8, 9, 10, 11, 12, 13, 14, 15]
        exon.groups = [0, 1, 1, 2, 2, 2, 3, 3, 3, 4, 5, 6, 6, 7, 8, 9]
        exon.exonStarts = [108204668 108242478 108242483 108256607 108256616 108256619 108257[illegible]
108257130 108257130 108258613 108266125 108268605 108268605 108270445
108273309 108275554]
        exon.exonEnds = [108204838 108242710 108242710 108256721 108256721 108256721 10825716[illegible]
108257219 108257306 108258709 108266201 108268653 108268793 108270624
108273506 108275710]
        exon.strands = [1 1 1 1 1 1 1 1 1 1 1 1 1 1 1 1]
        exon.intronStarts = [108204839 108242711 108256722 108257165 108258710 108266202 1082[illegible]
108270625 108273507]
        exon.intronEnds = [108242477 108256606 108257129 108258612 108266124 108268604 108270[illegible]
108273308 108275553]
        exon.transcriptIDs = ['ENSMUST00000000049', 'ENSMUST00000146050', 'ENSMUST00000133383'[illegible]
        exon.transcriptStarts = [108256607 108242478 108204668 108242483]
        exon.transcriptEnds = [108270624 108257219 108257306 108275710]
        exon.transcriptList of exon 0 = set(['ENSMUST00000133383'])
        exon.transcriptList of exon 1 = set(['ENSMUST00000146050'])
        exon.transcriptList of exon 2 = set(['ENSMUST00000152958'])
        .....
```

- Import Ensembl list of regions (=> data/pydata/mouse/ensembl/genes_by_region.bkdb & data/pydata/mouse/ensembl/region_by_gene.bkdb)

```
python psawn.py -a 9 -s mouse -e 62_37o

python ps_test.py -a 9  -i "['ENSMUSG00000000049']" -s mouse -q 1 -r ""

***** gene ENSMUSG00000000049 (rank 5)
        region: 1
```

- Import Ensembl list exons by predicted transcrips (=> data/pydata/ensembl/mouse_exons_by_predicted_transcript.bkdb)

```
python psawn.py -a 10 -e 62_37o -s mouse

python ps_test.py -a 10  -i "['18616','18608']" -r "" -s mouse

***** predicted transcript 18616 (rank 9575)
        exon.IDs = [126399L, 126401L, 126403L, 126405L, 126406L, 126408L, 126409L]
        exon.indexes = [0, 1, 2, 3, 4, 5, 6]
        exon.exonStarts = [3023946 3027482 3028932 3029230 3029385 3029768 3030156]
        exon.exonEnds = [3024127 3027647 3029121 3029339 3029523 3029914 3030316]
        exon.strands = [1 1 1 1 1 1 1]

***** predicted transcript 18608 (rank 9566)
        exon.IDs = [126380L, 126378L, 126377L, 126376L, 126375L, 126374L, 126373L, 126372L, 12
        exon.indexes = [28, 27, 26, 25, 24, 23, 22, 21, 20, 19, 18, 17, 16, 15, 14, 13, 12, 11
        exon.exonStarts = [3032030 3032219 3032890 3033075 3034321 3035657 3035946 3036628 303
3040140 3042311 3043263 3043698 3044220 3044795 3049871 3050099 3050319
3050620 3056775 3057869 3065579 3070590 3077430 3079400 3086068 3087243
3096607 3102207]
        exon.exonEnds = [3032134 3032330 3032968 3033210 3034545 3035833 3036020 3036702 30371
3040236 3042413 3043339 3043793 3044295 3044973 3050069 3050274 3050533
3050914 3056867 3057925 3065681 3070707 3077524 3079510 3086139 3087413
3096787 3102342]
        exon.strands = [-1 -1 -1 -1 -1 -1 -1 -1 -1 -1 -1 -1 -1 -1 -1 -1 -1 -1 -1 -1 -1 -1 -1 -
-1 -1 -1 -1]
```

- Import Ensembl list of predicted transcripts by region (=> data/pydata/ensembl/mouse_predicted_transcript_by_region.bkdb)

```
python psawn.py -a 11 -e 62_37o -s mouse

python ps_test.py -a 11 -i "[18616,18608]" -q 1 -r "" -s mouse

***** predicted transcript 18616
        region = 1
        transcript.start = 3023946
        transcript.end = 3030316
        transcript.strand = 1

***** predicted transcript 18608
        region = 1
        transcript.start = 3032030
        transcript.end = 3102342
        transcript.strand = -1
```

- Import Ensembl transcripts by gene (=> data/pydata/mouse/ensembl/transcripts_by_gene.bkdb)

```
python psawn.py -a 12 -s mouse -e 62_37o

python ps_test.py -a -i "['ENSMUSG00000000049']" -q 1 -r "" -s mouse
```

```
***** gene ENSMUSG00000000049 (rank 5)
        transcript.IDs = ['ENSMUST00000000049', 'ENSMUST00000133383', 'ENSMUST00000146050', 'ENSMUST(
```

- Import Ensembl region by chromosome (=> data/pydata/ensembl/mouse_chromosome_by_region.bkdb & data/pydata/ensembl/mouse_region_by_chromosome.bkdb)

```
python psawn.py -a 13 -e "62_37o" -s mouse

python ps_test.py -a 13 -s mouse

***** region => chromosome
        1 => 11
        10 => 6
        11 => X
        .....

***** chromosome => region
        1 => 6
        10 => 21
        11 => 1
        .....
```

- Import Ensembl transcript sequences (=> data/pydata/mouse/ensembl/transcript_sequence.bkdb)

```
python psawn.py -a 14 -e 62_37o -s mouse

python ps_test.py -a 14 -i "['ENSMUST00000000090']" -s  -r "mouse"

***** transcript ENSMUST00000000090 (rank 9)
        transcript.ID=ENSMUST00000000090
        transcript.geneID=ENSMUSG00000000088
        transcript.start=57369039
        transcript.end=57380231
        transcript.region=14
        transcript.chromosome=9
        transcript.strand=1
        transcript.sequence=GTCGCTGCGTGAGTCCGGCCCCCGCGAACT ...
```

### Importation of AceView data

- Import AceView transcript sequences (=> data/pydata/mouse/aceview/mouse_transcript_sequence.bkdb)

```
python psawn.py -a 15 -s mouse -v Sep07

python ps_test.py -a 15 -i "['0610007C21Rik.hSep07']" -r "" -s mouse

***** transcript 0610007C21Rik.hSep07 (rank 9)
        transcript.ID=0610007C21Rik.hSep07
        transcript.geneID=0610007C21Rik
        transcript.start=31356243
        transcript.end=31357006
        transcript.region=22
        transcript.chromosome=5
        transcript.strand=1
        transcript.sequence=agaagaagaagaagaagaagaagaaagagg ...
```

- Import AceView transcipt information (=> data/pydata/mouse/aceview/mouse_exons_by_gene.bkdd &

data/pydata/mouse/aceview/mouse_genes_by_ensembl_region.bkdd & data/pydata/mouse/aceview/mouse_transcrits_by_exon.bk[illegible]
& data/pydata/mouse/aceview/mouse_transcrits_by_gene.bkdd)

```
python psawn.py -a 16 -s mouse -v Sep07
```

data/pydata/mouse/aceview/mouse_exons_by_gene.bkdd

```
python ps_test.py -a 16 -b a -i "['2210417D09Rik']" -s mouse -q 1 -r ""

***** gene 2210417D09Rik (rank 702)
        exonStarts= [146981055]
        exonEnds= [146982127]
        strands= [1]
        indexes = [0]
        groups= [0]
        intronStarts= []
        intronEnds= []
        transcriptIDs= ['a']
        transcriptStarts= [146981055]
        transcriptEnds= [146982127]
        exon.transcriptList of exon 0 = set(['a'])
```

data/pydata/mouse/aceview/mouse_genes_by_ensembl_region.bkdd

```
python ps_test.py -a 16 -b b -i "['2210417D09Rik']" -q 1 -r "" -s mouse

        gene ranks: [45]
        gene IDs: 2210417D09Rik
        starts: 146981055
        ends: 146982127
        strands: 1
```

data/pydata/mouse/aceview/mouse_transcrits_sequence.bkdd

```
python ps_test.py -a 16 -b c -i "['0610007C21Rik.hSep07']" -r "" -s mouse

***** transcript 0610007C21Rik.hSep07 (rank 9)
        transcript.ID=0610007C21Rik.hSep07
        transcript.geneID=0610007C21Rik
        transcript.start=31356243
        transcript.end=31357006
        transcript.region=22
        transcript.chromosome=5
        transcript.strand=1
        transcript.sequence=agaagaagaagaagaagaagaagaaagagg ...
```

data/pydata/mouse/aceview/mouse_transcrits_by_gene.bkdd

```
python ps_test.py -a 16 -b d -i "['2210417D09Rik']" -r "" -s mouse

***** gene 2210417D09Rik (rank 702)
        transcript.IDs = ['a']
```

data/pydata/mouse/aceview/mouse_transcrits_by_exon.bkdd

```
python ps_test.py -a 16 -b e -i "['0610006L08Rik.exon0']" -r "" -s mouse


***** exon 0610006L08Rik.exon0
        Transcript IDS: ['b']
```

- Find correspondance between AceView genes and Ensembl genes (data/pydata/mouse/aceview/ensembl_genes_by_gene.bkdd)

```
python psawn.py -a 17 -s mouse

python ps_test.py -a 17 -i "['2210417D09Rik']" -r "" -s mouse

***** gene 2210417D09Rik (rank 702)
        ['ENSMUSG00000040121']
```

### 1.5.3 Process chip information

- Find probe positions by region for a chip list (data/pydata/mouse/ensembl/m8_positions_by_region.bkdd)

```
python psawn.py -a 18 -e 62_37o -l "['m4','m5','m8','m26','m27','m48','m49','m50','m53','m54','m62','

python ps_test.py -a 18 -c m8 -i "['0683:0877']" -q 1 -r "[1, 10, 100]" -s mouse

+++++ region 1

        ***** position.probeID = 0716:0647 (rank 1)
        position.median = 3004894
        position.strand: 1
        position.mismatch: 1

        ***** position.probeID = 0146:0417 (rank 10)
        position.median = 3023580
        position.strand: -1
        position.mismatch: 0

        ***** position.probeID = 0683:0877 (rank 5)
        position.median = 3005111
        position.strand: 1
        position.mismatch: 0

        .....
```

- Merge probe positions of different chips (data/pydata/mouse/ensembl/mouse_positions_by_region.bkdd)

```
python psawn.py -a 19 -i "['m4','m5','m8','m26','m27','m48','m49','m50','m53','m54','m62','m65','m67

python ps_test.py -a 19 -c m8 -q 1 -r "[1, 10, 100]" -s mouse

+++++ region 1

        ***** position.probeID = set(['4305339']) (rank 1)
        position.chips = set([65])
        position.median = 3005358
        position.strand = -1
        position.mismatch = 1

        ***** position.probeID = set(['608576']) (rank 10)
        position.chips = set([65])
        position.median = 3011831
        position.strand = -1
        position.mismatch = 0

        ***** position.probeID = set(['6099283', '6096723']) (rank 100)
        position.chips = set([65])
```

```
position.median = 3030181
position.strand = -1
position.mismatch = 0
```

- Find group of probes (GOP) (data/pydata/mouse/ensembl/mouse_gop_by_region.bkdd)

```
python psawn.py -a 20 -s mouse -v Sep07

python ps_test.py -a 20 -c m8 -i "['GOPMUSG00000000086']" -q 1 -r "" -s mouse
```

```
+++++ gop.ID GOPMUSG00000000086 (rank 3)
gop.start = 4767619
gop.end = 4767619
gop.strand = -1
gop.probeNb = 1
gop.upGeneIDs = GOPMUSG00000000087
gop.upGeneDistances = 6190
gop.downGeneIDs = Nf2
gop.downGeneDistances = 13004
```

### process Ensembl information

- Update probe information for probes targeting genes (update data/pydata/mouse/ensembl/mouse_exons_by_gene.bkdb & data/pydata/mouse/ensembl/m8_probe.bkdb)

```
python psawn.py -a 21 -b a -c m8 -s mouse
```

- Update probe information for probes targeting GOPs (update data/pydata/mouse/ensembl/mouse_exons_by_gene.bkdb & data/pydata/mouse/ensembl/m8_probe.bkdb)

```
python psawn.py -a 22 -b a -c m8 -s mouse
```

- Assign probesets to ensembl genes (update data/pydata/mouse/m8_probeset.bkdb & data/pydata/mouse/ensembl/m8_probe.bkdb

```
python psawn.py -a 23 -c m8 -s mouse
```

### process AceView information

- Update probe information for probes targeting genes (update data/pydata/mouse/aceview/mouse_exons_by_gene.bkdb & data/pydata/mouse/aceview/m8_probe.bkdb)

```
python psawn.py -a 24-b a -c m8 -s mouse
```

- Assign probesets to aceview genes (update data/pydata/mouse/m8_probeset.bkdb & data/pydata/mouse/aceview/m8_probe.bkdb

```
python psawn.py -a 25 -c m8 -s mouse
```

- Updated probe and probeset files (data/pydata/mouse/m8_probeset.bkdb & data/pydata/mouse/ensembl/m8_probe.bkdb

```
python psawn.py -a 23 -c m8 -s mouse

python ps_test.py -a 23 -b b -c m8 -i 1415670_at

or (because only one probeset is displayed)
```

```
python ps_test.py -a 23 -b b -c m8 -r 0

+++++ probeset 1415670_at (rank 0)

        probesetID: 1415670_at
        probesetIndex: 0
        affyGeneIDs: none
        probeNb: 11
        probeIDs: ['0269:0753', '0486:0557', '0780:0603', '0479:0899', '0653:0375', '0070:0475', '01
        ensemblProbeIDs: [2991140L, 2991142L, 2991137L, 2991136L, 2991139L, 2991144L, 2991141L, 2991
        probeIndexes: [0, 1, 2, 3, 4, 5, 6, 7, 8, 9, 10]
        probesetTargetLength: 545
        probesetTargetStart: 2424
        probesetTargetEnd: 2968
        ensemblExonGeneNbs: [0, 0, 0, 0, 0, 0, 0, 0, 0, 0, 1, 0, 0, 0, 0, 0, 0, 0, 0, 0, 0]
        ensemblIntronGeneNbs: [0, 0, 0, 0, 0, 0, 0, 0, 0, 0, 0, 0, 1, 0, 0, 0, 0, 0, 0, 0, 0]
        ensemblUpGeneNbs: [0, 0, 0, 0, 0, 0, 0, 0, 0, 0, 0, 0, 0, 0, 0, 0, 0, 0, 0, 0, 0]
        ensemblDownGeneNb: [0, 0, 0, 0, 0, 0, 0, 0, 0, 0, 0, 0, 0, 0, 0, 0, 0, 0, 0, 1, 0]
        ensemblOutProbeNbs: [0, 0, 0, 0, 0, 0, 0, 0, 0, 0, 0, 0, 0, 0, 0, 0, 0, 0, 0, 0, 0]
        ensemblNisProbeNbs: [0, 0, 0, 0, 0, 0, 0, 0, 0, 0, 0, 0, 0, 0, 0, 0, 0, 0, 0, 0, 0]
        ourEnsemblGeneIDs: ['ENSMUSG00000030058']
        ourEnsemblGeneNb: 1
        ourEnsemblProbeNb: 11
        Ens: specific to ensemblGeneIds: []
        Ens: specific to ourEnsemblGeneIds: ['ENSMUSG00000030058']
        Ens: common to both: []
        aceExonGeneNbs: [0, 0, 0, 0, 0, 0, 0, 0, 0, 0, 0, 0, 1, 0, 0, 0, 0, 0, 0, 0, 0]
        aceIntronGeneNbs: [0, 0, 0, 0, 0, 0, 0, 0, 0, 0, 0, 0, 1, 0, 0, 0, 0, 0, 0, 0, 0]
        aceUpGeneNbs: [0, 0, 0, 0, 0, 0, 0, 0, 0, 0, 0, 0, 0, 0, 0, 0, 0, 0, 0, 0, 0]
        aceDownGeneNb: [0, 0, 0, 0, 0, 0, 0, 0, 0, 0, 0, 0, 0, 0, 0, 0, 0, 0, 0, 1, 0]
        aceOutProbeNbs: [0, 0, 0, 0, 0, 0, 0, 0, 0, 0, 0, 0, 0, 0, 0, 0, 0, 0, 0, 0, 0]
        aceNisProbeNbs: [0, 0, 0, 0, 0, 0, 0, 0, 0, 0, 0, 0, 0, 0, 0, 0, 0, 0, 0, 0, 0]
        ourAceGeneIDs: ['Copg']
        ourAceGeneNb: 1
        ourAceProbeNb: 9
        ourAceToEnsGeneIDs: ['ENSMUSG00000030058']
        ourAceToEnsGeneNbs: [1]
        Ace: specific to ensemblGeneIds: []
        Ace: specific to ourEnsemblGeneIds: ['ENSMUSG00000030058']
        Ace: common to both: []

        INFORMATION ON TARGETED GENES:

        ENSEMBL:

        key: 0

        ***** gene: ENSMUSG00000030058
        probeIndexes: [0, 1, 2, 3, 4, 5, 6, 7, 8, 9, 10]
        probeLocalisations: [['e', 'e', 'i'], ['e', 'e', 'i'], ['e', 'e', 'i'], ['s', 'i'], ['e', 'i'
        firstStructureIndexes: [[36, 38, 14], [36, 38, 14], [36, 38, 14], [-1, 14], [40, 15], [40, 15
        firstStructureGroups: [[14, 14, -1], [14, 14, -1], [14, 14, -1], [-1, -1], [15, -1], [15, -1]
        firstStructureIDs: [['ENSMUSE00000732550', 'ENSMUSE00000752188', -1], ['ENSMUSE00000732550',
        firstStructureGroups: [[14, 14, -1], [14, 14, -1], [14, 14, -1], [-1, -1], [15, -1], [15, -1]
        sndStructureIndexes: [[], [], [], [], [], [], [], [], [], [], []]
        probePositions: [[87859693, 87859693, 87859693], [87859769, 87859769, 87859769], [87859777, 8
        probeRepetitionNbs: [[1, 1, 1], [1, 1, 1], [1, 1, 1], [1, 1], [1, 1], [1, 1], [1, 1], [1, 1],
        probeStrands: [[1, 1, 1], [1, 1, 1], [1, 1, 1], [1, 1], [1, 1], [1, 1], [1, 1], [1, 1], [1, 1
```

```
probeMismatchNbs: [[0, 0, 0], [0, 0, 0], [0, 0, 0], [0, 0], [0, 0], [0, 0], [0, 0], [0, 0],
in ExonProbeNb: [2, 2, 2, 0, 1, 1, 1, 1, 0, 3, 3]
inSpliceProbeNb: [0, 0, 0, 1, 0, 0, 0, 0, 1, 0, 0]
inIntronProbeNb: [1, 1, 1, 1, 1, 1, 1, 1, 1, 0, 0]
upProbeNb: [0, 0, 0, 0, 0, 0, 0, 0, 0, 0, 0]
downProbeNb: [0, 0, 0, 0, 0, 0, 0, 0, 0, 0, 0]
exonSet: set([36, 38, 40, 42, 43, 44, 14, 15])
groupSet: set([16, -1, 14, 15])
targetedTranscripts: ['ENSMUST00000113607', 'ENSMUST00000127614', 'ENSMUST00000149907', 'ENSM
notTargetedtranscripts: ['ENSMUST00000049966', 'ENSMUST00000132938', 'ENSMUST00000137717', 'E
ensemblGenes: ['ENSMUSG00000030058']


***** gene: ENSMUSG00000030060
probeIndexes: [0, 1, 2, 3, 4, 5, 6, 7, 8, 9, 10]
probeLocalisations: [['o'], ['o'], ['o'], ['o'], ['o'], ['o'], ['o'], ['o'], ['o'], ['d'], ['
firstStructureIndexes: [[], [], [], [], [], [], [], [], [], [-1], [-1]]
firstStructureGroups: [[], [], [], [], [], [], [], [], [], [-1], [-1]]
firstStructureIDs: [[], [], [], [], [], [], [], [], [], [-1], [-1]]
firstStructureGroups: [[], [], [], [], [], [], [], [], [], [-1], [-1]]
sndStructureIndexes: [[], [], [], [], [], [], [], [], [], [], []]
probePositions: [[], [], [], [], [], [], [], [], [], [87862330], [87862480]]
probeRepetitionNbs: [[], [], [], [], [], [], [], [], [], [1], [1]]
probeStrands: [[], [], [], [], [], [], [], [], [], [1], [1]]
probeMismatchNbs: [[], [], [], [], [], [], [], [], [], [0], [0]]
in ExonProbeNb: [0, 0, 0, 0, 0, 0, 0, 0, 0, 0, 0]
inSpliceProbeNb: [0, 0, 0, 0, 0, 0, 0, 0, 0, 0, 0]
inIntronProbeNb: [0, 0, 0, 0, 0, 0, 0, 0, 0, 0, 0]
upProbeNb: [0, 0, 0, 0, 0, 0, 0, 0, 0, 0, 0]
downProbeNb: [0, 0, 0, 0, 0, 0, 0, 0, 0, 1, 1]
exonSet: set([])
groupSet: set([])
targetedTranscripts: None
notTargetedtranscripts: None
ensemblGenes: ['ENSMUSG00000030060']


key: 11

***** gene: ENSMUSG00000030058
probeIndexes: [0, 1, 2, 3, 4, 5, 6, 7, 8, 9, 10]
probeLocalisations: [['e', 'e', 'i'], ['e', 'e', 'i'], ['e', 'e', 'i'], ['s', 'i'], ['e', 'i'
firstStructureIndexes: [[36, 38, 14], [36, 38, 14], [36, 38, 14], [-1, 14], [40, 15], [40, 15
firstStructureGroups: [[14, 14, -1], [14, 14, -1], [14, 14, -1], [-1, -1], [15, -1], [15, -1]
firstStructureIDs: [['ENSMUSE00000732550', 'ENSMUSE00000752188', -1], ['ENSMUSE00000732550',
firstStructureGroups: [[14, 14, -1], [14, 14, -1], [14, 14, -1], [-1, -1], [15, -1], [15, -1]
sndStructureIndexes: [[], [], [], [], [], [], [], [], [], [], []]
probePositions: [[87859693, 87859693, 87859693], [87859769, 87859769, 87859769], [87859777, 8
probeRepetitionNbs: [[1, 1, 1], [1, 1, 1], [1, 1, 1], [1, 1], [1, 1], [1, 1], [1, 1], [1, 1],
probeStrands: [[1, 1, 1], [1, 1, 1], [1, 1, 1], [1, 1], [1, 1], [1, 1], [1, 1], [1, 1], [1, 1
probeMismatchNbs: [[0, 0, 0], [0, 0, 0], [0, 0, 0], [0, 0], [0, 0], [0, 0], [0, 0], [0, 0],
in ExonProbeNb: [2, 2, 2, 0, 1, 1, 1, 1, 0, 3, 3]
inSpliceProbeNb: [0, 0, 0, 1, 0, 0, 0, 0, 1, 0, 0]
inIntronProbeNb: [1, 1, 1, 1, 1, 1, 1, 1, 1, 0, 0]
upProbeNb: [0, 0, 0, 0, 0, 0, 0, 0, 0, 0, 0]
downProbeNb: [0, 0, 0, 0, 0, 0, 0, 0, 0, 0, 0]
exonSet: set([36, 38, 40, 42, 43, 44, 14, 15])
groupSet: set([16, -1, 14, 15])
```

```
targetedTranscripts: ['ENSMUST00000113607', 'ENSMUST00000127614', 'ENSMUST00000149907', 'ENSM
notTargetedtranscripts: ['ENSMUST00000049966', 'ENSMUST00000132938', 'ENSMUST00000137717', 'E
ensemblGenes: ['ENSMUSG00000030058']


ACEVIEW:

key: 0

***** gene: 8430410A17Rik
probeIndexes: [0, 1, 2, 3, 4, 5, 6, 7, 8, 9, 10]
probeLocalisations: [['o'], ['o'], ['o'], ['o'], ['o'], ['o'], ['o'], ['o'], ['o'], ['d'], ['
firstStructureIndexes: [[], [], [], [], [], [], [], [], [], [-1], [-1]]
firstStructureGroups: [[], [], [], [], [], [], [], [], [], [-1], [-1]]
firstStructureIDs: [[], [], [], [], [], [], [], [], [], [-1], [-1]]
firstStructureGroups: [[], [], [], [], [], [], [], [], [], [-1], [-1]]
sndStructureIndexes: [[], [], [], [], [], [], [], [], [], [], []]
probePositions: [[], [], [], [], [], [], [], [], [], [87862330], [87862480]]
probeRepetitionNbs: [[], [], [], [], [], [], [], [], [], [1], [1]]
probeStrands: [[], [], [], [], [], [], [], [], [], [1], [1]]
probeMismatchNbs: [[], [], [], [], [], [], [], [], [], [0], [0]]
in ExonProbeNb: [0, 0, 0, 0, 0, 0, 0, 0, 0, 0, 0]
inSpliceProbeNb: [0, 0, 0, 0, 0, 0, 0, 0, 0, 0, 0]
inIntronProbeNb: [0, 0, 0, 0, 0, 0, 0, 0, 0, 0, 0]
upProbeNb: [0, 0, 0, 0, 0, 0, 0, 0, 0, 0, 0]
downProbeNb: [0, 0, 0, 0, 0, 0, 0, 0, 0, 1, 1]
exonSet: set([])
groupSet: set([])
targetedTranscripts: None
notTargetedtranscripts: None
ensemblGenes: ['ENSMUSG00000030060']


***** gene: Copg
probeIndexes: [0, 1, 2, 3, 4, 5, 6, 7, 8, 9, 10]
probeLocalisations: [['e', 'e', 'e', 'i'], ['e', 'e', 'i'], ['e', 'e', 'i'], ['i'], ['e', 'i'
firstStructureIndexes: [[44, 46, 47, 11], [44, 47, 11], [44, 47, 11], [11], [49, 12], [49, 12
firstStructureGroups: [[11, 11, 11, -1], [11, 11, -1], [11, 11, -1], [-1], [12, -1], [12, -1]
firstStructureIDs: [['Copg.exon44', 'Copg.exon46', 'Copg.exon47', -1], ['Copg.exon44', 'Copg.
firstStructureGroups: [[11, 11, 11, -1], [11, 11, -1], [11, 11, -1], [-1], [12, -1], [12, -1]
sndStructureIndexes: [[], [], [], [], [], [], [], [], [], [], []]
probePositions: [[87859693, 87859693, 87859693, 87859693], [87859769, 87859769, 87859769], [8
probeRepetitionNbs: [[1, 1, 1, 1], [1, 1, 1], [1, 1, 1], [1], [1, 1], [1, 1], [1, 1], [1, 1],
probeStrands: [[1, 1, 1, 1], [1, 1, 1], [1, 1, 1], [1], [1, 1], [1, 1], [1, 1], [1, 1], [1],
probeMismatchNbs: [[0, 0, 0, 0], [0, 0, 0], [0, 0, 0], [0], [0, 0], [0, 0], [0, 0], [0, 0],
in ExonProbeNb: [3, 2, 2, 0, 1, 1, 1, 1, 0, 2, 2]
inSpliceProbeNb: [0, 0, 0, 0, 0, 0, 0, 0, 0, 0, 0]
inIntronProbeNb: [1, 1, 1, 1, 1, 1, 1, 1, 1, 0, 0]
upProbeNb: [0, 0, 0, 0, 0, 0, 0, 0, 0, 0, 0]
downProbeNb: [0, 0, 0, 0, 0, 0, 0, 0, 0, 0, 0]
exonSet: set([12, 11, 44, 46, 47, 49, 50, 51])
groupSet: set([11, 12, 13, -1])
targetedTranscripts: ['a', 'c', 'd', 'l']
notTargetedtranscripts: ['b', 'e', 'f', 'g', 'h', 'i', 'j', 'k', 'n']
ensemblGenes: ['ENSMUSG00000030058']


key: 9
```

```
        ***** gene: Copg
        probeIndexes: [0, 1, 2, 3, 4, 5, 6, 7, 8, 9, 10]
        probeLocalisations: [['e', 'e', 'e', 'i'], ['e', 'e', 'i'], ['e', 'e', 'i'], ['i'], ['e', 'i'
        firstStructureIndexes: [[44, 46, 47, 11], [44, 47, 11], [44, 47, 11], [11], [49, 12], [49, 12
        firstStructureGroups: [[11, 11, 11, -1], [11, 11, -1], [11, 11, -1], [-1], [12, -1], [12, -1]
        firstStructureIDs: [['Copg.exon44', 'Copg.exon46', 'Copg.exon47', -1], ['Copg.exon44', 'Copg.
        firstStructureGroups: [[11, 11, 11, -1], [11, 11, -1], [11, 11, -1], [-1], [12, -1], [12, -1]
        sndStructureIndexes: [[], [], [], [], [], [], [], [], [], [], []]
        probePositions: [[87859693, 87859693, 87859693, 87859693], [87859769, 87859769, 87859769], [8
        probeRepetitionNbs: [[1, 1, 1, 1], [1, 1, 1], [1, 1, 1], [1], [1, 1], [1, 1], [1, 1], [1, 1],
        probeStrands: [[1, 1, 1, 1], [1, 1, 1], [1, 1, 1], [1], [1, 1], [1, 1], [1, 1], [1, 1], [1],
        probeMismatchNbs: [[0, 0, 0, 0], [0, 0, 0], [0, 0, 0], [0], [0, 0], [0, 0], [0, 0], [0, 0],
        in ExonProbeNb: [3, 2, 2, 0, 1, 1, 1, 1, 0, 2, 2]
        inSpliceProbeNb: [0, 0, 0, 0, 0, 0, 0, 0, 0, 0, 0]
        inIntronProbeNb: [1, 1, 1, 1, 1, 1, 1, 1, 1, 0, 0]
        upProbeNb: [0, 0, 0, 0, 0, 0, 0, 0, 0, 0, 0]
        downProbeNb: [0, 0, 0, 0, 0, 0, 0, 0, 0, 0, 0]
        exonSet: set([12, 11, 44, 46, 47, 49, 50, 51])
        groupSet: set([11, 12, 13, -1])
        targetedTranscripts: ['a', 'c', 'd', 'l']
        notTargetedtranscripts: ['b', 'e', 'f', 'g', 'h', 'i', 'j', 'k', 'n']
        ensemblGenes: ['ENSMUSG00000030058']


***** probe 0269:0753
        probeID: 0269:0753
        ensemblID: 2991140
        ensProbesetID: 3770
        probesetID: 1415670_at
        index: 0
        sequence: GGCTGATCACATCCAAAAAGTCATG
        targetPosition: 2436

        genes targeted in exons: ['Copg']
        first gene targeted in exons: Copg
        repetitions: [1, 1, 1]
        IDs: ['Copg.exon44', 'Copg.exon46', 'Copg.exon47']
        indexes: [44, 46, 47]
        groups: [11, 11, 11]
        positions: [87859693, 87859693, 87859693]
        strands: [1, 1, 1]
        mismatchNbs: [0, 0, 0]

        genes targeted in introns: ['Copg']
        first gene targeted in introns: Copg
        repetitions: [1]
        IDs: []
        indexes: [11]
        groups: []
        positions: [87859693]
        strands: [1]
        mismatchNbs: [0]

        outOfGeneNb: 0
        notInSequence:  None

***** probe 0486:0557
        probeID: 0486:0557
```

```
        ensemblID: 2991142
        ensProbesetID: 3770
        probesetID: 1415670_at
        index: 1
        sequence: GAGGAAACGTTCACCCTGTCTACTA
        targetPosition: 2513

        genes targeted in exons: ['Copg']
        first gene targeted in exons: Copg
        repetitions: [1, 1]
        IDs: ['Copg.exon44', 'Copg.exon47']
        indexes: [44, 47]
        groups: [11, 11]
        positions: [87859769, 87859769]
        strands: [1, 1]
        mismatchNbs: [0, 0]

        genes targeted in introns: ['Copg']
        first gene targeted in introns: Copg
        repetitions: [1]
        IDs: []
        indexes: [11]
        groups: []
        positions: [87859769]
        strands: [1]
        mismatchNbs: [0]

        outOfGeneNb: 0
        notInSequence:  None

***** probe 0780:0603
        probeID: 0780:0603
        ensemblID: 2991137
        ensProbesetID: 3770
        probesetID: 1415670_at
        index: 2
        sequence: GTTCACCCTGTCTACTATCAAGACA
        targetPosition: 2521

        genes targeted in exons: ['Copg']
        first gene targeted in exons: Copg
        repetitions: [1, 1]
        IDs: ['Copg.exon44', 'Copg.exon47']
        indexes: [44, 47]
        groups: [11, 11]
        positions: [87859777, 87859777]
        strands: [1, 1]
        mismatchNbs: [0, 0]

        genes targeted in introns: ['Copg']
        first gene targeted in introns: Copg
        repetitions: [1]
        IDs: []
        indexes: [11]
        groups: []
        positions: [87859777]
        strands: [1]
        mismatchNbs: [0]
```

```
outOfGeneNb: 0
notInSequence:  None
```

### 1.5.4 Export data

- Make a dump of m8_probeset.bkdb (pydata/mouse/m8_probeset.dump)

```
python psawn.py -a 26 -c m8 -s mouse
```

- Update probesets by gene files (pydata/mouse/ensembl/m8_probesets_by_gene.bkdb & pydata/mouse/aceview/m8_probesets_by_gene.bkdb)

```
python psawn.py -a 27-c m8 -s mouse
```

- Write probesets by gene files (pydata/mouse/txt/ensembl_m8_probesets_by_gene_xx.txt & pydata/mouse/txt/aceview_m8_probesets_by_gene_xx.txt)

```
python psawn.py -a 28-c m8 -s mouse
```

| Ensembl gene ID | Probe set IDs | Probe set ranks | Ensembl exon IDs | Exon ranks | Probes in exons | Last exon | Last group | Exon groups | Probes in groups | Targeted transcripts | Not targeted transcripts | Probes out of exons | Probes in gene |
|---|---|---|---|---|---|---|---|---|---|---|---|---|---|
| ENSMUSG00000000159 | {'1460214_at'} | [44506] | {{'ENSMUSE00000696983' } } | {[18 ] } | {[13 ] } | 18 | 13 | {[13 ] } | {[6 ] } | {[4]} | {[1, 2, 3, 5, 6]} | {[5]} | {[11]} |
| ENSMUSG00000000365 | {'1425709_at'} | [10014] | {{'ENSMUSE00000310652' 'ENSMUSE00000615517' } } | {[12 13 ] } | {[12 13 ] } | 37 | 37 | {[12 13 ] } | {[2 4 ] } | {[1, 2]} | {[]} | {[5]} | {[11]} |
| ENSMUSG00000000378 | {'1456290_x_at'} | [40584] | {{'ENSMUSE00000742872' 'ENSMUSE00000852207' 'ENSMUSE00000370189' } } | {[25 23 24 ] } | {[12 ] } | 25 | 12 | {[12 ] } | {[6 ] } | {[1, 2, 3]} | {[4, 5, 6, 7, 8]} | {[0]} | {[6]} |
| ENSMUSG00000000804 | {'1430403_at'} | [14708] | {{'ENSMUSE00000105744' } } | {[32 ] } | {[32 ] } | 35 | 34 | {[32 ] } | {[6 ] } | {[1, 2]} | {[]} | {[5]} | {[11]} |
| ENSMUSG00000000902 | {'1435856_x_at'} | [20161] | {{'ENSMUSE00000715013' 'ENSMUSE00000774208' 'ENSMUSE00000101968' 'ENSMUSE00000810891' } } | {[21 22 24 23 ] } | {[9 ] } | 24 | 7 | {[7 ] } | {[6 ] } | {[1, 2, 3, 5]} | {[4, 6, 7]} | {[0]} | {[6]} |
| ENSMUSG00000001168 | {'1425049_at'} | [9354] | {{'ENSMUSE00000463876' 'ENSMUSE00000883285' } } | {[10 9 ] } | {[7 ] } | 10 | 7 | {[7 ] } | {[6 ] } | {[1, 3]} | {[2]} | {[0]} | {[6]} |
| ENSMUSG00000002227 | {'1447376_at'} | [31681] | {{'ENSMUSE00000734279' } } | {[9 ] } | {[4 ] } | 31 | 21 | {[2 ] } | {[6 ] } | {[6]} | {[1, 2, 3, 4, 5, 7, 8]} | {[5]} | {[11]} |
| ENSMUSG00000002416 | {'1453806_at'} | [38100] | {{'ENSMUSE00000193828' 'ENSMUSE00000832861' 'ENSMUSE00000840741' 'ENSMUSE00000840077' } } | {[4 2 5 9 ] } | {[0 1 3 ] } | 14 | 4 | {[0 1 3 ] } | {[3 2 1 ] } | {[1, 2, 3, 5, 6]} | {[4]} | {[5]} | {[11]} |
| ENSMUSG00000002791 | {'1427931_s_at'} | [12236] | {{'ENSMUSE00000858265' 'ENSMUSE00000850590' } } | {[10 13 ] } | {[8 10 ] } | 13 | 10 | {[8 10 ] } | {[2 1 ] } | {[1, 2]} | {[]} | {[0]} | {[6]} |
| ENSMUSG00000003208 | {'1429733_at'} | [14038] | {{'ENSMUSE00000378280' } } | {[7 ] } | {[7 ] } | 7 | 7 | {[7 ] } | {[6 ] } | {[1]} | {[]} | {[5]} | {[11]} |

**fig.12** File ensembl_m8_probesets_by_gene_06.txt.

| AceView ID | Probe set IDs | Probe set ranks | AceView exon IDs | Probes in exons | Last exon | Last group | Exon groups | Probes in groups | Targeted transcripts | Not targeted transcripts | Probes out of exons | Probes in gene |
|---|---|---|---|---|---|---|---|---|---|---|---|---|
| A430107O13Rik | {'1447948_at'} | [32253] | {{'A430107O13Rik.exon2' 'A430107O13Rik.exon0' 'A430107O13Rik.exon1' } } | {[6 6 6 ] } | 32 | 25 | {[0 ] } | {[6 ] } | {[1, 3, 4, 5]} | {[2, 6, 7]} | {[5]} | {[11]} |
| A930009A15Rik | {'1453398_at'} | [37692] | {{'A930009A15Rik.exon6' 'A930009A15Rik.exon7' } } | {[4 2 ] } | 7 | 5 | {[4 5 ] } | {[4 2 ] } | {[1]} | {[2]} | {[0]} | {[6]} |
| AA792892 | {'1455830_s_at'} | [40124] | {{'AA792892.exon5' 'AA792892.exon4' } } | {[6 6 ] } | 5 | 4 | {[4 ] } | {[6 ] } | {[1, 2]} | {[]} | {[0]} | {[6]} |
| AU015228 | {'1457309_at'} | [41603] | {{'AU015228.exon0' } } | {[6 ] } | 0 | 0 | {[0 ] } | {[6 ] } | {[1]} | {[]} | {[0]} | {[6]} |
| Abhd13 | {'1428276_at'} | [12581] | {{'Abhd13.exon4' } } | {[6 ] } | 4 | 2 | {[2 ] } | {[6 ] } | {[1]} | {[2]} | {[0]} | {[6]} |
| Abi1and1200016E24Rik | {'1423178_at', '1452418_at'} | [7483, 36712] | {{'Abi1and1200016E24Rik.exon31' 'Abi1and1200016E24Rik.exon30' 'Abi1and1200016E24Rik.exon32' 'Abi1and1200016E24Rik.exon29' } {'Abi1and1200016E24Rik.exon6' 'Abi1and1200016E24Rik.exon8' } } | {[6 4 6 1 ] [1 6 ] } | 32 | 13 | {[13 ] [2 ] } | {[6 ] [6 ] } | {[1, 2, 3, 4, 5, 7], [10, 16]} | {[6, 8, 9, 10, 11, 12, 13, 14, 15, 16], [1, 2, 3, 4, 5, 6, 7, 8, 9, 11, 12, 13, 14, 15]} | {[0, 0]} | {[6, 6]} |
| Ablim1 | {'1460120_at'} | [44412] | {{'Ablim1.exon18' } } | {[6 ] } | 47 | 30 | {[12 ] } | {[6 ] } | {[15]} | 6, 7, 8, 9, 10, 1 | {[0]} | {[6]} |
| Acaa2 | {'1455061_a_at'} | [39355] | {{'Acaa2.exon6' } } | {[6 ] } | 22 | 10 | {[2 ] } | {[6 ] } | {[2, 3]} | {[1, 4, 5, 6]} | {[5]} | {[11]} |
| Adap_comp_sub.2 | {'1450894_a_at'} | [35188] | {{'Adap_comp_sub.2.exon0' } } | {[6 ] } | 0 | 0 | {[0 ] } | {[6 ] } | {[1]} | {[]} | {[0]} | {[6]} |
| Adck2andNdufb2 | {'1453806_at'} | [38100] | {{'Adck2andNdufb2.exon28' 'Adck2andNdufb2.exon36' 'Adck2andNdufb2.exon24' 'Adck2andNdufb2.exon32' 'Adck2andNdufb2.exon31' 'Adck2andNdufb2.exon30' } } | {[3 1 3 2 2 2 ] } | 41 | 13 | {[9 10 12 ] } | {[3 2 1 ] } | {[5, 6, 7, 8, 9, 14, 15, 17]} | {[1, 2, 3, 4, 10, 11, 12, 13, 16]} | {[5]} | {[11]} |

**fig.13** File aceview_m8_probesets_by_gene_06.txt.

- Write probeset lists (pydata/mouse/txt/m8_probesets_ensembl.txt & pydata/mouse/txt/m8_probesets_aceview.txt)

```
python psawn.py -a 29-c m8 -s mouse
```

| ENSEMBL | | ACEVIEW |
|---|---|---|
| [0, 1, 0, 0, 0, 0, 0, 0, 0, 0, 0, 0] | | [0, 0, 0, 1, 0, 0, 0, 0, 0, 0, 0, 0] |
| [1, 1, 0, 0, 0, 0, 0, 0, 0, 0, 0, 0] | | [1, 1, 0, 0, 0, 0, 0, 0, 0, 0, 0, 0] |
| [2, 1, 0, 0, 0, 0, 0, 0, 1, 1, 0, 0] | | [2, 1, 0, 0, 0, 1, 0, 0, 0, 0, 0, 0] |
| [3, 0, 1, 0, 0, 0, 0, 0, 0, 0, 0, 0] | | [3, 0, 1, 0, 0, 0, 0, 0, 0, 0, 0, 0] |
| [4, 1, 0, 0, 0, 0, 0, 0, 0, 0, 0, 0] | | [4, 0, 0, 1, 0, 0, 0, 0, 0, 0, 0, 0] |
| [5, 1, 0, 0, 0, 0, 0, 0, 0, 0, 0, 0] | | [5, 1, 0, 0, 0, 0, 0, 0, 0, 0, 0, 0] |
| [6, 1, 0, 0, 0, 0, 1, 0, 1, 0, 0, 0] | | [6, 0, 1, 1, 0, 0, 0, 0, 0, 0, 0, 0] |

**fig.14** Files m8_probesets_ensembl.txt and m8_probesets_aceview.txt. First column indicates probeset rank, following columns show the number of genes targeted by 11,10,9 ...1 probes.

- Write AceView genes (pydata/mouse/txt/mouse_ens_by_ace_gene.txt

```
python psawn.py -a 30 -s mouse
```

| AceView Gene ID | Ensembl Gene Ids |
|---|---|
| 0610006L08Rik | {} |
| 0610007C21Rik | {'ENSMUSG00000013622'} |
| 0610007L01Rik | {'ENSMUSG00000053094'} |
| 0610007N19Rik | {'ENSMUSG00000089317','ENSMUSG00000090401'} |
| 0610007P08Rikand9330134C04Rik | {'ENSMUSG00000021470'} |
| 0610007P14Rik | {'ENSMUSG00000021252'} |
| 0610007P22Rik | {'ENSMUSG00000015126'} |
| 0610008C08Rik | {'ENSMUSG00000079508'} |
| 0610008F07Rik | {'ENSMUSG00000046840'} |
| 0610009B22Rik | {'ENSMUSG00000007777'} |
| 0610009D07Rik | {'ENSMUSG00000037361'} |
| 0610009K11Rik | {'ENSMUSG00000041241'} |
| 0610009O03Rik | {'ENSMUSG00000001082','ENSMUSG00000036693'} |
| | |

**fig.15** Files mouse_aceview_genes.txt.

## 1.6 PSAWNml

Matlab programs.

### 1.6.1 Vocabulary

- **- bicluster** one or several genes targeted by one or several probesets
- **- paired probesets** probesets that target a common gene.
- **- similar probesets** paired probesets that are assumed to target in a given network the same transcript(s) following a test that uses positive (CORR) and negative (ANTI) correlation, and the p-value (PV) of the similarity of their neighbourhood.
- **- positive network** a network in which a given pair or probesets is similar.
- **- triangle** three similar probesets.
- **- group** several probesets that are considered as being all similar since they are gathered by merging triangles that have one edge in common.
- **- probe number limit** the minimal number of probes a probeset must have in a gene to be considered as targeting this gene .

### 1.6.2 Importation of data generated by PSAWNpy

#### import_targetnb

**import_targetnb** reads a text file generated by PSAWNpy and containing either Ensembl or eventually AceView informations (see fig. 13 of PSAWNpy tutorial), and creates a matrix indicating for each probeset the number of genes that have x probes targeting their exons with x>=1 and x<=n(max(probe nb)))

```
ModelRank=8;
DisplayFlag=1;
import_targetnb(ModelRank,DisplayFlag)
```

### FIG16/17/18

Caracteristics of Ensembl and AceView genes.

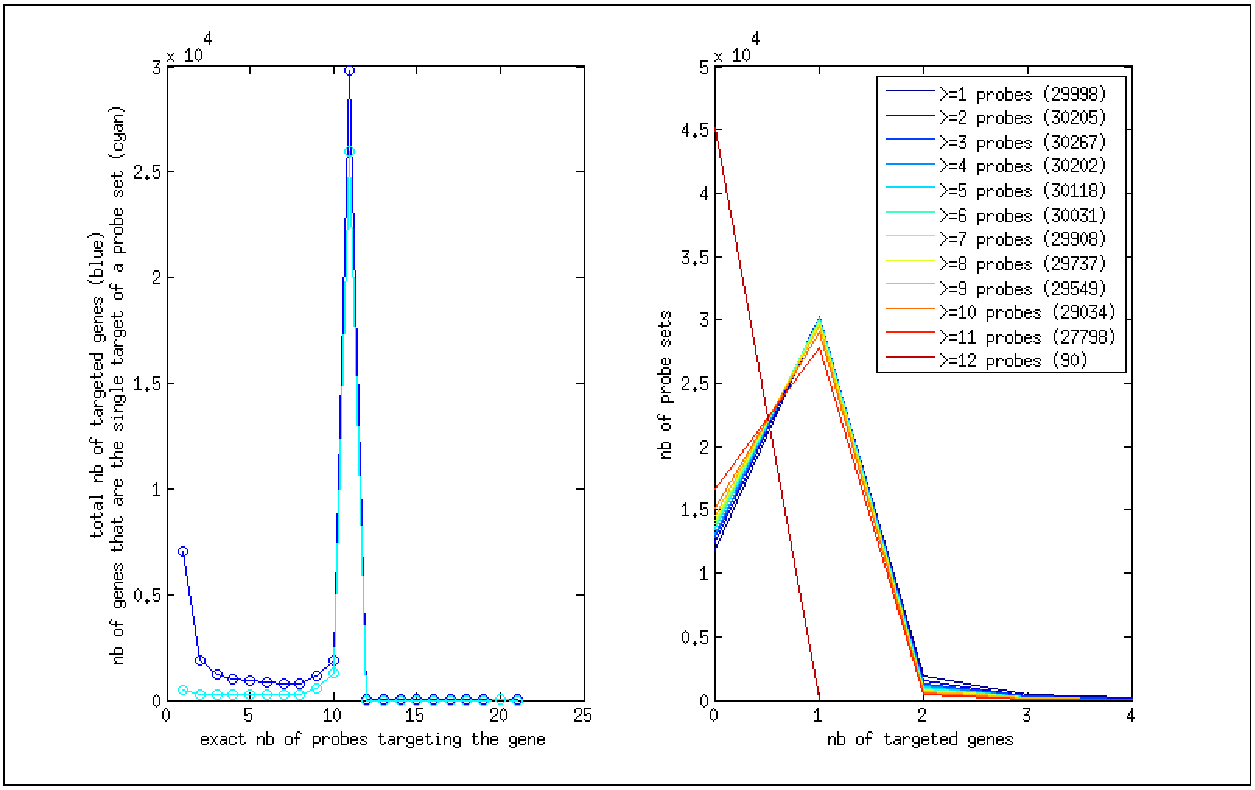


**fig.16 Probe sets targeting Ensembl genes**.On the *left panel*, are displayed the number of genes (blue) which are targeted by the number of probes indicated in abscissa (ProbeNbLimit variable). The cyan curve indicates the number of probesets which target these genes. Cyan and blue curves diverge as and when the number of probes reduces (lesser is the number of probes targeting a gene and higher is the number of targeted genes). On the *right panel*, the different curves indicates the number of probesets that target a given number of genes (here from 0 to 11 genes), according to the minimal number of probes allowed to define a target as indicated in the legend.

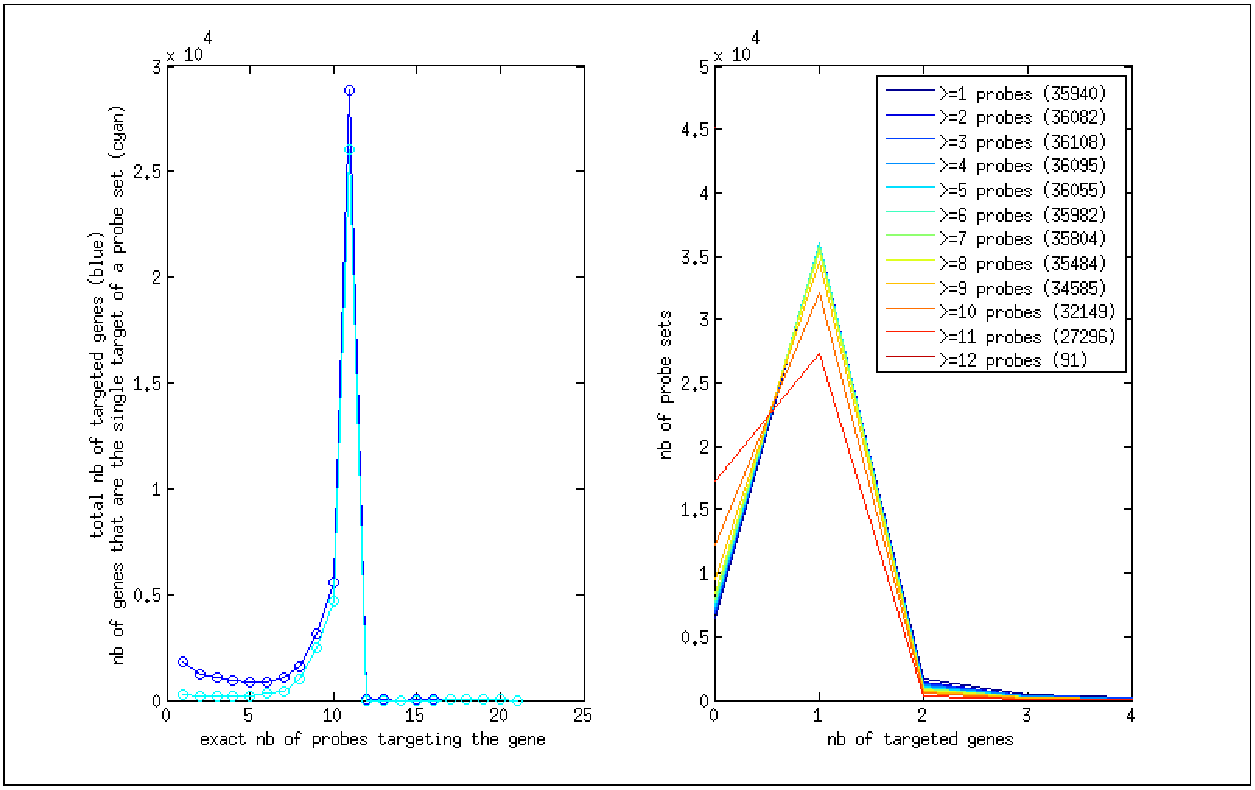


**fig.17 Probe sets targeting Aceview Genes**. It can be seen than the divergence between cyan and blue curves, for low values of targeting probes, is less important than those observed with Ensembl gene definitions (Fig. 1), and that the number of probesets that target a gene with 9 and 10 probes is higher.

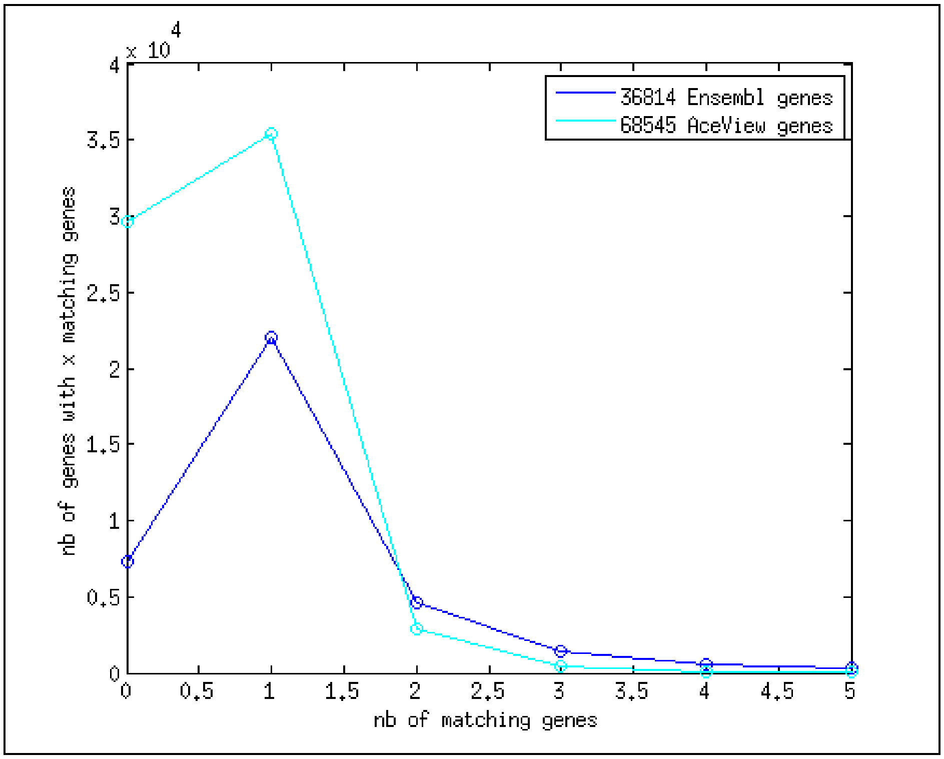


**fig.18 Correspondance between Ensembl and AceView genes**. The number of genes is largest AceView than in Ensembl. As a consequence about 50% of AceView genes have not counterpart in Ensembl. For the other genes, the relationship is complex, and it can be inferred from the relative position of blue and cyan curves, that many Ensembl genes overlap two or three AceView genes. This more stringeant definition of AceView genes (more genes, but many with a smaller size) could explain the differences between FIG15 and FIG16.

#### import_targetinfo

**import_targetinfo** reads a series of text files generated by PSAWNpy (see fig. 11-12 of PSAWNpy tutorial) that indicate for each gene the list of probesets that target it, with detailed information about the exons, group of exons and transcripts that are targeted.

```
ModelRank=8;
import_targetinfo(ModelRank)
```

### 1.6.3 Calculating the neighbourhood similariy on selected pairs of probesets

#### calculate_nodesim

**calculate_nodesim** calculates distribution of positive (CORR) and negative(ANTI) correlation, and of pv-value (PV) of overlap between neighbourhood for different categories of paired probesets in each networks of a series of networks.Categories are:

```
Pairs of probesets targeting a single gene
   Pairs of probesets inside exons of the same gene (Sim single)
   Pairs of probesets outside exons of the same gene (OutSim single)
   Pairs of randomly matched probesets present in Sim single and targeting genes with more
     or = than max(1,ProbeNbLimit-2 probes (HSim single)
   Pairs of randomly matched probesets present in Sim single targeting with genes with less
     than 3 probes (LSIm single)
   Pairs of randomly matched probesets, one present HSim single and the other in LSim single
     (LHSim single)
Pairs of probesets targeting several genes
   Pairs of probesets inside exons of the same genes (Sim multiple)
   Pairs of probesets outside exons of the same genes (OutSim multiple)
   Pairs of randomly matched probesets present in Sim multiple and targeting genes with more
     or = than max(1,ProbeNbLimit-2 probes (HSim multiple)
   Pairs of randomly matched probesets present in Sim multiple targeting with genes with less
     than 3 probes (LSim multiple)
   Pairs of randomly matched probesets, one present HSim multiple and the other in LSim multiple
     (LHSim multiple)
   Pairs of probesets inside exons of the same gene(s)
```

calculate_nodesim is used twice. In a first round (TestFlag=1), which is not mandatory, CORR, ANTI and PV distributions are calculated on all categories to study their differential properties;

```
TestFlag=1;
NotFoundFlag=0;
ProbeNbLimit=1;
ModelRank=8;
NetRankList=[7:21];
PvCorrList=[0,40,50,60];
calculate_nodesim(TestFlag,NotFoundFlag,ProbeNbLimit,ModelRank,NetRankList,PvCorrList)
```

In a second round (TestFlag=0), CORR, ANTI and PV distributions are calculated only on single category to find corresponding test limits used to determine if a particular pair of probeset must be considered as similar(that is targeting the same group of transcript(s)

```
TestFlag=0;
calculate_nodesim(TestFlag,NotFoundFlag,ProbeNbLimit,ModelRank,NetRankList,PvCorrList)
```

### display_nodesim

**display_nodesim** displays figures related to statistics calculated by calculate_nodesim (TestFlag=1) in one network.

```
TestFlag=1;
NotFoundFlag=0;
ProbeNbLimit=1;
Species='mouse';
ModelRank=8;
NetRank=7;
FigureRanks=[4:16];
AceViewFlag=1;
PvCorrList=[0,40,50,60];
display_nodesim(ProbeNbLimit,TestFlag,Species,Modelrank,NetRank,FigureRanks,1,PvCorrList)
```

### FIG19

Distributions of PV can be considered at different value of the CorrLimit parameter which allows to consider neigthbourhood of a probeset at different level of stringency (only probesets that have a positive correlation greater than CorrLimit are recruited in that neighbourhood). It can be seen that restricting neighbourhood to the highest correlated probesets, has an effect on PV which is shifted towards higher values.

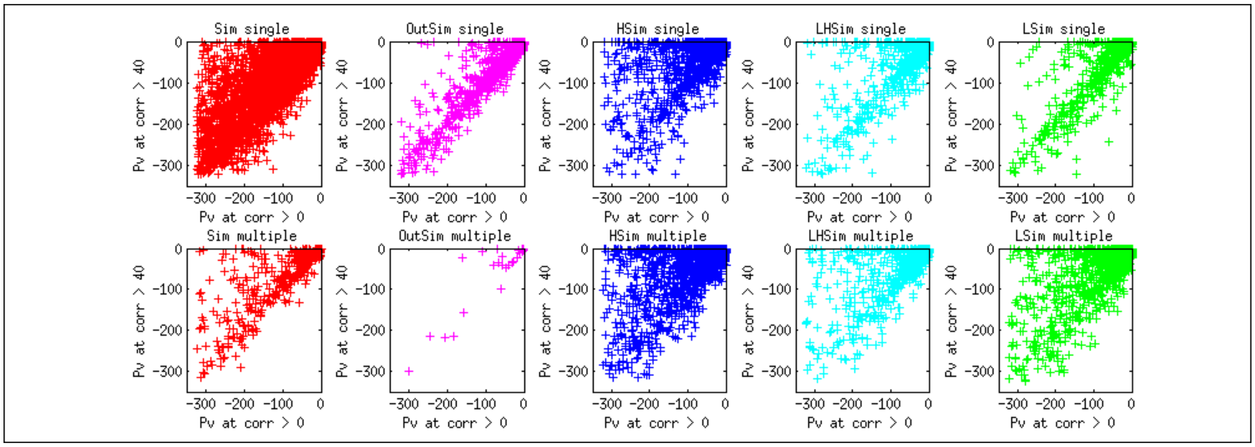


**fig.19a Effect of correlation limit on p-values of similarity: CorrLimit=40 vs CorrLimit=0**.

**fig.19b** Effect of correlation limit on p-values of similarity: CorrLimit=50 vs CorrLimit=0

**fig.19c** Effect of correlation limit on p-values of similarity: CorrLimit=60 vs CorrLimit=0

### FIG20

PV and CORR seems to have little correlation in this type of plot (but see FIG25).

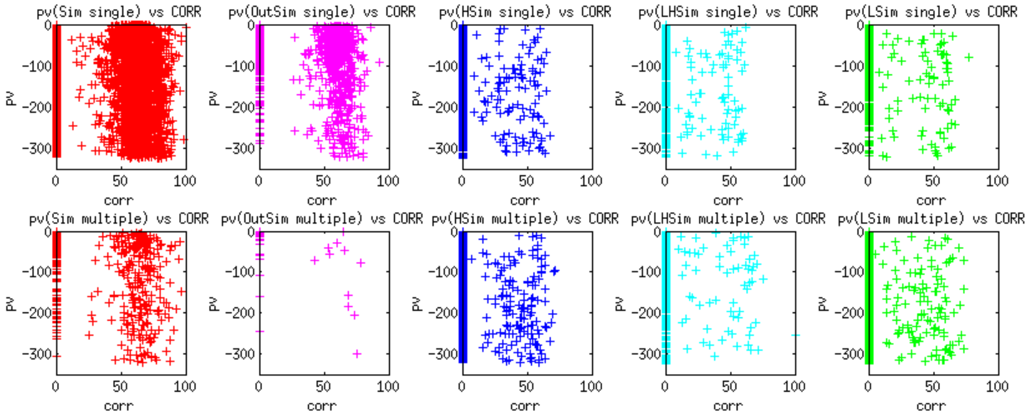


**fig.20a** **p-values of similarity vs correlation (CorrLimit=0)**.

**fig.20b** p-values of similarity vs correlation (CorrLimit=40)

**fig.20c** p-values of similarity vs correlation (CorrLimit=50)

**fig.20d** p-values of similarity vs correlation (CorrLimit=60)

### FIG21

Distribution of PV according to CorrLimit and to the strength of CORR between paired probesets. Sim and OutSim are true paired probesets that target respectively exons and introns or up and down seqences of a gene. HSim, LHSim and LSim are random probeset pairs. Single and multiple refers respectively to pairs that target a single of multiple genes. Pair of probesets that target an exon have a lower PV than those that target an intron, or the up or down sequence of the gene (red curves are always on top of magenta curves). However, the 95th percentile (the value that we use to test paired probeset) is not very different. Most of the randomly paired probesets have CORR=0 (for example in FIG21a, there are 10000 pairs in HSim single that have CORR>=0, and only 449 that have CORR>0). When CORR=0, these pairs have a higher PV than true paired probesets (blue, cyan and green green curves are low in CORR>=0 panel). However when we enforce that CORR>0, we select probesets that have very low PV(correlated probesets have a similar neighbourhood if CorrLimit=0; if CorrLimit=40, higher CORR values must be used).

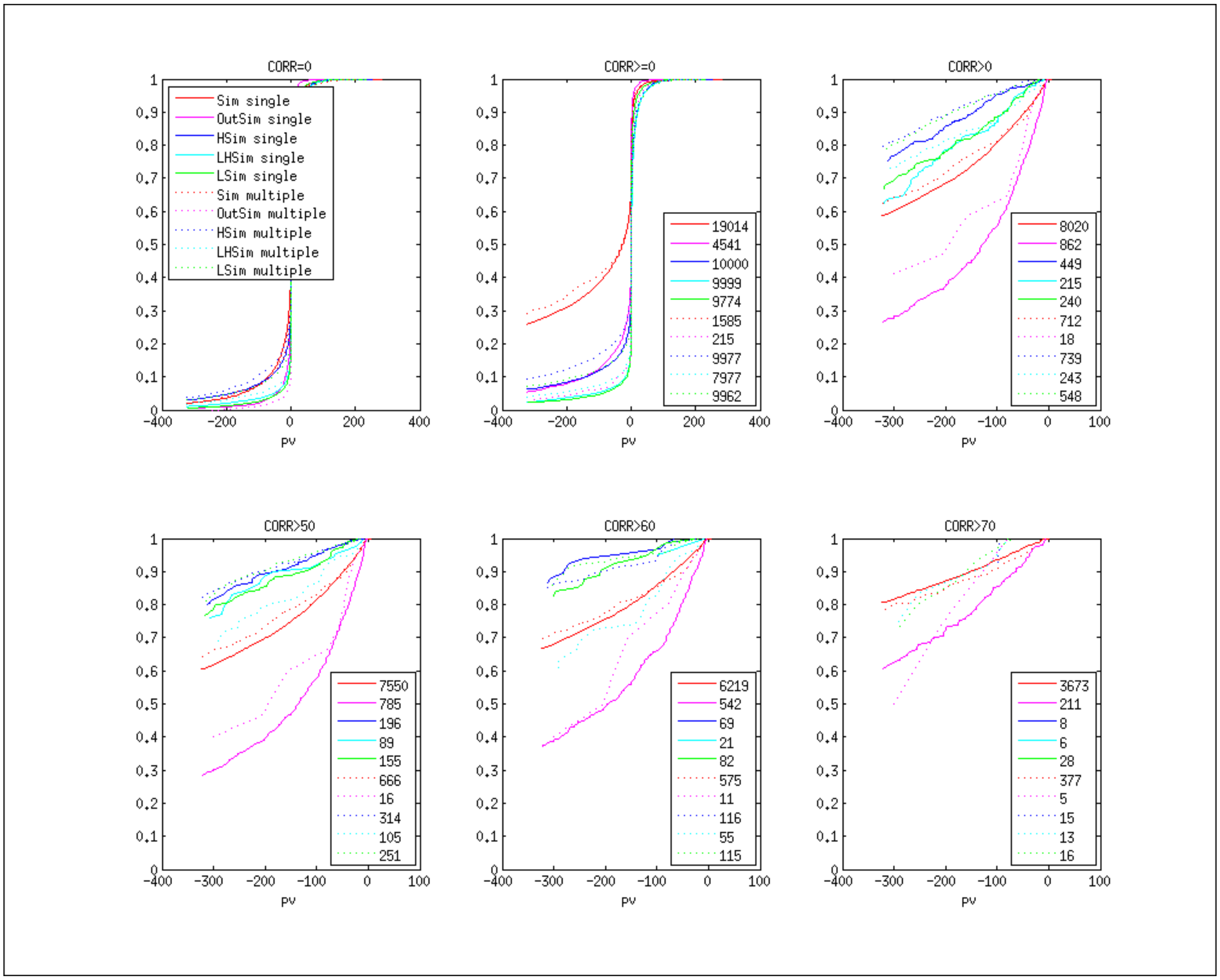


**fig.21a p-values distributions (CorrLimit=0)**.

**fig.21b** p-values distributions (CorrLimit=40)

**fig.21c** p-values distributions (CorrLimit=50)

**fig.21d** p-values distributions (CorrLimit=60)

### FIG22

Distribution of PV for CORR>0.

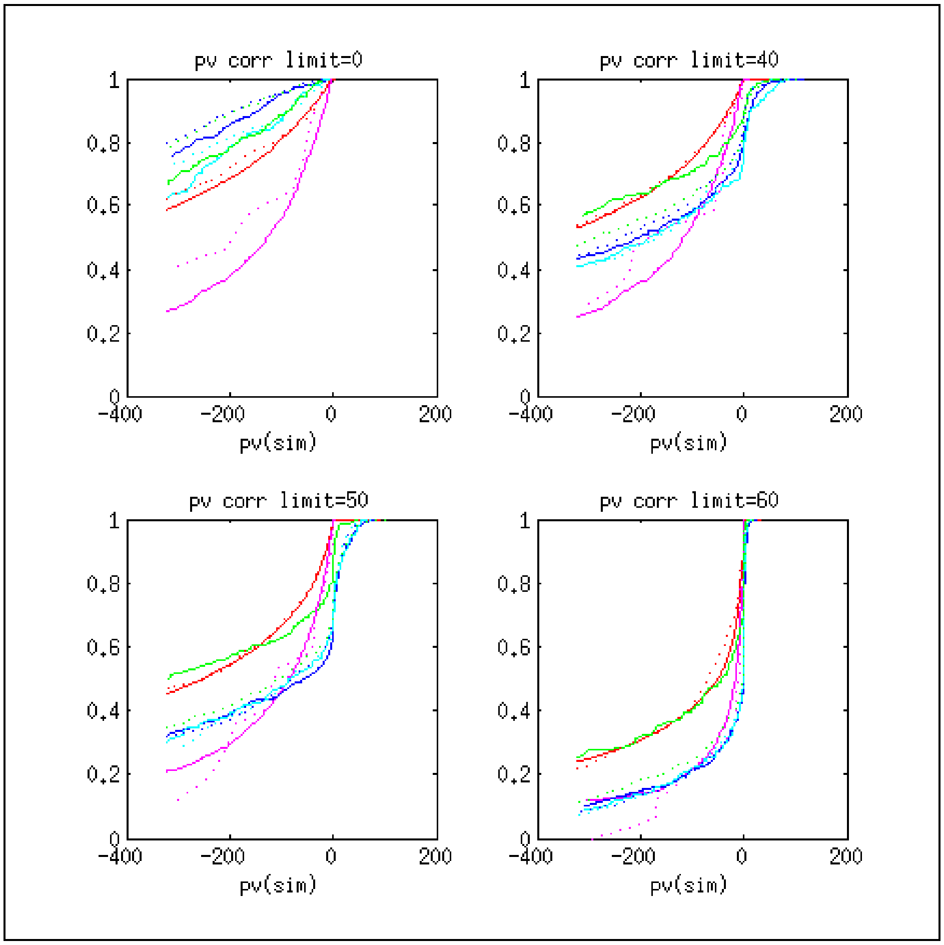


**fig.22 p-values distributions**.

### FIG23

Distribution of CORR, ANTI and CORR-ANTI for paired probesets with CORR>0. True paired probeset stand out from random ones. Sim and OutSim distributions are similar as are single and multiple distributions.

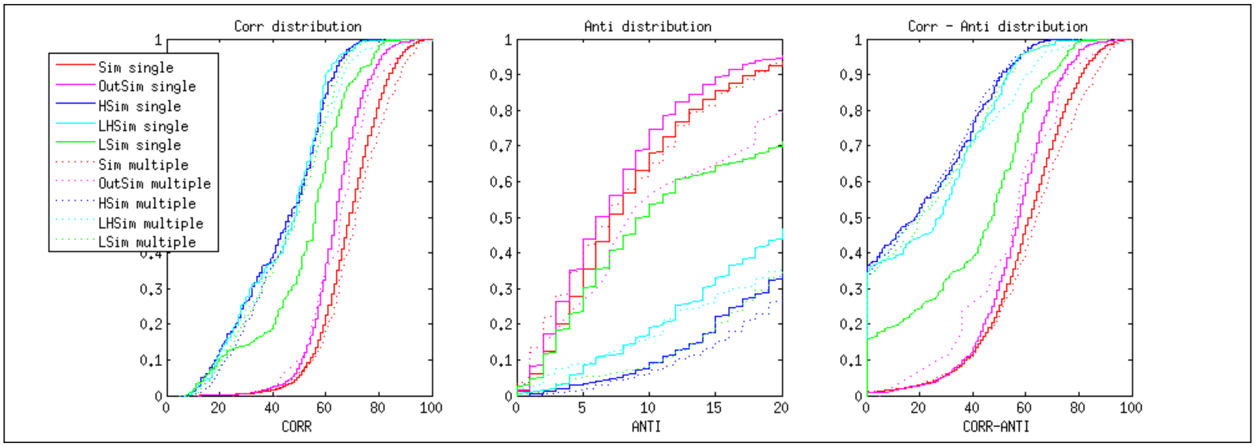


**fig.23 corr, anti and corr-anti distribution**.

### FIG24

Sim and OutSIm distributions of some caracteristics of paired probesets (number of common or uncommon genes or transcripts, ...).

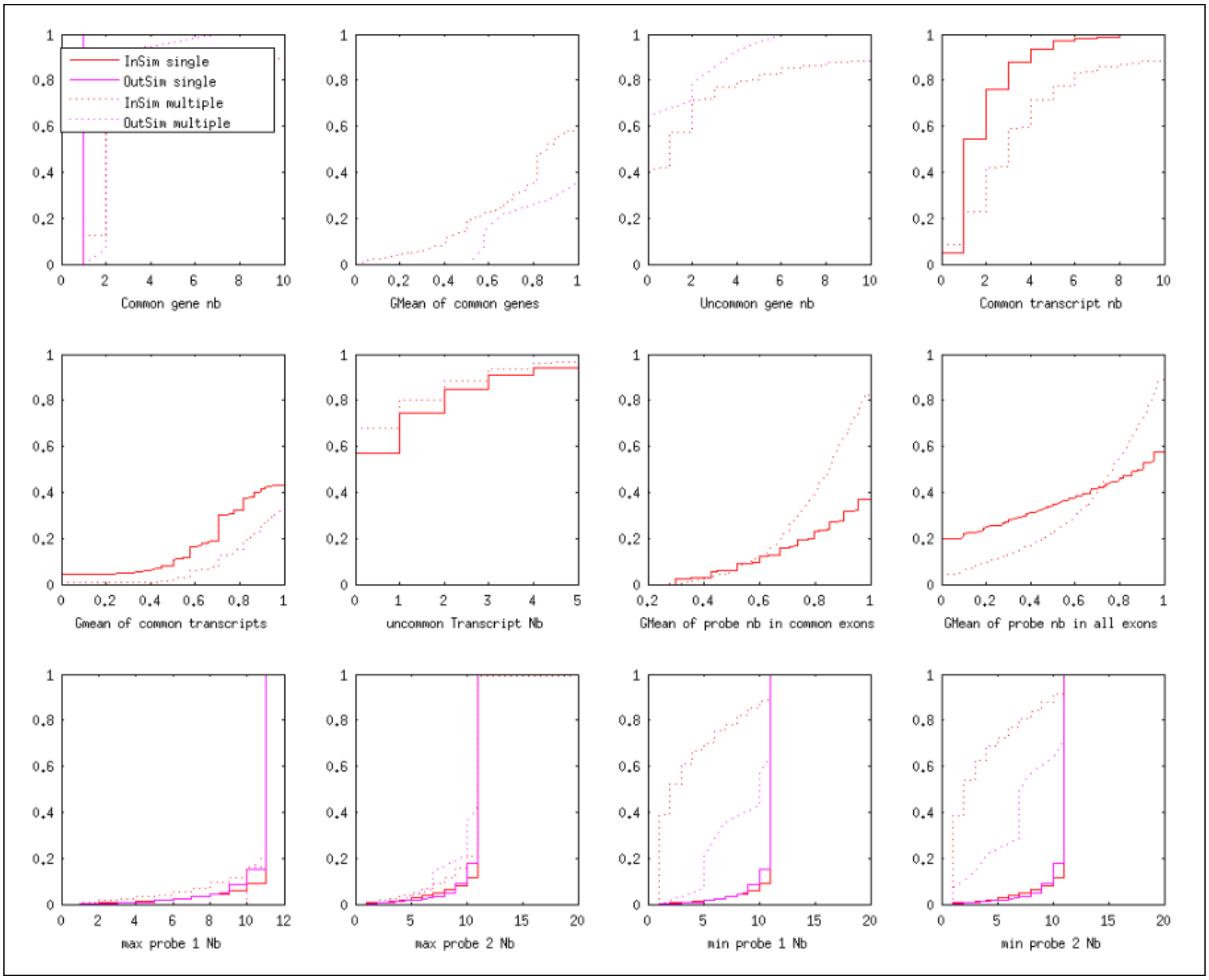


**fig.24 Caracteristics of InSim and OutSim**. GMean common items (genes or transcripts) are geometric means (#common/sqrt(#item1*#item2)). 'c mean probe': geometric mean of common probe nb relative to the number of probe targeting common exons in each probeset.'max(min) probe in 1(2)': greatest (lowest) number of targeting probe for the first (snd) probeset.

### FIG25

There is a clear relationship between high (low) CORR, low (high) ANTI and low (high) PV whivh appear when all values of one type are displayed after they have been ordered relatively to another one (for example CORR values indexed on ordered PV values).

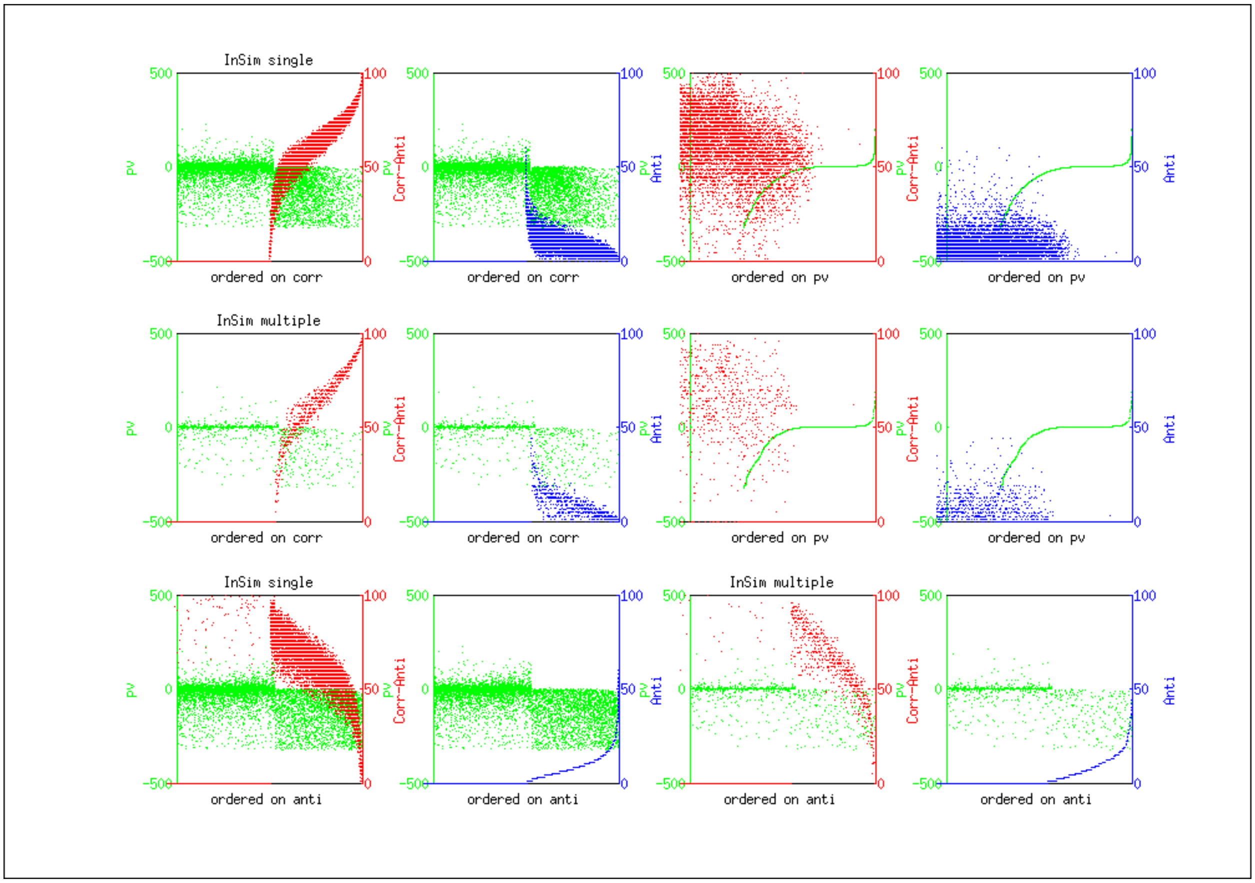


**fig.25 CORR, ANTI, PV plots in InSim**.

### FIG26

Paired probesets that are positively correlated have a higher mean number of neighbours, and a better overlap of their neighbourhood than paired probesets that are not correlated. Overlap distribution may have a bimodal distribution which is more visible with higher CorrLimit pvalues.

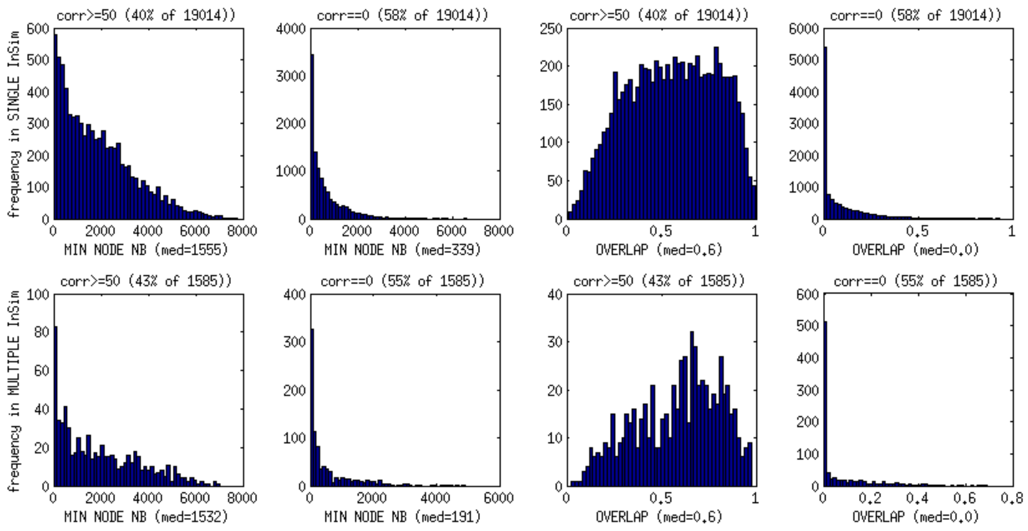


**fig.26a** **Statistics on nodes (CorrLimit=0)**. Is displayed the distribution of the smallest number of neighboors of two paired probesets (MIN NODE NB). The overlap (OVERLAP) between two neighbourhood is calculated as the fraction of common neighboors relative the smallest number of neighboors of the two paired probesets.

**fig.26b** Statistics on nodes (CorrLimit=40)

**fig.26c** Statistics on nodes (CorrLimit=50)

**fig.26d** Statistics on nodes (CorrLimit=60)

### FIG27

Paired probesets that are positively correlated have a higher mean number of neighbours, and a better overlap of their neighbourhood than paired probesets that are not correlated.

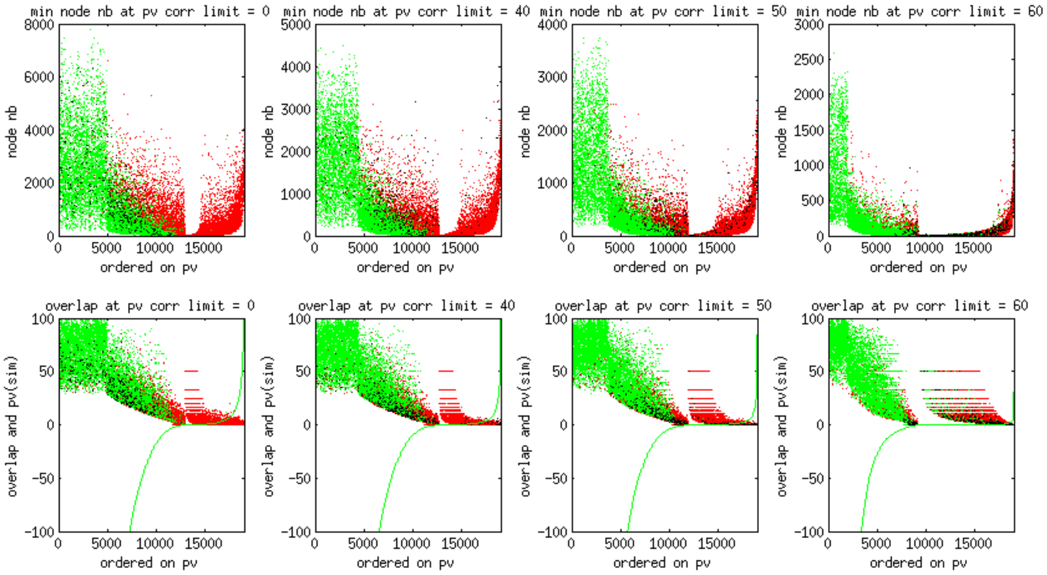


**fig.27a Statistics on nodes (InSim single)**. Number of neighbours (node nb), overlap calculated as explained in FIG26 (overlap) and PV (pv) are indexed on PV ordered values. Black, red and green points are paired probeset with CORR=0, with 0>CORR<50, and CORR>=50, respectively.

**fig.27b** Statistics on nodes (InSim multiple)

### FIG28

Frequency of paired probeset selected with different combination of CORR, ANTI and PV (InSim single) according to the smallest number of probes that target the gene (most of the probeset target genes with the modal (11) number of probes, that when the minimal number of probes for one probeset is less than 11, in general the other probeset target the gene with 11 probes).

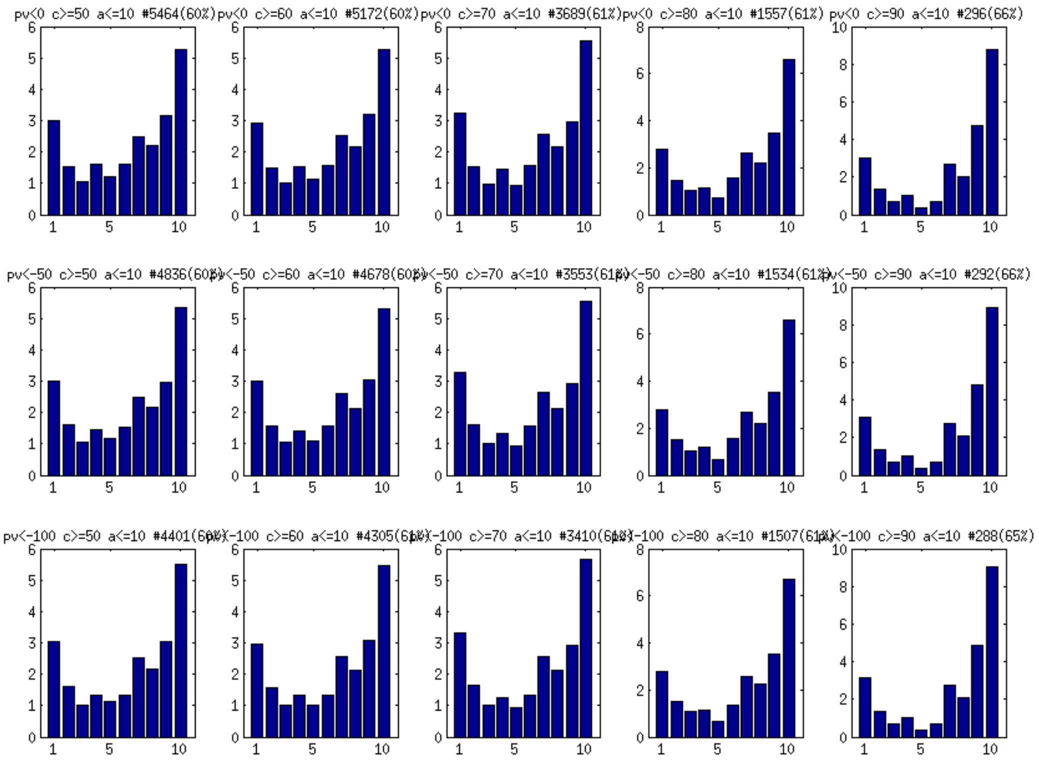

**fig.28a Frequency of paired probesets according to the number of targeting probes (InSim single).** In abscissa is plotted the smallest number of probes that target the gene in a pair of probesets (the bar corresponding to the modal number of probes (11) is very high in comparison with others and is not plotted (its value is given in parenthesis above the each figure)). The number of paired probesets corresponding to the selection is indicated by # symbol. pv,c and a stand respectively for PV, CORR, and ANTI.

**fig.28b** Frequency of paired probesets according to the number of targeting probes (InSim multiple)

### FIG29

**Enrichment of paired probesets selected with different combination of CORR, ANTI and PV values among probeset targeting exons of a single gene(InSim single category) according to the smallest number of probes that target the gene.** For probesets that target a single gene, below seven targeting probes, the observed frequency of paired probesets targeting the same group of transcript(s) (high CORR, low ANTI and low PV) is less than expected. This could indicate that if less than seven probes hybridize in one probeset of a pair targeting the same transcript(s), at least one of the CORR, ANTI or PV values tends to be affected, which prevent to detect the pair similarity. ore As we do not observe this effect in probesets pairs targeting multiple genes we could hypothesize that in this case, probes that do not target the assigned gene hybridize with other targeted genes, which prevent that one of the CORR, ANTI or PV value is affected.

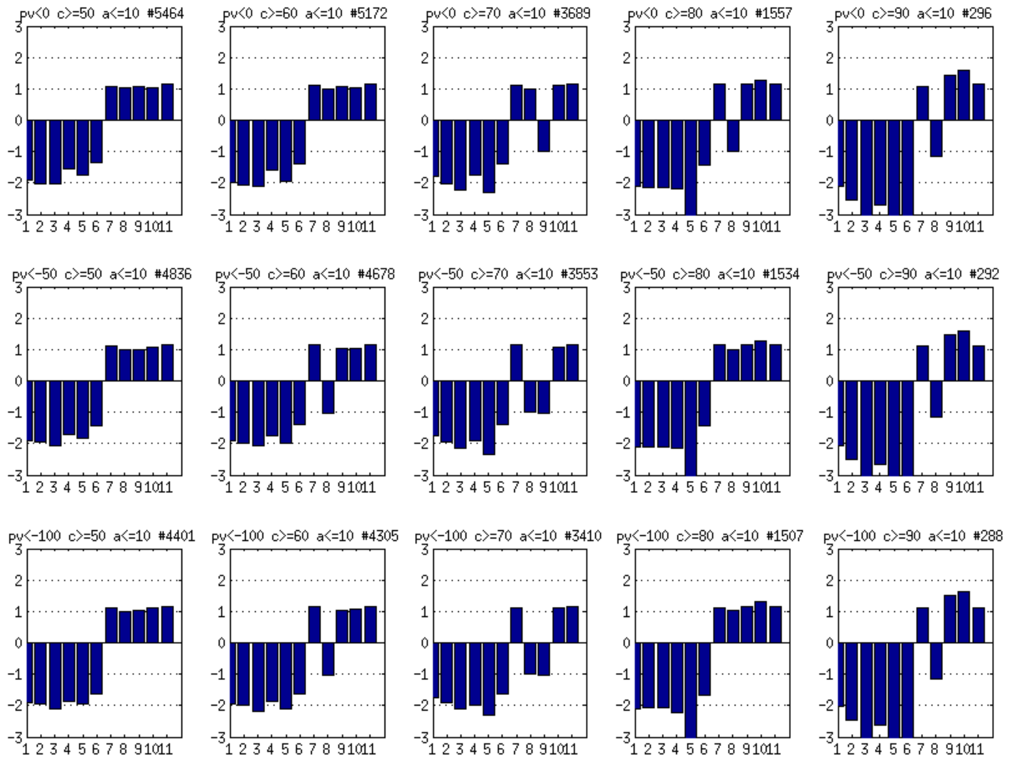


**fig.29a Enrichment of paired probesets according to the smallest number of targeting probes (InSim single category)**. Ratio between the frequency observed on the selected subgroup (FIG28) and the total frequency observed on the whole population for each value of targeting probe number. If the ratio is less than 1 (depletion), the negative inverse is plotted.

**fig.29b** Enrichment of paired probesets according to the number of targeting probes (In Sim multiple)

### calculate_limits

```
Species='mouse';
ChipRank=8;
ProbeNbLimit=1;
FigRanks=[15:27];
FirstNetRanks=[7:21];
PvCorrRank=1;
ValFlag=1;
MeanFlag=0;
[Limit]=calculate_limits(Species,ChipRank,ProbeNbLimit,FigRanks,FirstNetRanks,PvCorrRank,ValFlag,Mea[illegible]
```

Distributions are now envisionned in several networks.

## FIG30

Distribution of CORR, ANTI, PV according to the number of positive networks for CORR>0. Paired probesets are grouped according to the number of networks in which their CORR is greater than 0. We observe that distribution corresponding to the maximal number of networks (here 15), are apart of all the other curves which are ordered according to the number of positive networks. A network is positive for a given pair of probeset if CORR>0.

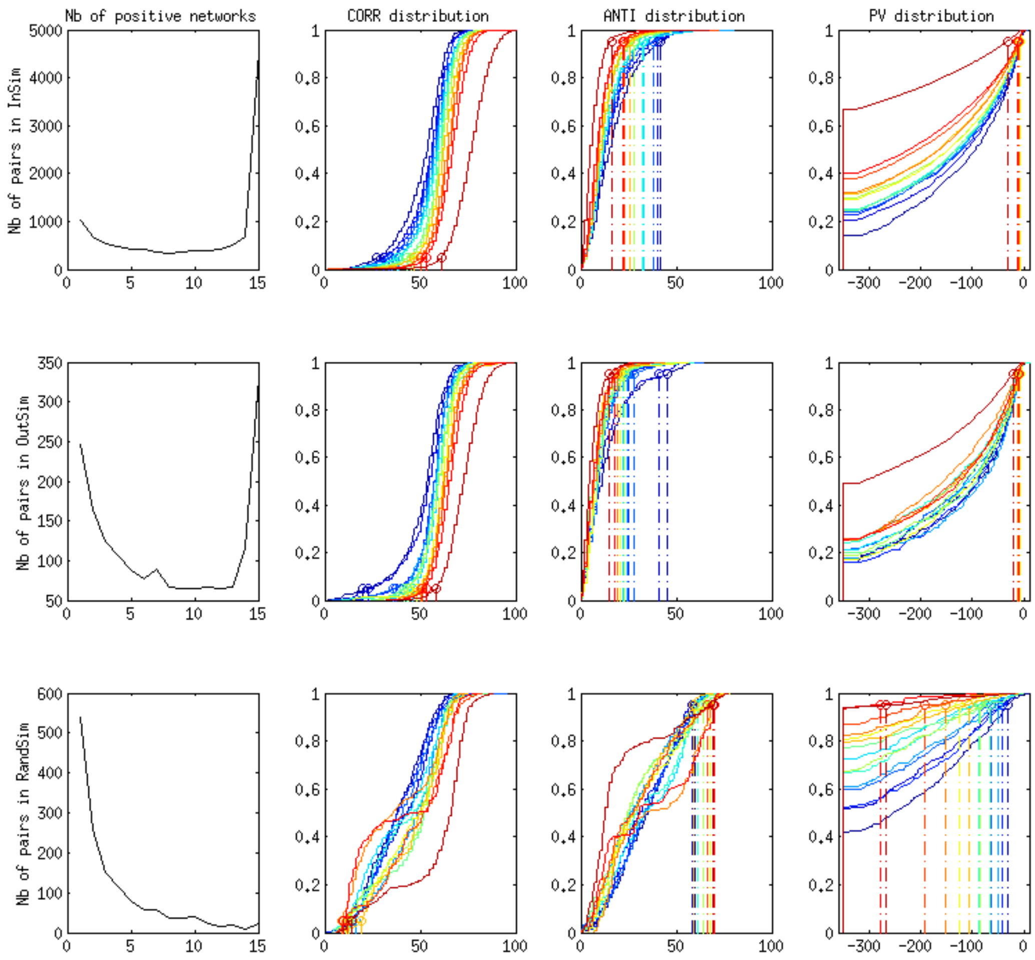


**fig.30a Statistics on CORR, ANTI and PV according to the number of positive networks (single).** Vertical lines indicate either 95th percentile (ANTI, PV) or 5th percentile (CORR).

**fig.30b** Statistics on CORR, ANTI and PV according to the number of positive networks (multiple)

### FIG31

Evolution of 5th percentile of CORR and 95th percentile of ANTI and PV according to the number of positive networks. Two ways for calculating these values are plotted. In the first method (FIG31a), each paired probeset give a single value (mean -std of all individual values observed in networks positive for this pair), and the percentiles are calculated on the final series of values. In the second method (FIG31b), all individual values are gathered, and the percentiles are calculated are calculated on the final series of these values. We found that second method gave more reproducible results between probeset paris targeting single and multiple genes. A network is positive for a given pair of probeset if CORR>0.

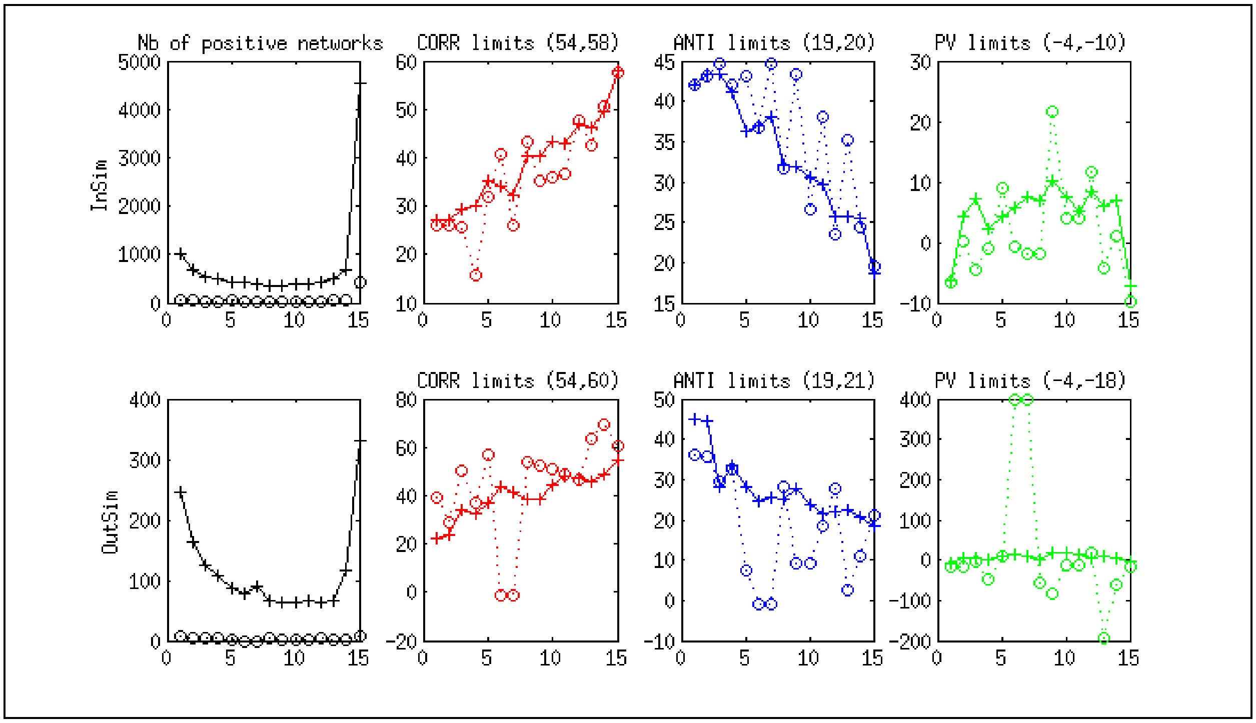


**fig.31a Limits on CORR,ANTI and PV (mean - std for all values of each paired probesets)**. Continuous and interrupted lines correspond to InSim single and InSim multiple respectively (repective limits calculated from percentiles of distributions relative to probeset pairs positive in all networks (which are finally used to test similarity of paired probeset) are in parenthesis).

**fig.31b** Limits on CORR,ANTI and PV (all values)

### FIG32

Distribution of CORR, ANTI and PV in different categories of paired probesets. Random pair of probesets that have by chance CORR>0 in one network may have a better PV than paired probesets, but their CORR and ANTI distribution are clearly shifted towards worse values. Moreover, these random pairs have CORR>0 in a small numbe of netwoks (FIG33).

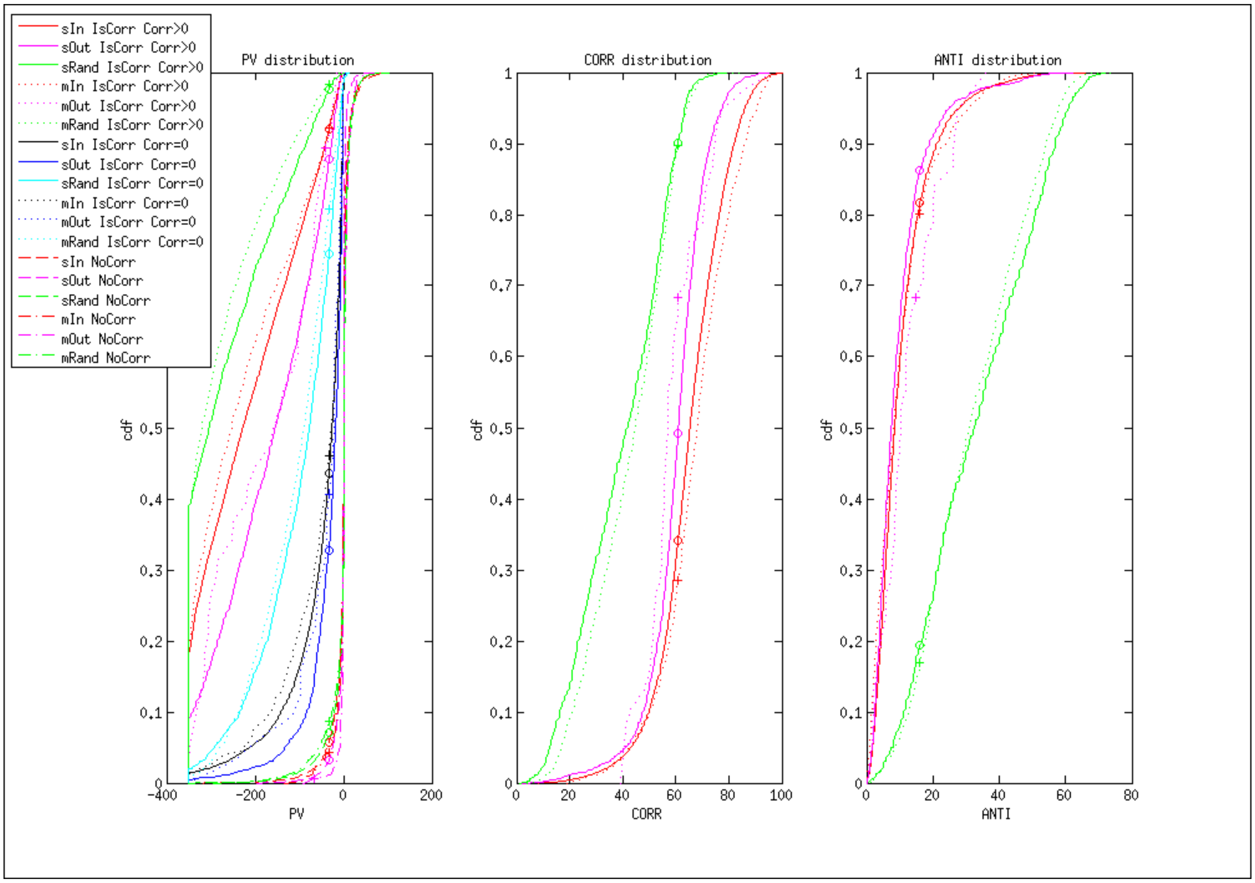


**fig.32 Distributions of CORR, ANTI and PV**. In legend, first letter s or m stands for single or multiple, respectively. NoCorr indicate paired probesets that are never correlated. IsCorr indicates paired probesets that are correlated in at least one network, and in this case statistics concerning networks in which CORR=0 (Corr=0)or CORR>0 (Corr>0) are displayed separately.

### FIG33

Frequency distribution of different categories of paired probesets according to te number of positive networks. In category (target inside exons) and Rand category (random pairs) patterns are symetrical of each other. Out category (target inside introns, or upward and downward 2kb sequences) has an intermediate pattern.

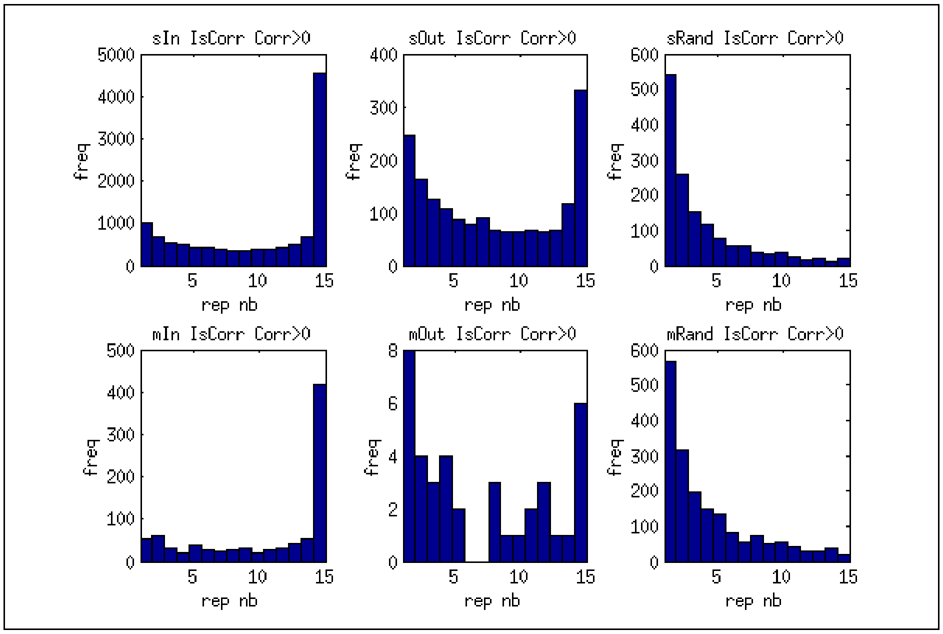


**fig.33 Frequency of positive networks**. Same legend that FIG17 (rep nb = number of positive networks).

### FIG34

Ten paired probesets that are positively correlated (InSim single) in half of the networks are selected randomly. Reproducibility of the CORR or ANTI of neighbour probesets with one of the paired probeset is studied across networks. We observe a great stability of the different values calculated in different networks.

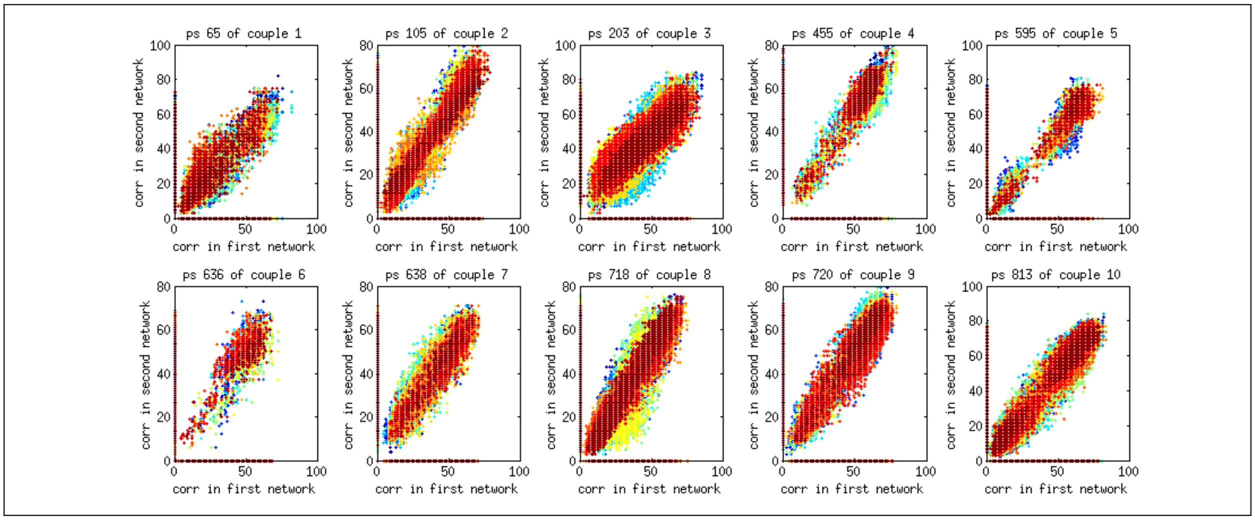


**fig.34a Reproducibility of CORR values between networks(Corr>0)**. Each comparison between two networks is colored differently.

**fig.34b** Reproducibility of CORR values between networks(Corr=0)

**fig.34c** Reproducibility of ANTI values between networks(Corr>0)

**fig.34d** Reproducibility of ANTI values between networks(Corr=0)

Study of the reproducibility of the CORR or ANTI of neighbour probesets with both probesets of a pair inside each network. We observe that reproducibility is good irrespective of the CORR value between the two probesets of the pair. By selecting pair of probesets that are positively correlated in half of the networks, we have a high probability of selecting probesets that target the same transcript(s), hence the reproducibility of their neighbourhood in all networks.

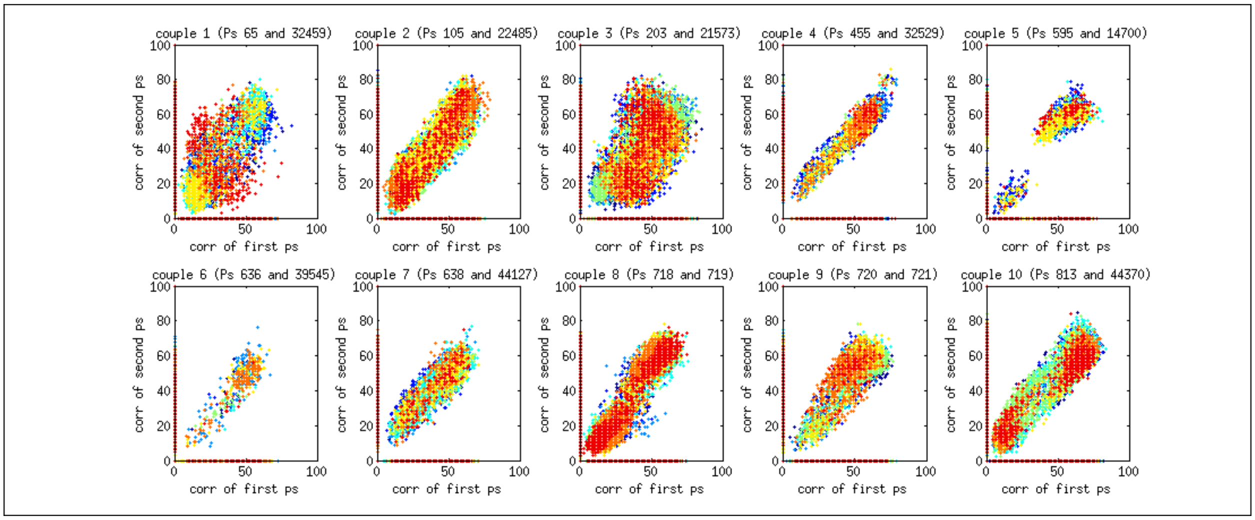


**fig.34e Reproducibility of CORR values inside a nework(Corr>0)**. Each comparison is colored differently.

**fig.34f** Reproducibility of CORR values inside a nework(Corr=0)

**fig.34g** Reproducibility of ANTI values inside a nework(Corr>0)

**fig.34h** Reproducibility of ANTI values inside a nework(Corr=0)

### FIG35

Distribution of some caracteristics of paired probesets (number of common or uncommon genes or transcripts, ...). Three groups of paired probesets are studied (Good: CORR>0 in all the networks, Bad: CORR>0 in at least one network, but mean(CORR), or mean(ANTI) or maen(PV) does not pass the test with corresponding limits, Corr=0: CORR=0 in all the networks).

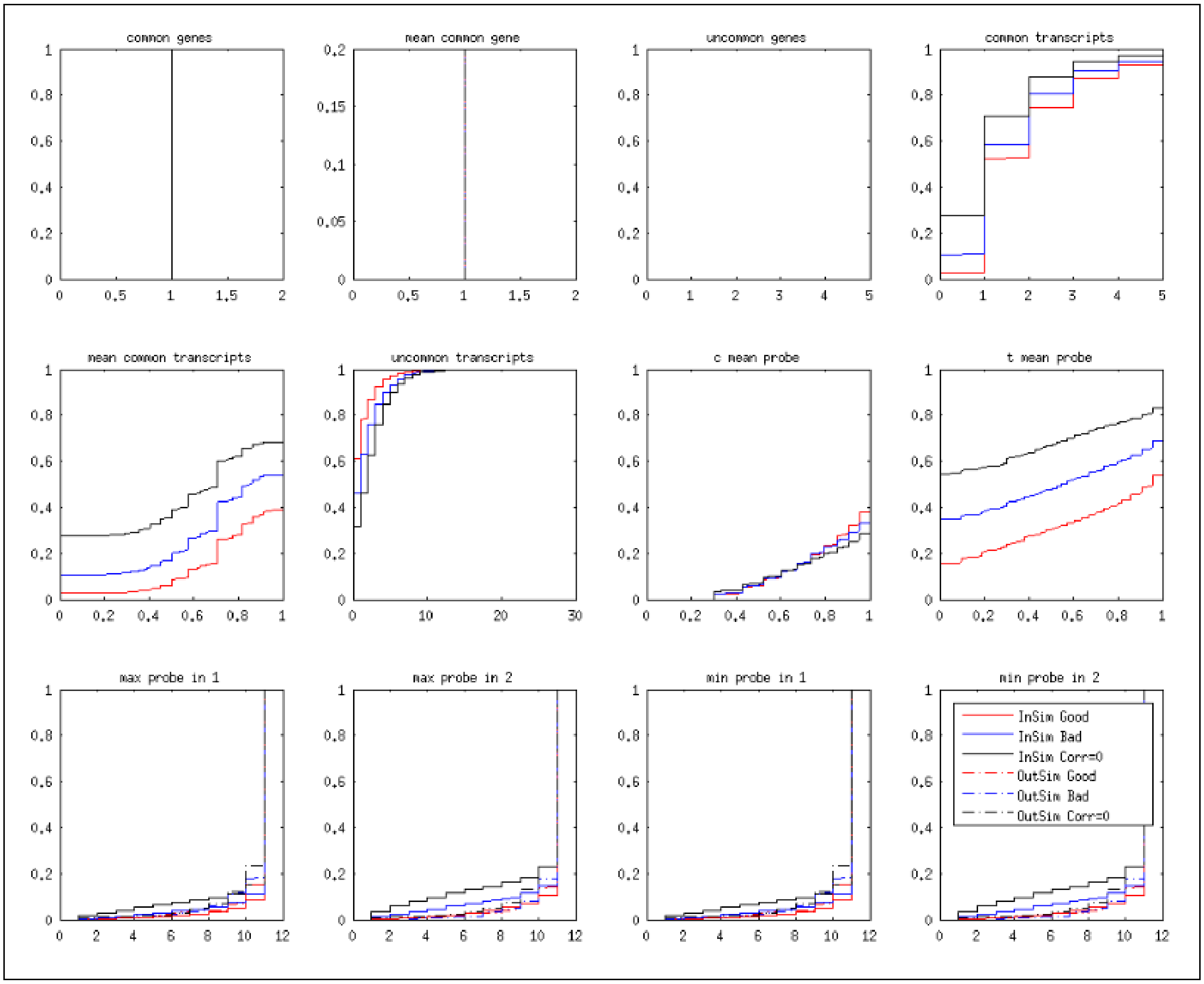


**fig.35a Probe set pair caracteristics (single)**. Mean common items (genes or transcripts) are geometric means (#common/sqrt(#item1*#item2)). 'c mean probe': geometric mean of common probe nb relative to the number of probe targeting common exons in each probeset. 'tMeanGroupProbeIn': geometric mean of common probe nb relative to the number of probe targeting all exons in each probeset.'max(min) probe in 1(2)': greatest (lowest) number of targeting probe for the first (snd) probeset.

**fig.35b** Probe set pair caracteristics (multiple)

### FIG36

CORR, ANTI and PV distribution for paired probesets with CORR>0 in all networks.

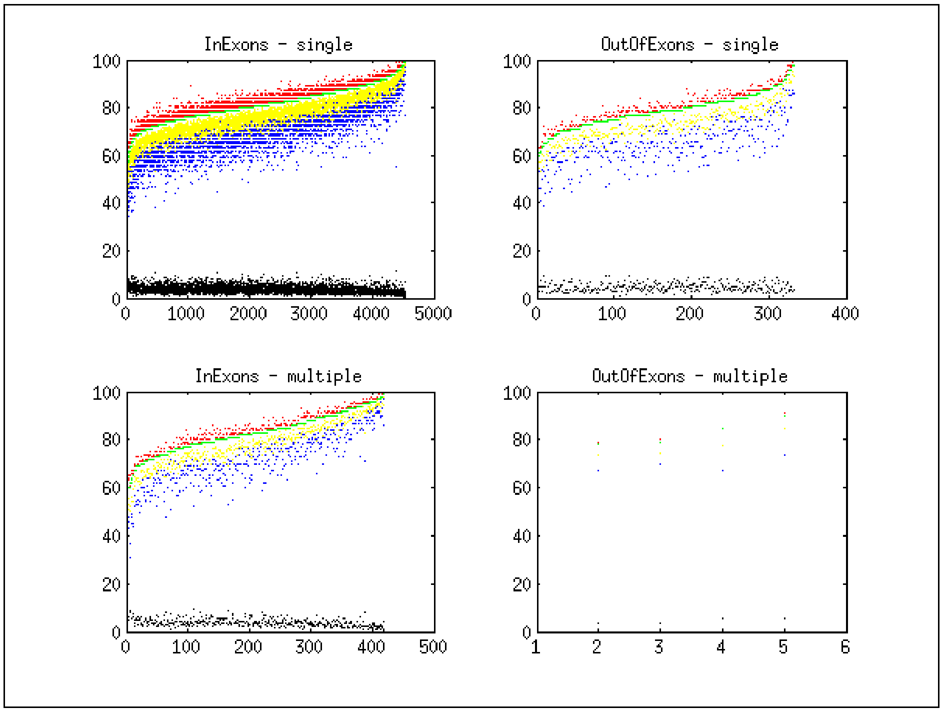


**fig.36a** **CORR distribution**. Blue: minimal values, red: maximal values, yellow: mean values, black= std values, green: 95th percentile. Data are sorted according to the percentile values.

**fig.36b** ANTI distribution green: 5th percentile

**fig.36c** PV distribution green: 5th percentile

### FIG37

Frequency of paired probeset according to the number of positive networks. A network is positive for a given pair of probeset if the pair passes the test (CORR>=CorrLimit and ANTI<=AntiLimit and PV<=PvLimit).

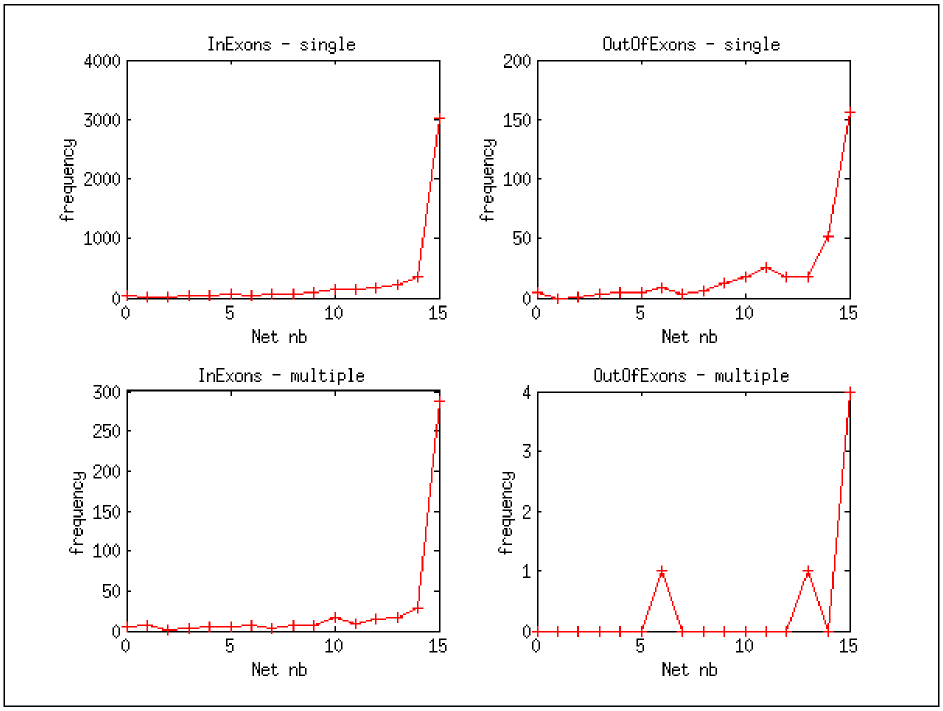


**fig.37 Paired probeset frequency.** 'Net nb': number of positive networks.

### FIG38

Properties of paired probeset that passes or don't pass the similarity test in one network.

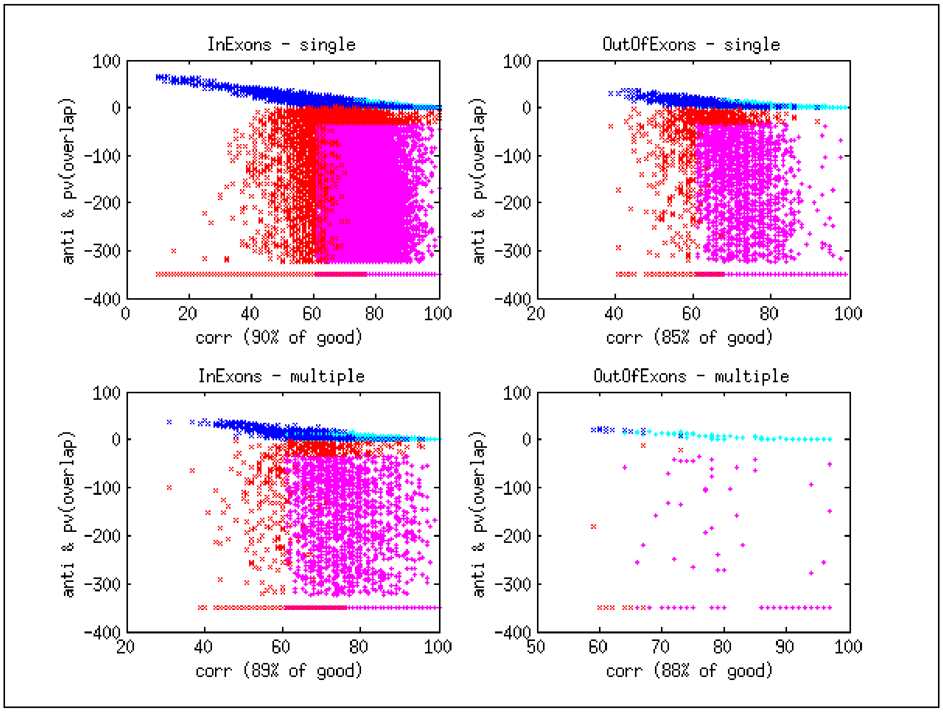


**fig.38 Relation between CORR, ANTI,PV**. Cyan: ANTI for pairs that pass the test, blue: ANTI for pairs that do not pass the test, magenta: PV for pairs that pass the test, red: PV for pairs that do not pass the test.

### FIG39

Comparison of CORR and ANTI measures to Pearson's correlation coefficient, calculated on signals.

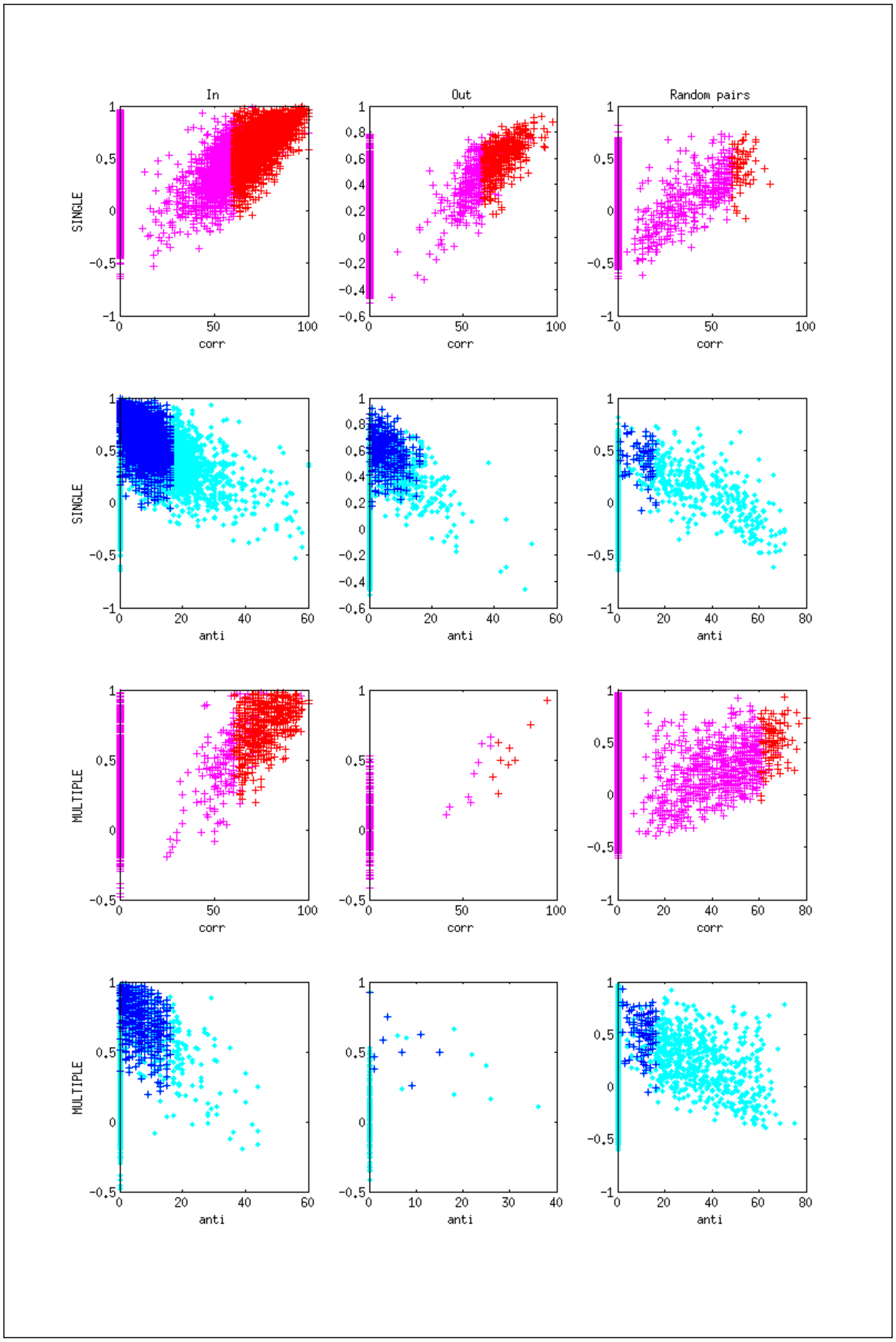


**fig.39 Comparison with Pearson's correlation coefficient** Pearson' correlation is plotted on ordinates. Red and blue crosses indicate respectively CORR and ANTI values for paired probesets that pass the test. Magenta and cyan crosses indicate respectively CORR and ANTI values for paired probesets that do not pass the test.

### merge_ps

```
Species='mouse';
ChipRank=8;
NetRanks=[7:21];
ProbeNbLimit=1;
PvCorrRank=1;
StepRanks=[1:7];
DisplayFlag=1;
SumFlag=0;
merge_ps(Species,ChipRank,NetRanks,ProbeNbLimit,PvCorrRank,StepRanks,DisplayFlag,SumFlag)
```

#### STEP1

**Construct NewPs.**

**FIG40** A higher fraction of probesets are located in AceView genes than in Ensembl genes.

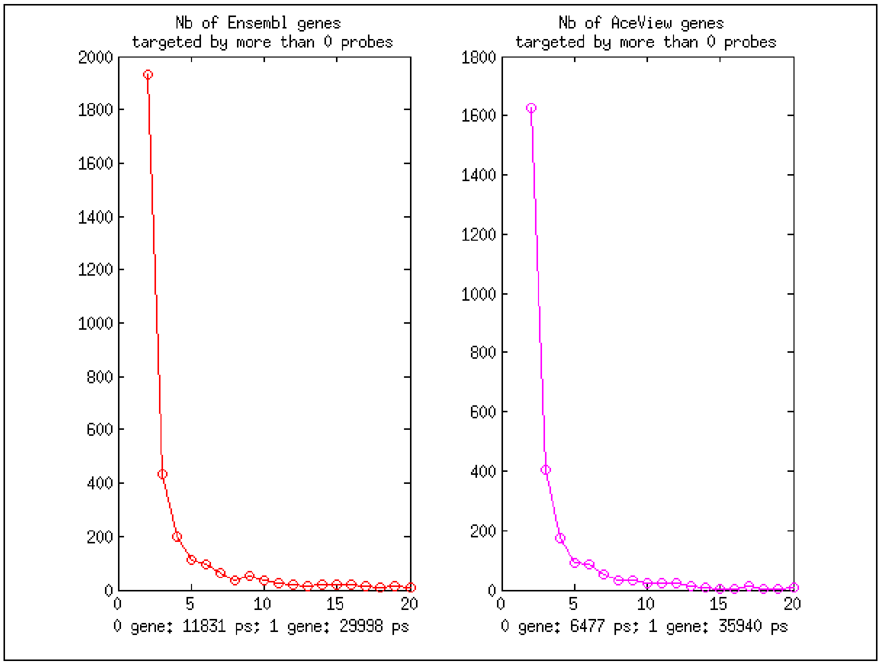


**fig.40a frequency of probesets that target a given number of genes.** Red: Ensembl genes, magenta: AceView genes.

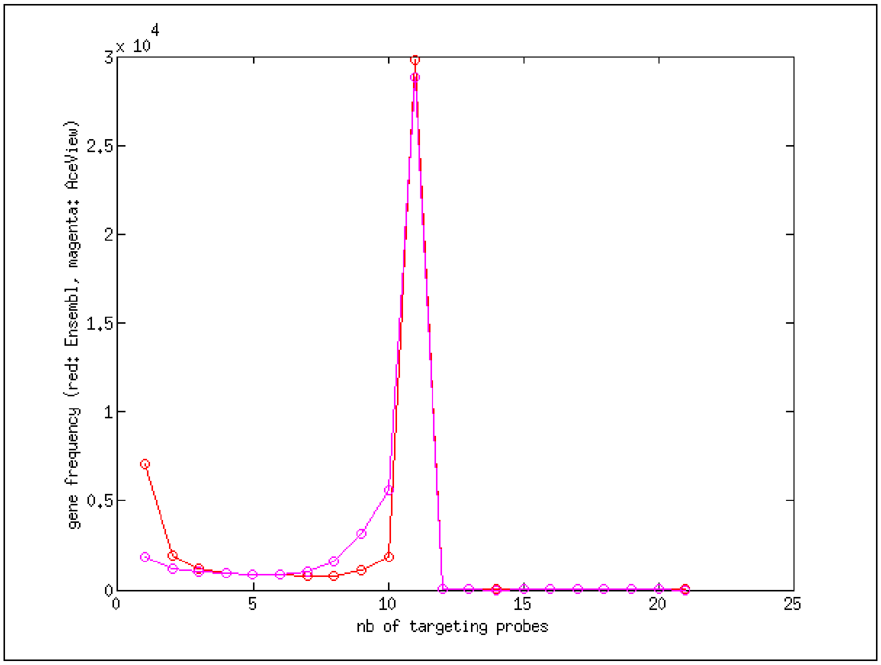


**fig.40b frequency of genes that are targeted by a given number of probes.** Red: Ensembl genes, magenta: AceView genes.

### STEP2

**Calculate limits for CORR, ANTI and PV.**

### STEP3

**Update NewPs.**

### STEP4

**Construct PsBy.**

### STEP5

**Grouping probesets that target the same transcript(s)**. For a given value of TestLimit parameter, paired probesets A and B are considered as targeting the same transcript(s) if they are similar, i.e. if their CORR, ANTI and PV values pass the test (CORR>=CorrLimit and ANTI<=AntiLimit and PV<=PvLimit) in a number of networks equal to or higher than TestLimit. If more than two probesets exist in the currently processed bicluster, there could exist triangle(s), that is series of three probesets such that any pair pass the test. Having completed the list of all triangles, triangles that have a common edge are merged. Doing that, it could occur that one of the pair does not pass the test (bad link: for example if we merge ABC and ABD, if neither BCD nor ACD exist, that means that paired probesets C and D do not pass the test). FIG43 shows that most of these paired probesets linked with ‘bad links’ pass the test in a high number of networks, which explains why we keep them inside the group. However if could occur that two groups have only one probeset in common. In this case this particular probeset is considered as a hub (or a pivot), and is kept apart in a special list indicating its relationships with oher groups.

**FIG41** Statistics on the third edge in probeset triangles. If probeset A and B form a pair that pass the test in **at least** a given number of networks (TestLimit=15 - delta), and if probeset A and C form a pair that pass also the test in the same conditions, what is the frequency distribution of the pair (B,C) (third edge of the triangle) ?

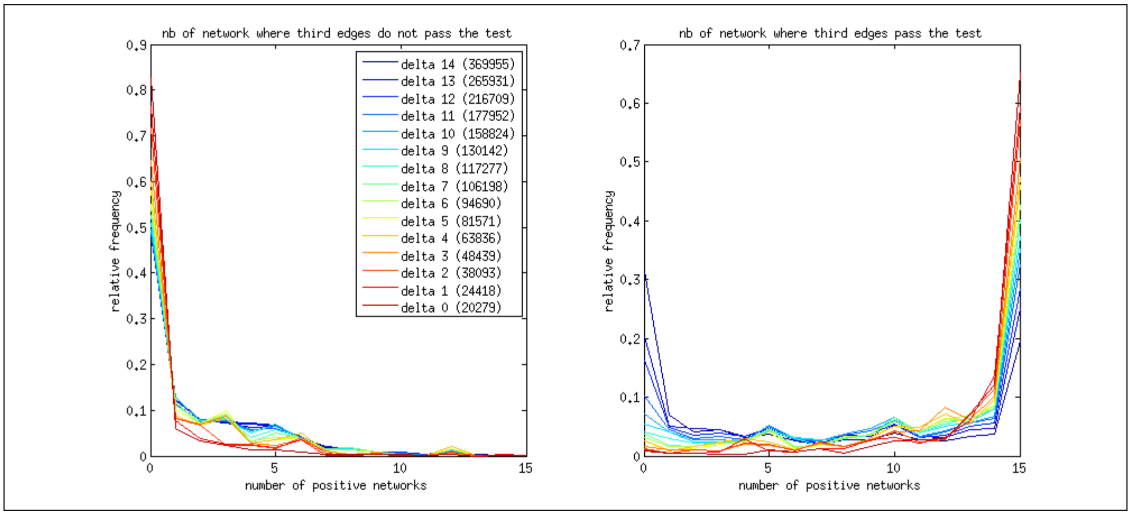


**fig.41 frequency of third edges that pass (right panel) or do not pass (left panel) the test in a given number of networks** The statistics concern third edges that belong to a triangle where the two other edges pass the test in at least a given number (15 - delta) of networks.

**FIG42** Statistics on the third edge in probeset triangles. If probeset A and B form a pair that pass the test **exactly** in a given number of networks (TestLimit=15 - delta), and if probeset A and C form a pair that pass also the test in the same conditions, what is the frequency distribution of the pair (B,C) (third edge of the triangle) ?

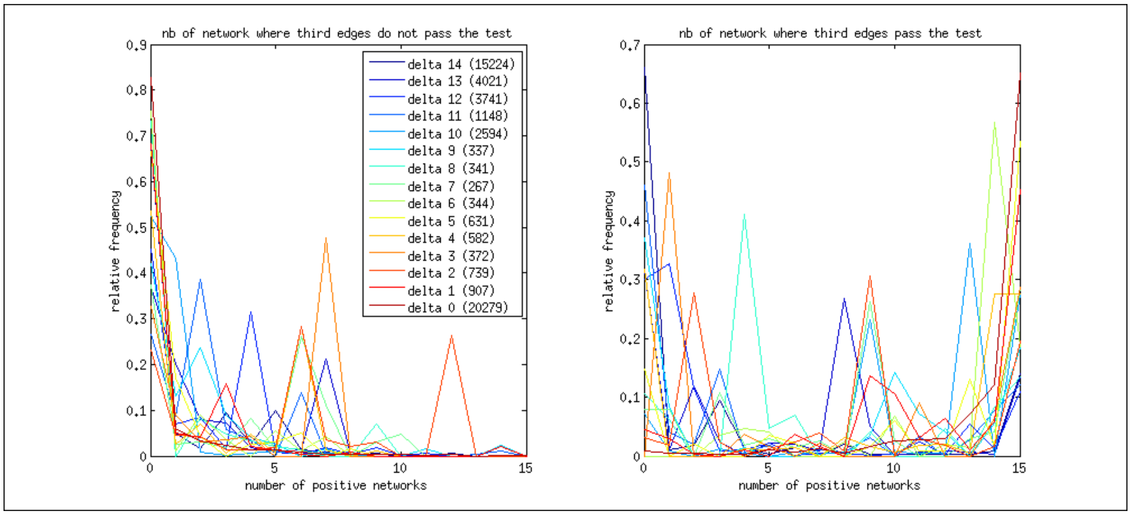


**fig.42 frequency of third edges that pass (right panel) or do not pass (left panel) the test in a given number of networks** The statistics concern third edges that belong to a triangle where the two other edges pass the test in a given number (15 - delta) of networks.

**FIG43** Statistics on grouped probesets

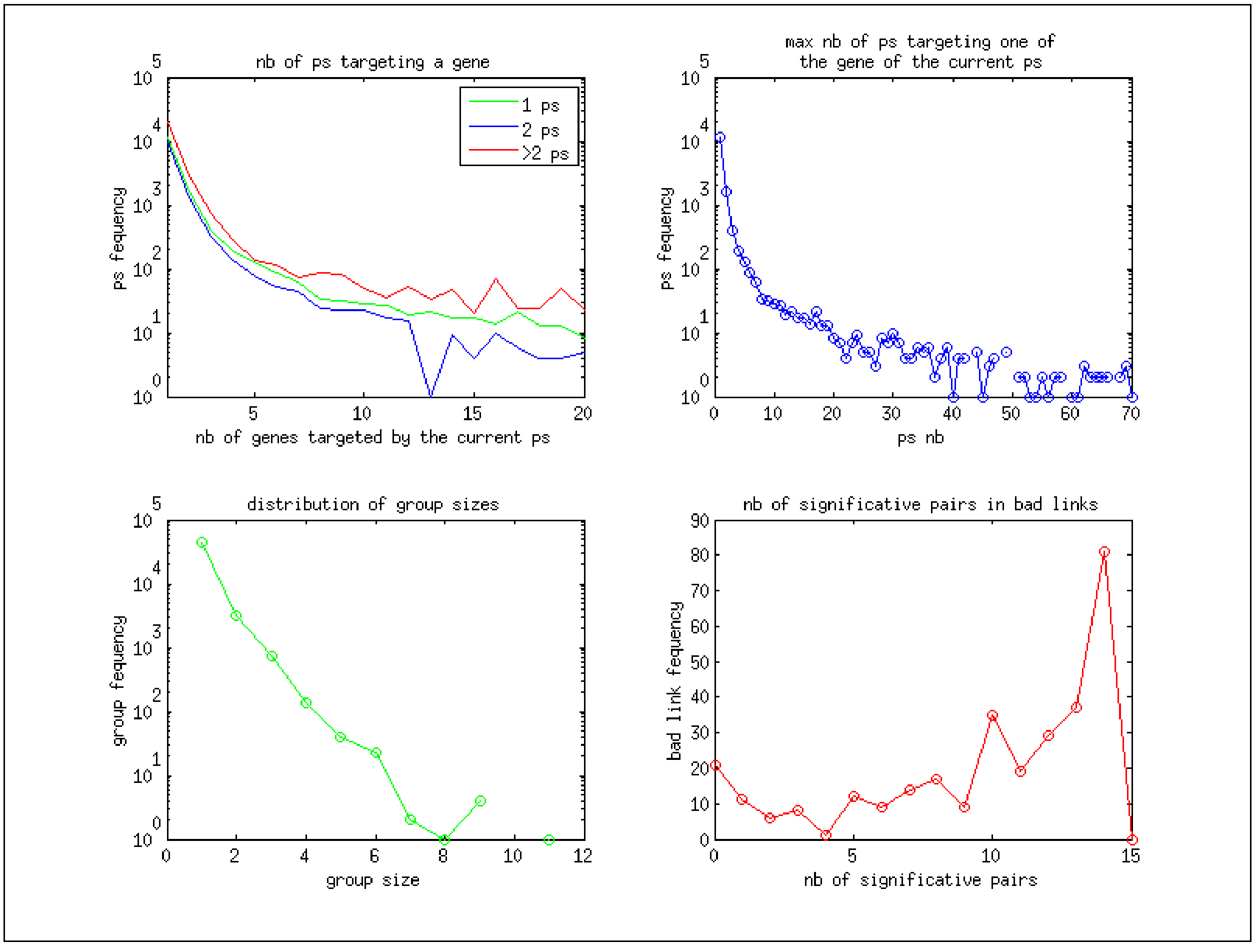


**fig.43_d0 Statistics on grouped probesets at delta=0 (TestLimit=15)**

Left upper panel: Number of genes targeted by the current probeset.

Green, blue and red curves correspond repectively to probesets for which

- all targeted genes are targeted only by the current probeset
- some genes are targeted by the current probeset plus a single other one
- some genes are targeted by the current probeset plus two or more another probesets

Right upper panel: maximal number of probeset targeting one of the genes targeted by the current probeset.

Left lower panel: distribution of size of probeset groups.

Right lower panel: number of positive networks for pairs of probesets which have been included in a group following merging of triangles, but which do not pass the test in 15 networks.

**fig.43_d4** Statistics on grouped probesets at delta=4 (TestLimit=11)

**fig.43_d8** Statistics on grouped probesets at delta=8 (TestLimit=7)

**fig.43_d11** Statistics on grouped probesets at delta=11 (TestLimit=4)

**fig.43_d14** Statistics on grouped probesets at delta=14 (TestLimit=1)

**FIG44**

**For each probeset (the current probeset), we search genes that are targeted (by the current probeset) either with the highest** number of probes (i.e. the number of probes targeting the assigned gene), or with a lesser number of probes. For each of these categories, we recover in different lists, the paired probesets that target the same transcript(s). The two upper plots refer to the second list (paired probesets corresponding to the genes targeted by the current probeset with a smaller number of probes), and the two lower plots to the first list. By plotting, for each pair of probeset, the maximal number of probes targeting the gene, and the difference between the two number of probes, we have direct grasp of the difference of distribution between the two categories.

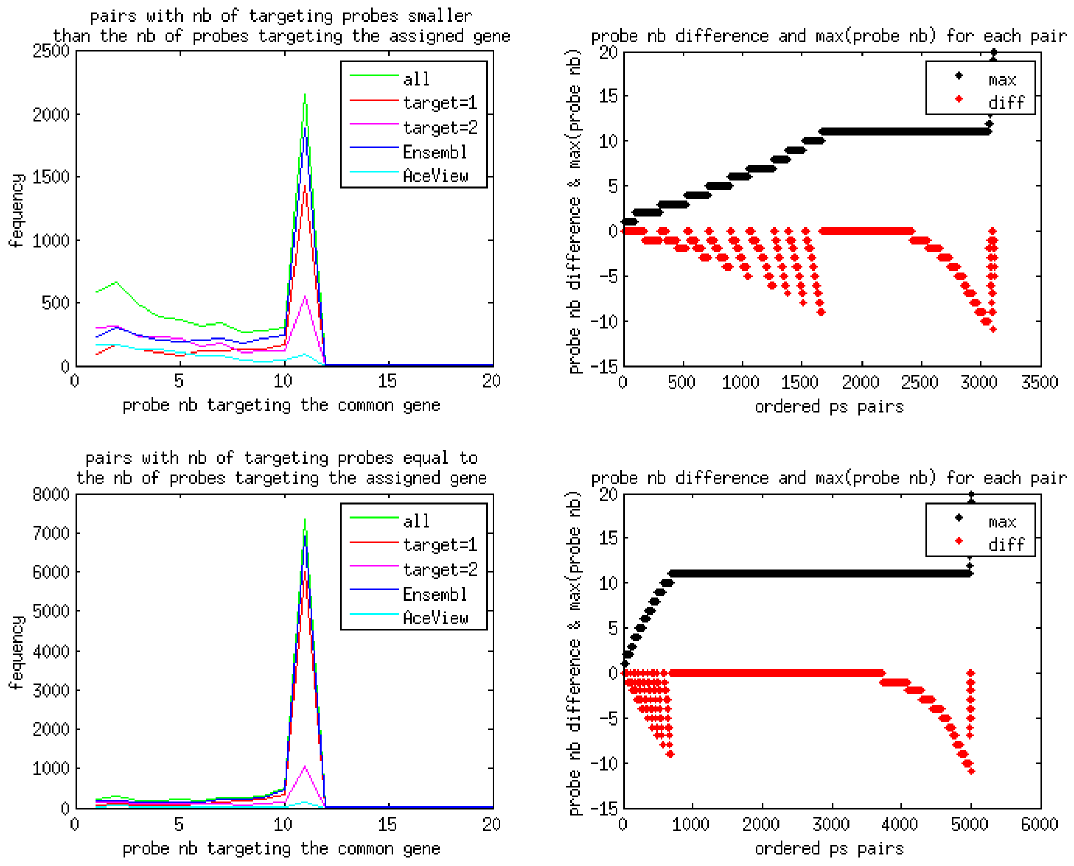


**fig.29_d0 Statistics on pairs of probesets at delta=0**

Left panels: Frequency of probesets according to the number of probes targeting a gene.

all : all probesets

target = 1 : probesets inside exons

target = 2 : probesets outside exons

ENS: probeset targeting Ensembl genes

AceView: probeset targeting AceView genes

Right panels: For each pair of probeset is plotted the maximal number of probes targeting the gene, and the difference between the two number of probes.

**fig.44_d4** Statistics on pairs of probesets at delta=4

**fig.44_d8** Statistics on pairs of probesets at delta=8

**fig.44_d11** Statistics on pairs of probesets at delta=11

**fig.44_d14** Statistics on pairs of probesets at delta=14

### FIG45

**Distribution of number of probes in class MM. For each probeset (the current probeset), we consider independantly** the gene that is assigned to the current probeset, and all the other gene(s) targeted by the current probeset but not assigned to it. Then we consider for each gene, all the possible pairs targeting this gene, and display the distribution of the number of targeting probes.

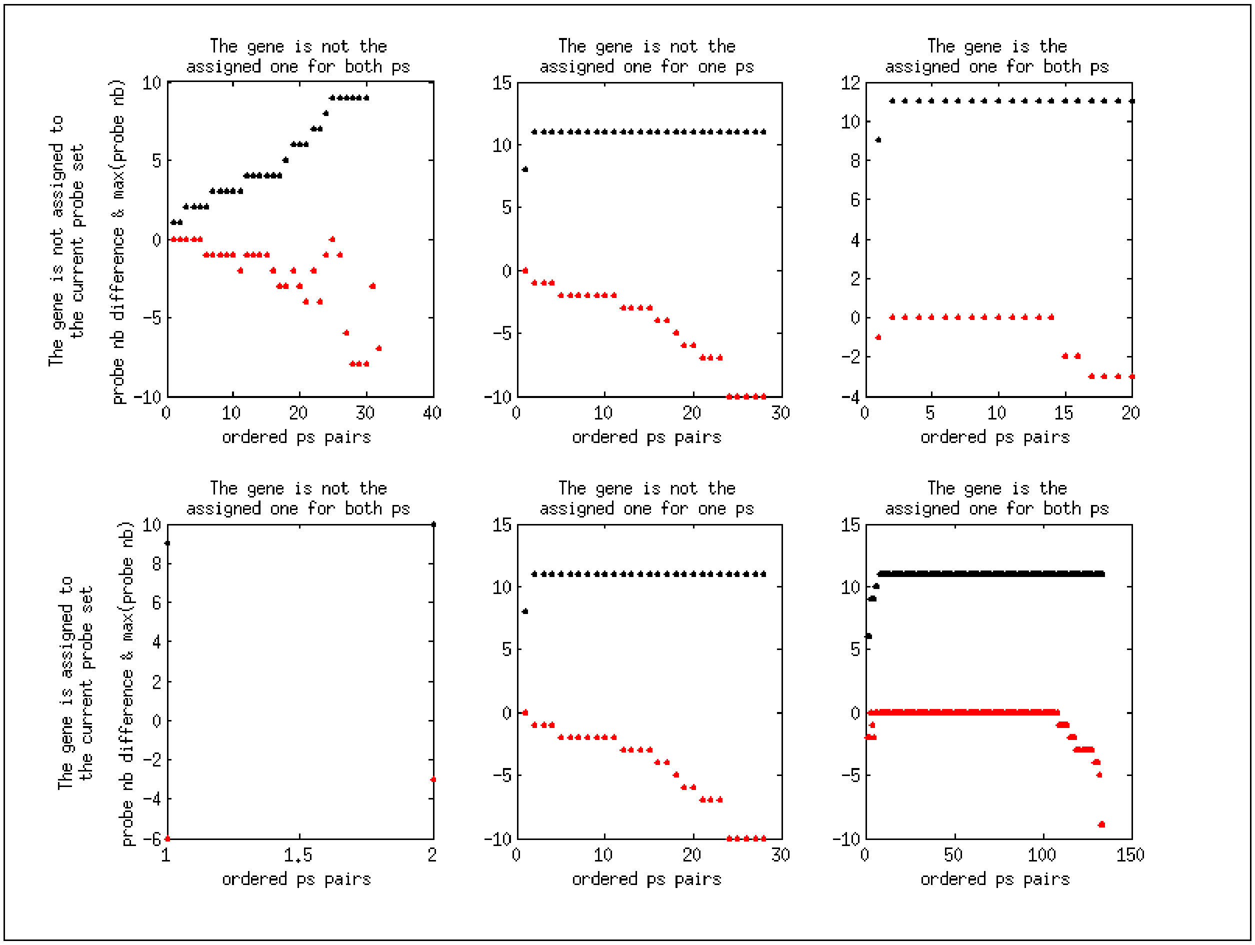


**fig.45_d0 Probe nb difference distribution according to gene assignation in MM class at delta=0**

**fig.45_d4** Probe nb difference distribution according to gene assignation in MM class at delta=4

**fig.45_d8** Probe nb difference distribution according to gene assignation in MM class at delta=8

**fig.45_d11** Probe nb difference distribution according to gene assignation in MM class at delta=11

**fig.45_d14** Probe nb difference distribution according to gene assignation in MM class at delta=14

**FIG46 Distribution of number of probes in class CX.**

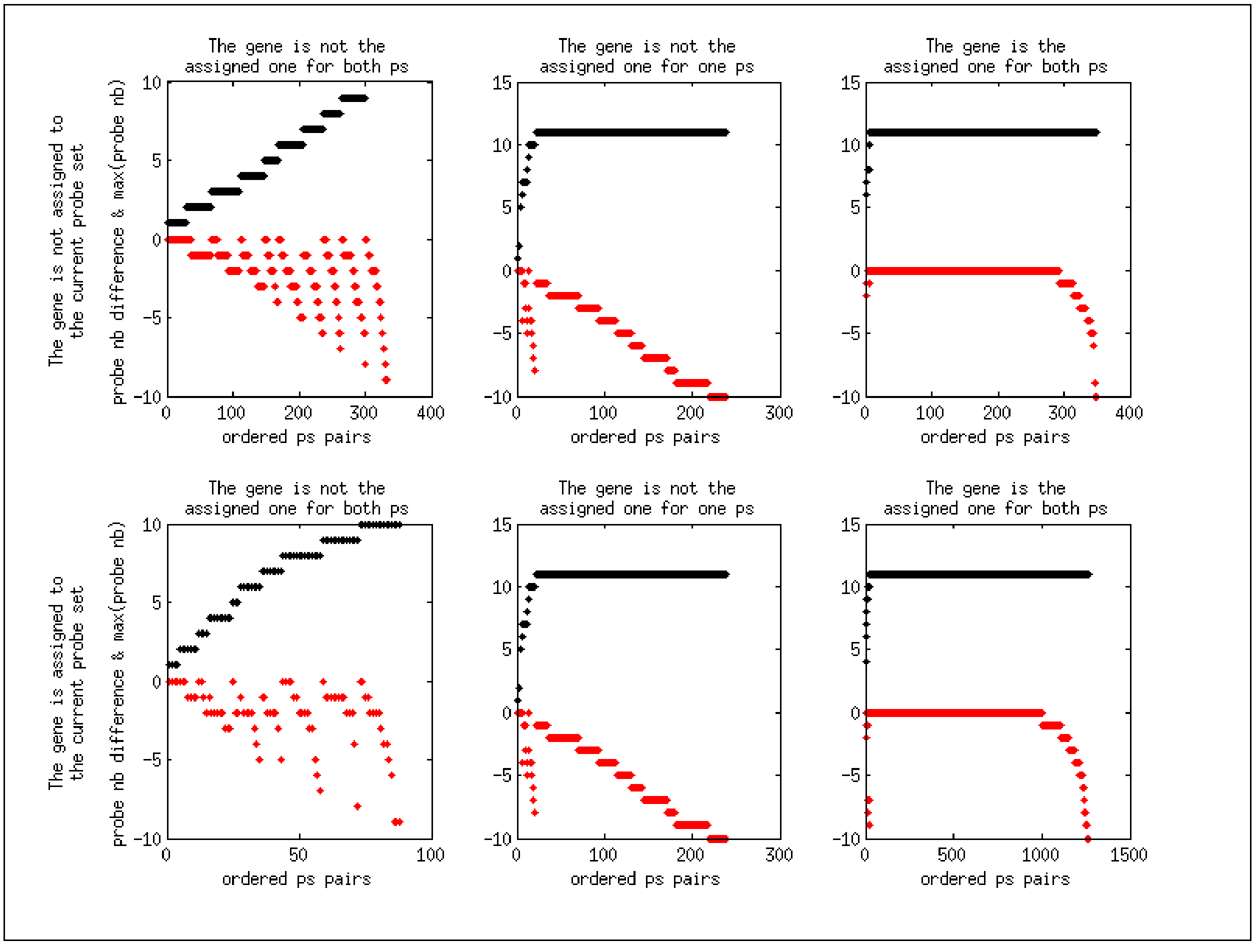


**fig.46_d0 Probe nb difference distribution according to gene assignation in CX class at delta=0**

**fig.46_d4** Probe nb difference distribution according to gene assignation in CX class at delta=4

**fig.46_d8** Probe nb difference distribution according to gene assignation in CX class at delta=8

**fig.46_d11** Probe nb difference distribution according to gene assignation in CX class at delta=11

**fig.46_d14** Probe nb difference distribution according to gene assignation in CX class at delta=14

**FIG47 Distribution of number of probes in class MS.**

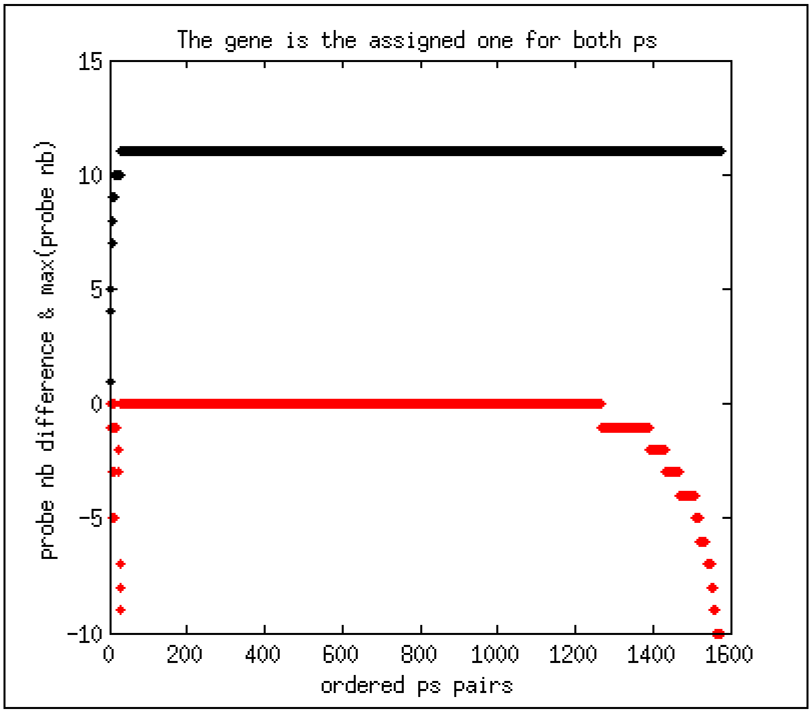


**fig.47_d0 Probe nb difference distribution according to gene assignation in MS class at delta=0**

**fig.47_d4** Probe nb difference distribution according to gene assignation in MS class at delta=4

**fig.47_d8** Probe nb difference distribution according to gene assignation in MS class at delta=8

**fig.47_d11** Probe nb difference distribution according to gene assignation in MS class at delta=11

**fig.47_d14** Probe nb difference distribution according to gene assignation in MS class at delta=14

**FIG48 Probe nb difference according to class.**

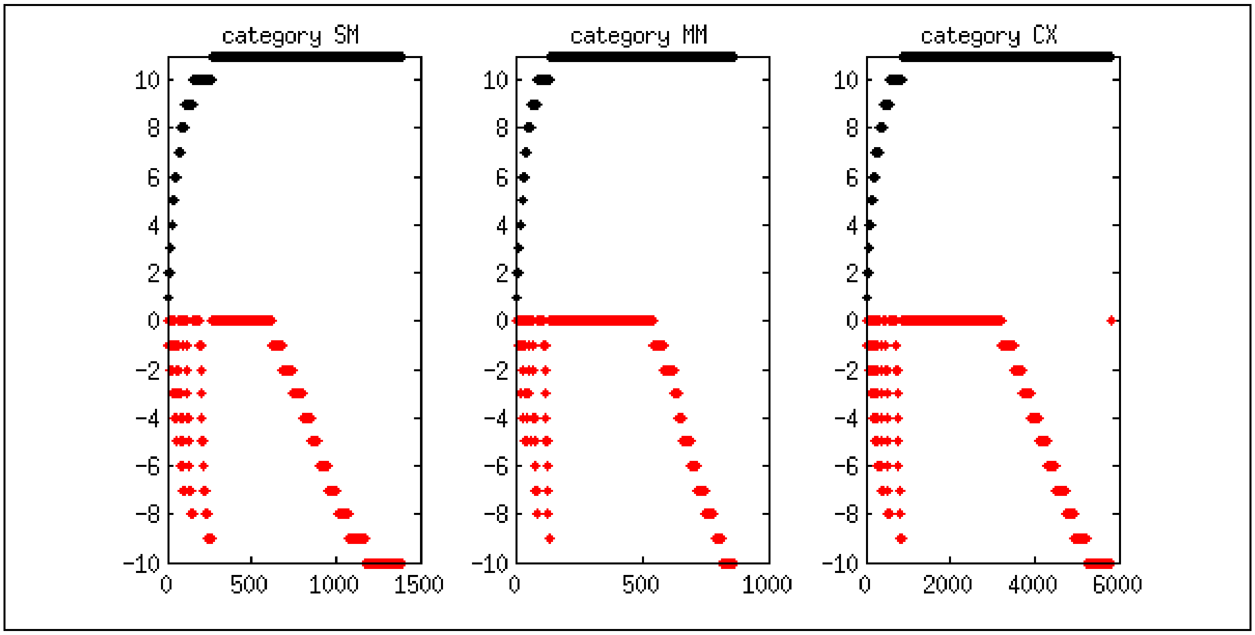


**fig.48_d0 Probe nb difference according to class at delta=0**

**fig.48_d4** Probe nb difference according to class at delta=4

**fig.48_d8** Probe nb difference according to class at delta=8

**fig.48_d11** Probe nb difference according to class at delta=11

**fig.48_d14** Probe nb difference according to class at delta=14

**FIG49 Probe nb difference according to target type.**

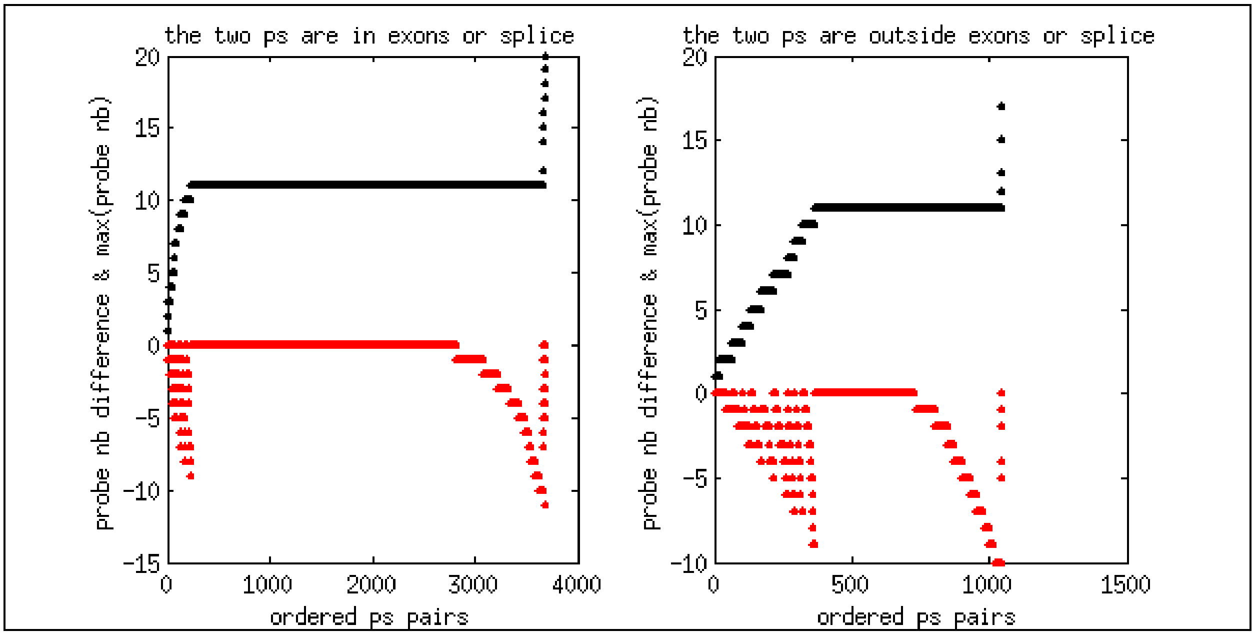


**fig.49_d0 Probe nb difference according to target type at delta=0**

**fig.49_d4** Probe nb difference according to target type at delta=4

**fig.49_d8** Probe nb difference according to target type at delta=8

**fig.49_d11** Probe nb difference according to target type at delta=11

**fig.49_d14** Probe nb difference according to target type at delta=14

**FIG50 Probe nb difference according to gene assignation and target type (1 = inside exons, 2 = outside exons).**

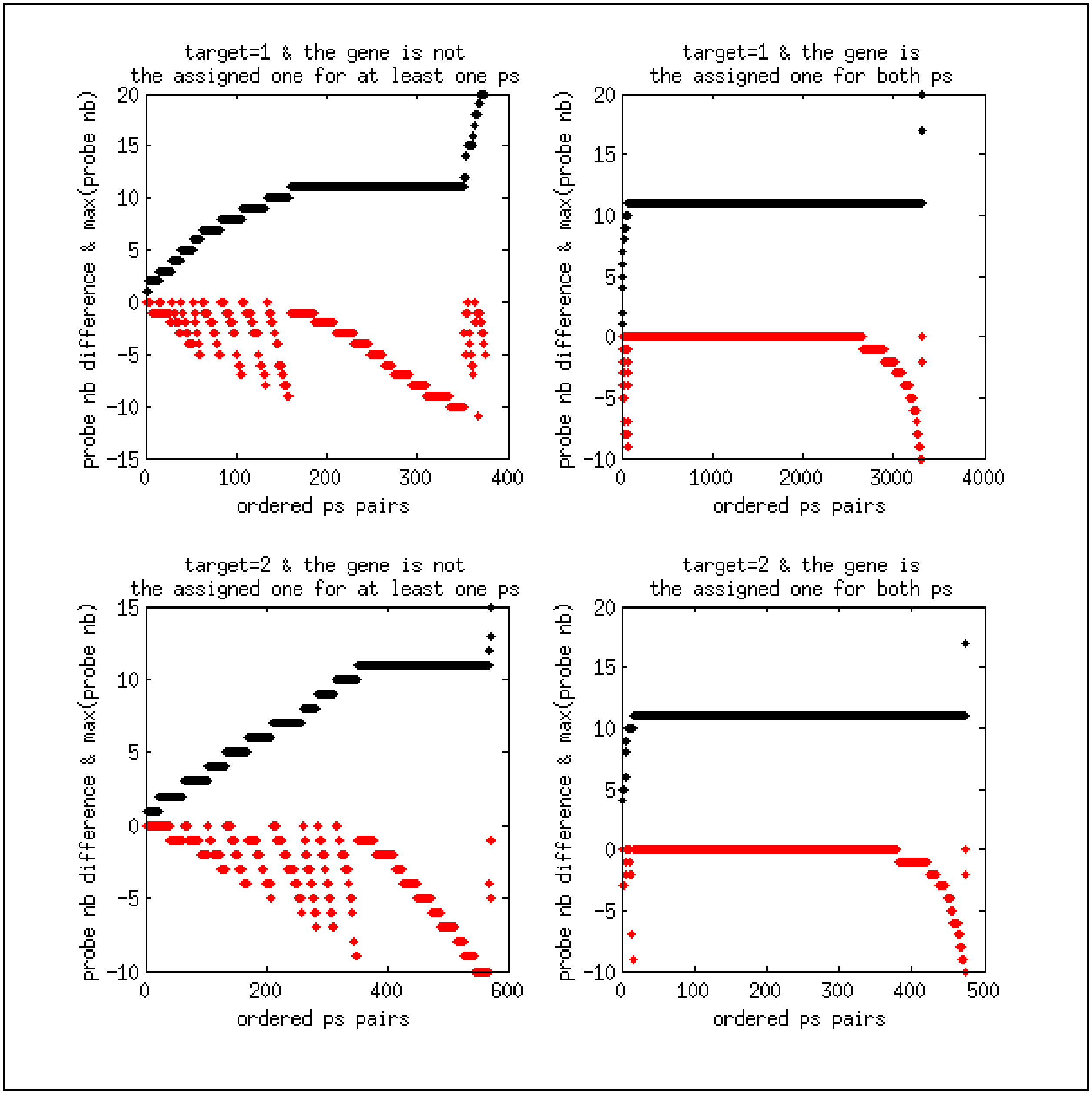


**fig.50_d0 Probe nb difference according to gene assignation and target type at delta=0**

**fig.50_d4** Probe nb difference according to gene assignation and target type at delta=4

**fig.50_d8** Probe nb difference according to gene assignation and target type at delta=8

**fig.50_d11** Probe nb difference according to gene assignation and target type at delta=11

**fig.50_d14** Probe nb difference according to gene assignation and target type at delta=14

### STEP6

#### Construct PsMatrix

### STEP7

#### FIG51 to 53

##### FIG51 Pearson correlation coefficient distribution according the level of reproducibility of similarity between paired probesets.

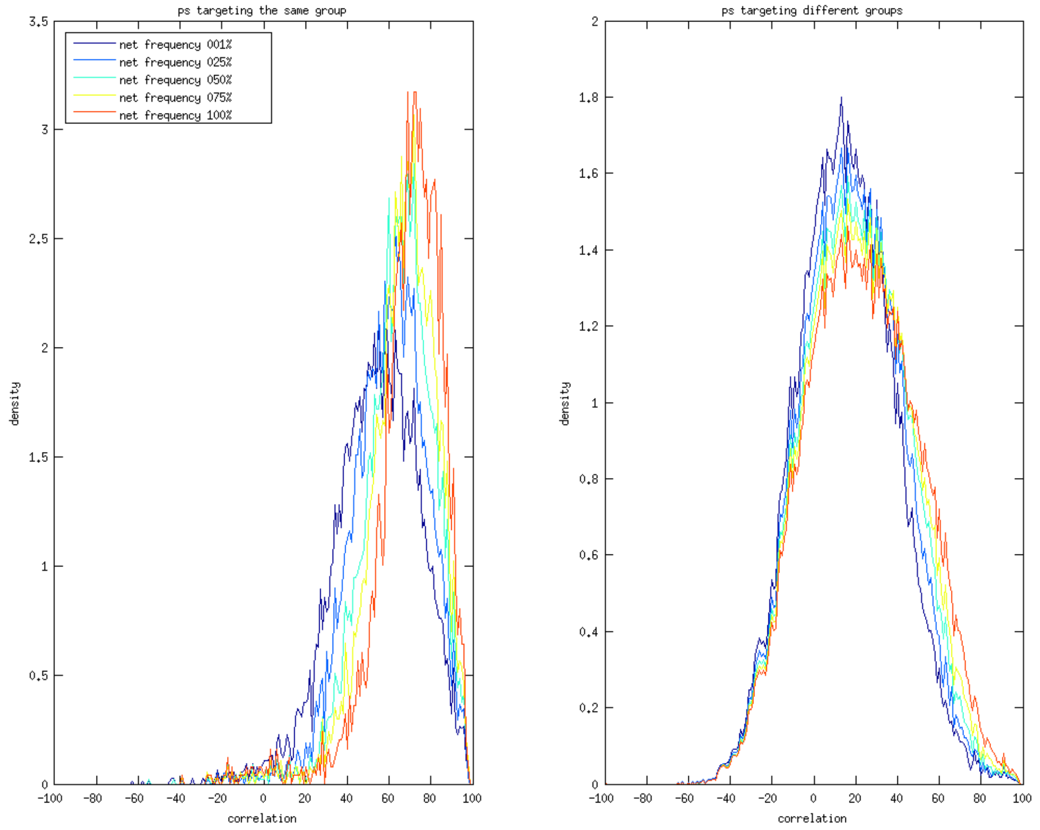


**fig.51 Pearson correlation coefficient distribution.**

Left panel: Distribution among similar paired probesets at several level or reproductibility. The distributions corresponding to low level or reproducibility are shifted towards he left (smaller correlation coefficients).

Right panel: Distribution among dissimilar paired probesets. Distributions are identical.

**FIG52 Frequency of groups of different size according to the level of reproductibility in each class.**

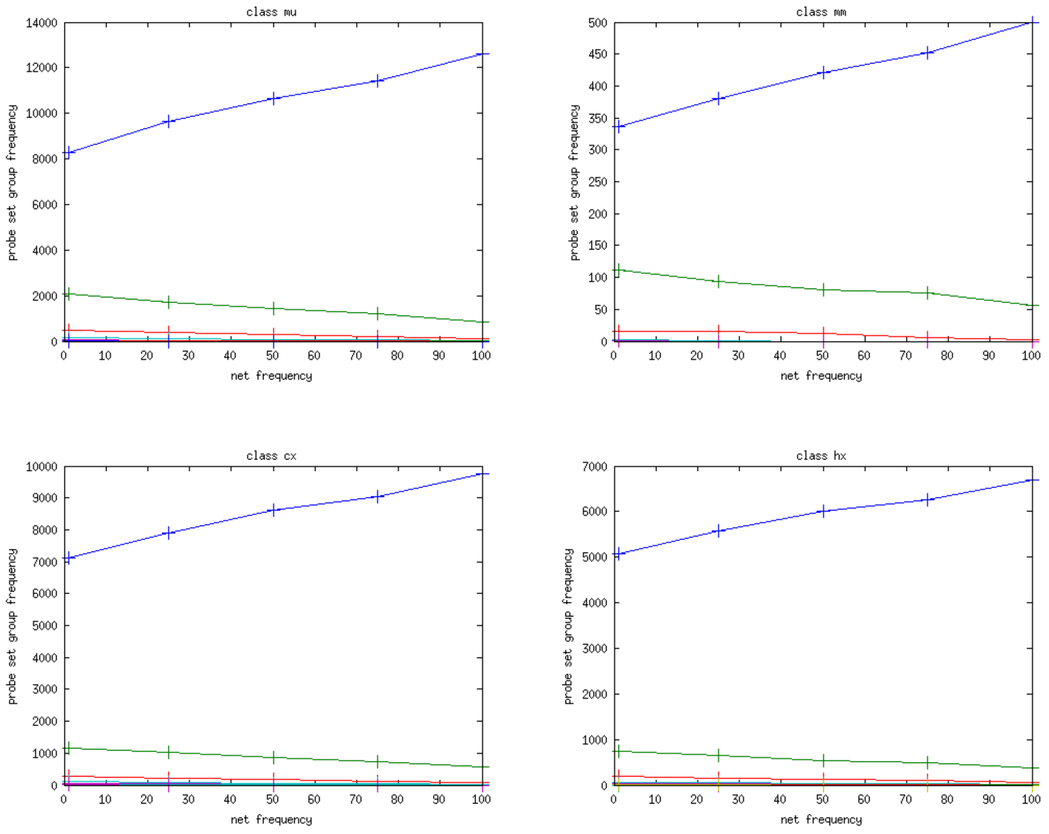


**fig.52 Frequency of groups.** Blue, green and red lines refers to group of size 1,2 and 3 respectively.

**FIG53 Frequency of groups of different size according to the level of reproductibility.**

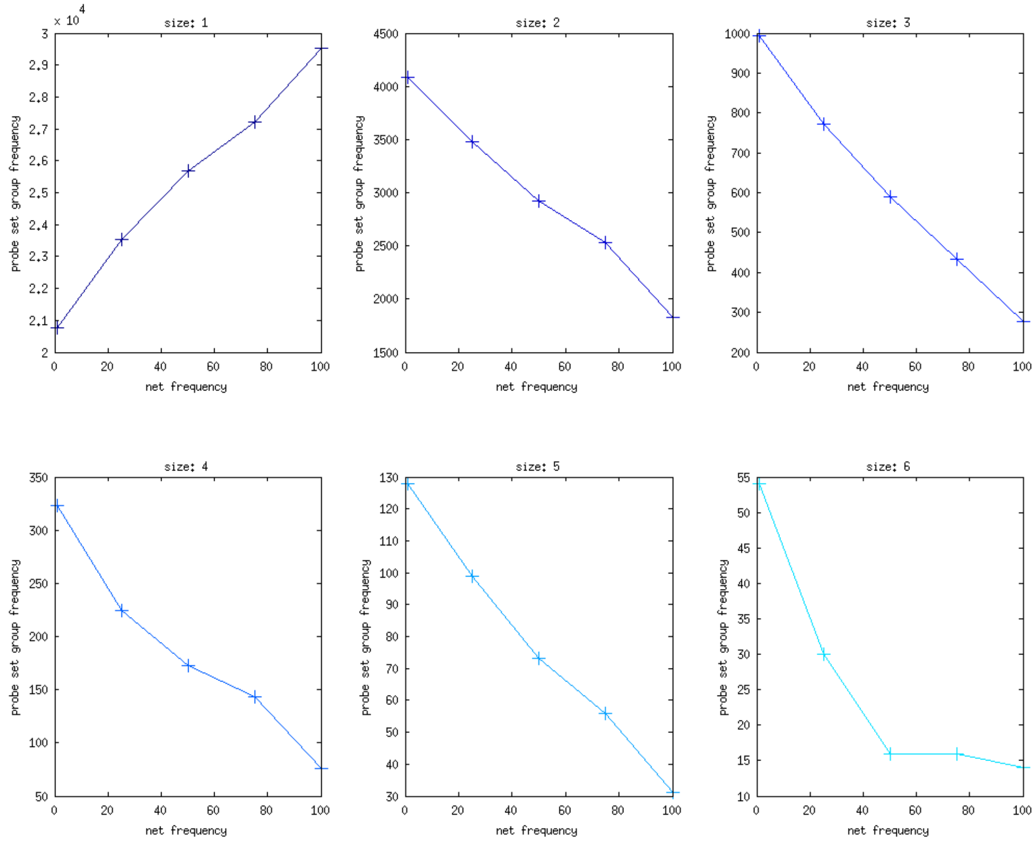


**fig.53 Frequency of groups according to their size.** Classes MS, MM, CX and HX are joined.

### STEP8

**Write results.**

**FIG54** For each chip model, two results are available, corresponding to probeset targeting genes with at least one (e.g. m8_probenb1) or seven (e.g. m8_probenb7) probes. For example, the m8 chip results archived in m8_probenb1 contain the following files:

```
1: first probeset ID
2: gene ID assigned to the probesets (either Ensembl ID or eventually AceView or GOP id)
3: gene name
4: rank of the assigned gene to the current probeset
5: position of the assigned gene in NewPs.geneNames
6: target type of assigned gene (1 for probeset in exon, 0 otherwise)
7: source type of assigned gene (1 for Ensembl, 2 for AceView, 3 for GOP)
8: nb of probe targeting the assigned gene
```

```
 9: nb of not assigned genes targeted with the same nb of probes
10: nb of not assigned genes targeted with less nb of probes
11: nb of groups of transcripts corresponding to the assigned gene
12: rank of the parent probeset
13: Rank of the group of transcripts targeted by the current
    probeset in the assigned gene
14: Rank of the group(s) of transcripts targeted by the current
    probeset, if it is a pivot
15: 0,1 indicates if the current probeset is a pivot
16: 0,1 indicates if the current probeset is paired with a pivot
17: nb of probesets that do not target the assigned gene but target
    a common gene with the current probeset
18: nb of genes that are targeted by probesets that do not target the
    assigned gene
19: nb of genes that are targeted by other probeset with a nb
    of probes higher than the number of probes of the current probeset that
    target the assigned gene
20: ClassRank
21: and beyond: nb of  other genes targeted with all possible nb of probes (from 1 to ProbeNb)
```

**fig.54 Files m8_n12_netnb21_probenb1_netprc{001,025,050,075,100}_pvcorr1_psmatrix.txt** contains PsMatrix corresponding to pair of probesets assumed to targeting the same transcripts(s) in at least 1, 25,50,75 and 100% of networks.

**FIG55 File m8_n12_netnb21_probenb1_pvcorr1_pspair.txt** contains all detected probeset pairs. Overlaping exons are merged to build up a group of exons.

```
 1: gene ID assigned to the two probesets forming a pair (either Ensembl ID or eventually AceView or
 2: gene name
 3: first probeset ID
 4: second probeset ID
 5: first probeset rank in PsMatrix
 6: second probeset rank in PsMatrix
 7: indicates if the probesets are similar in 1% PsMatrix
 8: indicates if the probesets are similar in 25% PsMatrix
 9: indicates if the probesets are similar in 50% PsMatrix
10: indicates if the probesets are similar in 75% PsMatrix
11: indicates if the probesets are similar in 100% PsMatrix
     pivot information for fields 7 to 10, if the two probesets are similar:
     if first and second probesets are not a pivot => 1
     if only one of them is a pivot => 2
     if both are pivots => 3
12: probeset class (1=SS, 2=SM, 3=MS, 4=MM, 5=CX, 6=HX, 7= no genomic target)
13: percentage of significative comparisons in which the two probesets are positively correlated at F
14: percentage of significative comparisons in which the two probesets are negatively correlated at F
15: percentage of all comparisons in which the two probesets are positively correlated at FDR 10%
16: percentage of all comparisons in which the two probesets are negatively correlated at FDR 10%
17: percentage of all comparisons in which the two probesets are not changed at FDR 10%
18: percentage of significative comparisons in which the two probesets are positively correlated at F
19: percentage of significative comparisons in which the two probesets are negatively correlated at F
20: percentage of all comparisons in which the two probesets are positively correlated at FDR 1%
21: percentage of all comparisons in which the two probesets are negatively correlated at FDR 1%
22: percentage of all comparisons in which the two probesets are not changed at FDR 1%
23: number of probes of the first probeset targeting the assigned gene
```

```
24: number of genes targeted by the first probeset with the same number of probes
25: number of genes targeted by the first probeset with an inferior number of probes
26: number of probes of the second probeset targeting the assigned gene
27: number of genes targeted by the second probeset with the same number of probes
28: number of genes targeted by the second probeset with an inferior number of probes
29: class rank of first probe set
30: class rank of snd probe set
31: v: the probeset pair is tested in all the networks,
    *: the probeset pair is absent of at leat one networks (~1% of all pairs)
32: total number of transcripts targeted by the two probesets
33: number of transcripts targeted in common by the two probesets
34: number of transcripts specifically targeted by the first probeset
35: number of transcripts specifically targeted by the second probeset
36: total number of exons targeted by the two probesets
37: number of probes located in exons targeted in common by the two probesets
38: number of probes located in exons specifically targeted by the first probeset
39: number of probes located in exons specifically targeted by the second probeset
40: total number of groups of exons targeted by the two probesets
41: number of probes located in groups of exons targeted in common by the two probesets
42: number of probes located in groups of exons specifically targeted by the first probeset
43: number of probes located in groups of exons specifically targeted by the second probeset
44: overlaping score for transcripts (column 23*100/column 22)
45: overlaping score for exons
for each targeted exon a local weighted overlap score, using the number of probes of the
first (PNb1) and the second (PNb2) probeset targeting this exon, and the total nb
of probes targeting an exon (PNb)
(PNb1+PNb2)/PNb)*(min(PNb1,PNb2)/max(PNb1,PNb2)
The overlaping score is the mean of all local scores
46: overlaping score for groups of exons
same method used for the overlaping score for exons
47: percentage of first probeset probes located in the last exon
48: percentage of second probeset probes located in the last exon
49: indicates (1/0) if all probes of the first probeset are located in a single exon
50: indicates (1/0) if all probes of the second probeset are located in a single exon
51: percentage of first probeset probes located in the last group
52: percentage of second probeset probes located in the last group
53: indicates (1/0) if all probes of the first probeset are located in a single group
54: indicates (1/0) if all probes of the second probeset are located in a single group
55 to 77: iAceView information, idem to 32 to 54
```

**fig.55 Columns of file describing all detected probeset pairs.**

# FIG56 Relationships between similarity and localisation of probes in common exons or transcripts.

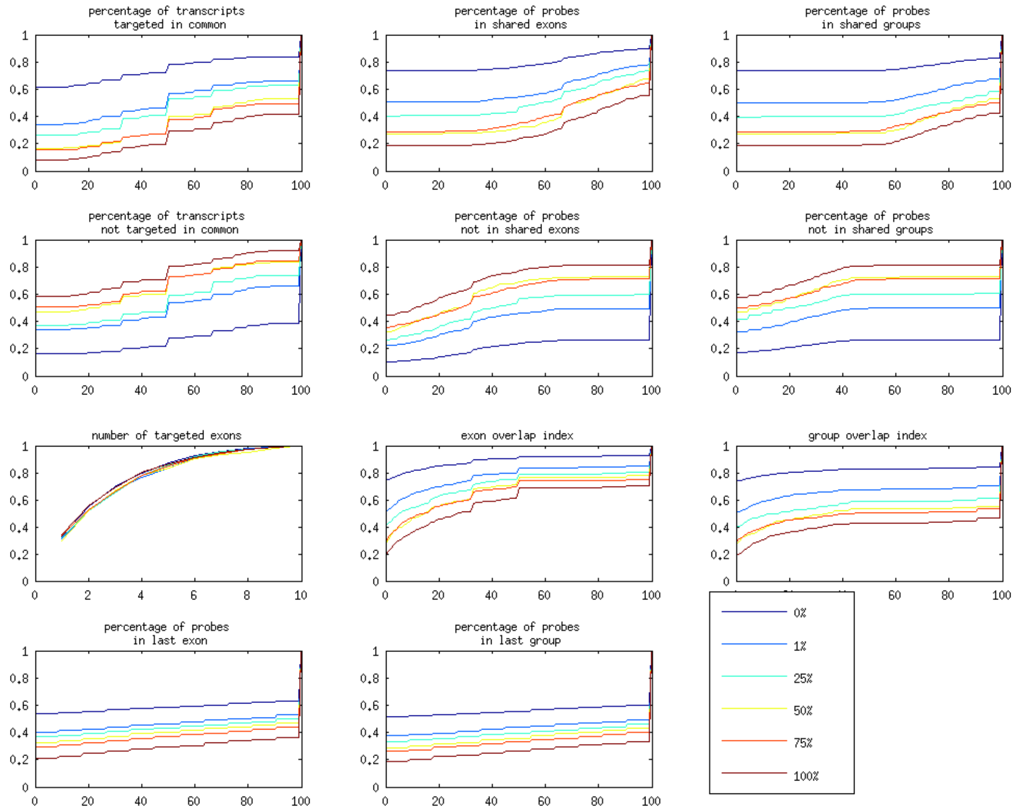


**fig.56a Relationships between similarity and localisation of probes in common exons or transcripts (Ensembl).**

**fig.56b** Relationships between similarity and localisation of probes in common exons or transcripts (Ace-View).

CHAPTER

TWO

# REFERENCE MANUALS

## 2.1 PSAWNpy

### 2.1.1 CLASSES

**Classes used by psawnPy.**

**class** `ps_class.`**`Chip`**(*myName=None*, *name=None*, *shortName=None*, *mySpeciesName=None*, *probeNb=None*, *probeLength=None*, *compName=None*, *ens47Name=None*, *ens48Name=None*, *geoName=None*)

**define a Chip model.**

```
Data attributes:
    myName --------- local chip name ('m2')
    name ----------- chip name ('Human Genome U95A Array')
    shortName ------ chip short name ('HG_U95A')
    mySpeciesName -- species name used inside program
    probeNb--------- median probe number per (pair of) probe set
    compName ------- compagny name ('Affymetrix')
    ens47Name ------ chip name in Ensembl version 1 to 47
    ens48Name ------ chip name from Ensembl version 48
    geoName -------- GEO platform name (GPL)
```

**class** `ps_class.`**`ExonList`**(*exonIndexes=[ ]*, *idList=[ ]*, *groupList=[ ]*, *exonStartArray=array([ ], dtype=uint32)*, *exonEndArray=array([ ], dtype=uint32)*, *strandArray=array([ ], dtype=int8)*, *intronStartArray=array([ ], dtype=uint32)*, *intronEndArray=array([ ], dtype=uint32)*, *transcriptsByExon=[ ]*, *transcriptIDs=[ ]*, *transcriptStarts=[ ]*, *transcriptEnds=[ ]*)

**Define a list of exons.**

```
data attributes:
    exonID ------ list of ensembl exon stable id
    exonStart --- list of exon start positions
    exonEnd ----- list of exon end positions
    exonStrand -- list of strand orientation
    groups
    intronStarts
    intronEnds
    transcriptsByExon
    transcriptIDs
    transcriptStarts
    transcriptEnds
    indexes
```

**class** ps_class.**GeneList** (*idList=[ ], startArray=array([ ], dtype=uint32), endArray=array([ ], dtype=uint32), strandArray=array([ ], dtype=int8)*)

**Define a list of genes.**

```
Data attributes:
    IDs
    starts
    ends
    strands
```

**class** ps_class.**GopList** (*idArray=array([ ], dtype=float64), regionArray=array([ ], dtype=float64), startArray=array([ ], dtype=uint32), endArray=array([ ], dtype=uint32), strandArray=array([ ], dtype=int8), upGeneDistanceArray=array([ ], dtype=uint8), downGeneDistanceArray=array([ ], dtype=uint8), upGeneIDArray=array([ ], dtype=float64), downGeneIDArray=array([ ], dtype=float64), positionNbArray=array([ ], dtype=uint8)*)

**Define a list of group of probes (GOP).**

```
Data attributes:
    IDs
    region
    starts
    ends
    strands
    upGeneDistances
    downGeneDistances
    upGeneIDs
    downGeneIDs
    positionNbs
```

**class** ps_class.**Position** (*probeID=None, regionID=None, chromosome=None, median=None, strand=None, mismatchNb=None, geneIDs=None, updownGeneIDs=None, exonIDs=None, predictedTranscriptIDs=None, updownPredictedTranscriptIDs=None, predictedExonIDs=None, localisation=None*)

**Define a position.**

```
Data attributes:
    probeID
    median
    strand
    mismatchNb

Functions:
    owningStructure -- get the surrounding element (gene or exon)
    exon ------------- test if the probe is in an exon
    intron ----------- test if the probe is in an intron
    up_or_down ------- test if the probe is up or down from a gene
```

**exon** (*arrays*)

Test if the probe is in an exon

**intron** (*arrays, geneStart, geneEnd*)

Test if the probe is in an intron

**up_or_down** (*arrays*)

Test if the probe is up or down from a gene

**class** ps_class.**PositionList** (*probeIDList=[ ], chipsList=[ ], medianArray=array([ ], dtype=uint32), strandArray=array([ ], dtype=int8), mismatchNbArray=array([ ], dtype=uint8)*)

**Define a list of genomic positions.**

```
Data attributes:
    probeIDs=probeIDList
    chips=chipsList
    medians=medianArray
    strands=strandArray
    mismatches=mismatchNbArray
```

**class** `ps_class.`**`Probe`**(*probeID=None*, *xPosition=None*, *yPosition=None*, *ensemblID=None*, *probesetID=None*, *ensProbesetID=None*, *index=None*, *sequence=None*, *targetPosition=None*, *genes4exon={}*, *genes4splice={}*, *genes4intron={}*, *genes4up={}*, *genes4down={}*, *outOfGeneNb=0*, *notInSequence=None*)

**Define a probe.**

```
Data attributes:
    probeID
    xPosition
    yPosition
    ensemblID
    probesetID
    ensProbesetID
    index
    sequence
    targetPosition
    genes4exon
    genes4intron
    genes4splice
    genes4up
    genes4down
    outOfGeneNb
    notInSequence
```

**class** `ps_class.`**`Probeset`**(*probesetID=None*, *ensGeneIDs=None*, *affyGeneIDs=None*, *probesetIndex=None*, *probeIDs=None*, *ensProbeIDs=None*, *probeIndexes=None*, *probeNb=None*, *probesetTargetLength=None*, *probesetTargetStart=None*, *probesetTargetEnd=None*, *ensExonGeneNbs=None*, *ensIntronGeneNbs=None*, *ensUpGeneNbs=None*, *ensDownGeneNbs=None*, *ensOutProbeNbs=None*, *ensNisProbeNbs=None*, *aceExonGeneNbs=None*, *aceIntronGeneNbs=None*, *aceUpGeneNbs=None*, *aceDownGeneNbs=None*, *aceOutProbeNbs=None*, *aceNisProbeNbs=None*, *ourEnsGeneIDs=None*, *ourEnsGeneNb=None*, *ourEnsProbeNb=None*, *ensGenesByProbeNb=None*, *ensGeneIDs4Ens=None*, *ourEnsGeneIDs4Ens=None*, *commonGeneIDs4Ens=None*, *ourAceGeneIDs=None*, *ourAceGeneNb=None*, *ourAceProbeNb=None*, *aceGenesByProbeNb=None*, *ourAceToEnsGeneIDs=None*, *ensGeneIDs4Ace=None*, *ourEnsGeneIDs4Ace=None*, *commonGeneIDs4Ace=None*, *ourAceToEnsGeneNbs=None*, *ourGeneIDs=None*, *ourGeneNb=None*, *ourProbeNb=None*)

**Define a probe set.**

```
Data attributes:
    probesetID
    probesetIndex
    ensGeneIDs
    affyGeneIDs
    probeNb
    probeIDs
    ensProbeIDs
```

```
probeIndexes
probesetTargetLength
probesetTargetStart
probesetTargetEnd
ensExonGeneNbs
ensIntronGeneNbs
ensUpGeneNbs
ensDownGeneNbs
ensOutProbeNbs
ensNisProbeNbs
ourEnsGeneIDs
ourEnsGeneNb
ourEnsProbeNb
ensGeneIDs4Ens
ourEnsGeneIDs4Ens
commonGeneIDs4Ens
ensGenesByProbeNb
aceExonGeneNbs
aceIntronGeneNbs
aceUpGeneNbs
aceDownGeneNbs
aceOutProbeNbs
aceNisProbeNbs
ourAceGeneIDs
ourAceGeneNb
ourAceProbeNb
aceGenesByProbeNb
ourAceToEnsGeneNbs
ourAceToEnsGeneIDs
ensGeneIDs4Ace
ourEnsGeneIDs4Ace
commonGeneIDs4Ace
ourGeneIDs
ourGeneNb
ourProbeNb
```

**class** `ps_class.`**`ProbesetList`**(*probesetID=None*, *probesetIndex=None*, *probeNb=None*, *ensGeneIDnb=None*, *affyGeneIDnb=None*, *ensExonGeneNbs=None*, *ensIntronGeneNbs=None*, *ensUpGeneNbs=None*, *ensDownGeneNbs=None*, *ensOutProbeNbs=None*, *ensNisProbeNbs=None*, *aceExonGeneNbs=None*, *aceIntronGeneNbs=None*, *aceUpGeneNbs=None*, *aceDownGeneNbs=None*, *aceOutProbeNbs=None*, *aceNisProbeNbs=None*, *ourEnsGeneNb=None*, *ourEnsProbeNb=None*, *ourAceGeneNb=None*, *ourAceProbeNb=None*, *ensGeneIDs4EnsNb=None*, *ourEnsGeneIDs4EnsNb=None*, *commonGeneIDs4EnsNb=None*, *ourEnsGeneIDs4AceNb=None*, *ensGeneIDs4AceNb=None*, *ourEnsGeneIDs4AceNB=None*, *commonGeneIDs4AceNb=None*, *ourEnsTargetedExons=None*, *ourEnsTargetedGroups=None*, *ourEnsTargetedTranscripts=None*, *ourEnsNotTargetedTranscripts=None*, *ourAceTargetedExons=None*, *ourAceTargetedGroups=None*, *ourAceTargetedTranscripts=None*, *ourAceNotTargetedTranscripts=None*)

**Define a list of probe sets.**

```
Data attributes:
    probesetNb=len(probesetID)
    probesetID=probesetID
```

```
probesetIndex=probesetIndex
probeNb=probeNb
ensGeneIDnb=ensGeneIDnb
affyGeneIDnb=affyGeneIDnb
ensExonGeneNbs=ensExonGeneNbs
ensIntronGeneNbs=ensIntronGeneNbs
ensUpGeneNbs=ensUpGeneNbs
ensDownGeneNbs=ensDownGeneNbs
ensOutProbeNbs=ensOutProbeNbs
ensNisProbeNbs=ensNisProbeNbs
ourEnsGeneNb=ourEnsGeneNb
ourEnsProbeNb=ourEnsProbeNb
ensGeneIDs4EnsNb=ensGeneIDs4EnsNb
ourEnsGeneIDs4EnsNb=ourEnsGeneIDs4EnsNb
commonGeneIDs4EnsNb=commonGeneIDs4EnsNb
ourEnsTargetedExons=ourEnsTargetedExons
ourEnsTargetedGroups=ourEnsTargetedGroups
ourEnsTargetedTranscripts=ourEnsTargetedTranscripts
ourEnsNotTargetedTranscripts=ourEnsNotTargetedTranscripts
aceExonGeneNbs=aceExonGeneNbs
aceIntronGeneNbs=aceIntronGeneNbs
aceUpGeneNbs=aceUpGeneNbs
aceDownGeneNbs=aceDownGeneNbs
aceOutProbeNbs=aceOutProbeNbs
aceNisProbeNbs=aceNisProbeNbs
ourAceGeneNb=ourAceGeneNb
ourAceProbeNb=ourAceProbeNb
ensGeneIDs4AceNb=ensGeneIDs4AceNb
ourEnsGeneIDs4AceNb=ourEnsGeneIDs4AceNb
commonGeneIDs4AceNb=commonGeneIDs4AceNb
ourAceTargetedExons=ourAceTargetedExons
ourAceTargetedGroups=ourAceTargetedGroups
ourAceTargetedTranscripts=ourAceTargetedTranscripts
ourAceNotTargetedTranscripts=ourAceNotTargetedTranscripts
```

**class** `ps_class.`**`Species`**(*myName=None*, *officialName=None*, *ensName=None*)

**Define a Species.**

```
Data attributes:
    myName ----- -- species name used inside program
    officialName -- scientific latin name
    ensName ------- name used in Ensembl tables
```

**class** `ps_class.`**`StructureList`**(*idList=[ ]*, *startArray=array([ ]*, *dtype=uint32)*, *endArray=array([ ]*, *dtype=uint32)*, *strandArray=array([ ]*, *dtype=int8)*)

**Define a list of genomic structures.**

```
Data attributes:
    IDs=idList
    starts=startArray
    ends=endArray
    strands=strandArray
```

**class** `ps_class.`**`TargetedGene`**(*probeIndexes=[ ]*, *probeLocalisations=[ ]*, *firstStructureIndexes=[ ]*, *firstStructureGroups=[ ]*, *sndStructureIndexes=[ ]*, *sndStructureGroups=[ ]*, *firstStructureIDs=[ ]*, *probePositions=[ ]*, *probeMismatchNbs=[ ]*, *probeRepetitionNbs=[ ]*, *probeStrands=0*, *inExonProbeNb=0*, *inSpliceProbeNb=0*, *inIntronProbeNb=0*, *upProbeNb=0*, *downProbeNb=0*, *targetedTranscripts=None*, *exonSet=None*, *groupSet=None*, *notTargetedTranscripts=None*, *ensemblGenes=None*)

**Define a list of probes targeting a gene.**

```
Data attributes:
    probeIndexes
    probeLocalisations
    firstStructureIndexes
    firstStructureGroups
    sndStructureIndexes
    sndStructureGroups
    firstStructureIDs
    probePositions
    probeRepetitionNbs
    probeStrands
    probeMismatchNbs
    inExonProbeNb
    inSpliceProbeNb
    inIntronProbeNb
    upProbeNb
    downProbeNb
    exonSet
    groupSet
    targetedTranscripts
    notTargetedTranscripts
    ensemblGenes

Function:
    fill_info
```

**class** `ps_class.`**`TargetingProbeset`**(*probesetNames={}*, *probesetIndexes={}*, *targetedExons={}*, *targetedGroups={}*, *targetedTranscripts={}*, *notTargetedTranscripts={}*, *inGeneProbeNbs={}*, *notInExonProbeNbs={}*)

**Define a dictionnary of targets** indexed on the number of targeting probes**

```
Data attributes:
    probesetNames
    probesetIndexes
    targetedExons
    targetedGroups
    targetedTranscripts
    notTargetedTranscripts
    inGeneProbeNbs
    notInExonProbeNbs
```

**class** `ps_class.`**`Targets`**(*repetition=[ ]*, *IDs=[ ]*, *indexes=[ ]*, *groups=[ ]*, *mismatchNbs=[ ]*, *positions=[ ]*, *strands=[ ]*)

**Define a target.**

```
Data attributes:
    repetition
    IDs
    indexes
```

```
    groups
    positions
    strands
    mismatchNbs
```

**class** `ps_class.`**`Transcript`** (*ID=None*, *geneID=None*, *start=None*, *end=None*, *region=None*, *chromosome=None*, *sequence=None*, *strand=None*)

**Define a transcript.**

```
Data attributes:
    transcriptID -- transcript ID
    geneID -------- gene ID
    start --------- genomic start position of transcript
    end ----------- genomic end position of transcript
    region -------- Ensembl region
    chromosome ---- chromosome
    sequence ------ transcript sequence
    strand -------- gene strandedness
```

**class** `ps_class.`**`TranscriptList`** (*idList=[ ]*, *startArray=array([ ]*, *dtype=uint32)*, *endArray=array([ ]*, *dtype=uint32)*, *strandArray=array([ ]*, *dtype=int8)*)

**Define a list of transcripts.**

```
Data attributes:
    IDs=idList
    starts=startArray
    ends=endArray
    strands=strandArray
```

### 2.1.2 BINTOOLS

**Functions for testing position membership and processing exons.**

`bintools.`**`isInStructure`** (*start*, *end*, *strand*, *starts*, *ends*, *strands*, *overlapFlag*)

**Find the structure(s) wich contains the tested structure.**

```
Arguments:
    start    ----- starting position of the tested structure
    end      ----- ending position of the tested structure
    strand ------- strandedness (-1 or -1) of the tested structure
    starts ------- array of starting coordinates of the scanned structures
    ends --------- array of ending coordinates of the scanned structures
    strands ------ array of strandedness of the scanned structures
    overlapFlag -- allows to retrieve several overlapping structures

Return:
    indexes -- array of indexe(s) of structure(s) containing the tested structure
```

`bintools.`**`isOverlapStructure`** (*start*, *end*, *strand*, *starts*, *ends*, *strands*, *overlapFlag*)

**Find the structure(s) wich overlap the tested structure.**

```
Arguments:
    start -------- starting position of the tested structure
    end ---------- ending position of the tested structure
    strand ------- strandedness (-1 or -1) of the tested structure
    starts ------- array of starting coordinates of the scanned structures
    ends --------- array of ending coordinates of the scanned structures
    strands ------ array of strandedness of the scanned structures
```

```
        overlapFlag -- allows to retrieve several overlapping structures

    Return:
        indexes -- array of indexe(s) of structure(s) overlaping the tested structure
```

bintools.**owningStructure**(*position*, *strand*, *starts*, *ends*, *strands*, *overlapFlag*)

**Find the structure(s) wich contains the tested position.**

```
    Arguments:
        position ----- coordinate of the tested position
        strand ------- strandedness (-1 or -1) of the tested position
        starts ------- array of starting coordinates of the scanned structures
        ends --------- array of ending coordinates of the scanned structures
        strands ------ array of strandedness of the scanned structures
        overlapFlag -- allows to retrieve several overlapping structures

    Return:
        indexes -- array of indexe(s) of structure(s) containing the tested position
```

bintools.**process_exons**(*exonIDs=[ ]*, *exonStarts=[ ]*, *exonEnds=[ ]*, *exonStrands=[ ]*, *transcriptIDs=[ ]*)

**Order exons and eliminate doublons.**

```
    Keywords arguments:
        exonIDs -------- list of exon IDs
        exonStarts ----- list of exon starting positions
        exonEnds ------- list of exon ending positions
        exonStrands ---- list of exon strandedness
        transcriptIDs -- list of transcript IDs

    Return ordered list without doublons:
        exonIDList --------- list of exon IDs
        exonStarts --------- array of exon starting positions
        exonEnds ----------- array of exon ending positions
        exonStrands -------- array of exon strandedness
        transcriptsByExon -- list of sets of transcripts
        transcriptIDs ------ list of transcript IDs
        transcriptStarts --- array of transcript starts (smallest targeting exon start)
        transcriptEnds ----- array of transcript ends (largest targeting exon end)
        exonIndexes -------- array of indexes indicating the rank of exon start positions relative
                             to the 5' gene start position
        exonGroups --------- indicate for each exon, the group it belongs to
                             (a chain of overlaping exons is a group)
        intronStarts ------- start positions of introns defined as inter-group intevals
        intronEnds --------- end positions of introns defined as inter-group intevals
```

### 2.1.3 DBTOOLS

**Functions for creating Berkeley data bases.**

dbtools.**makebt**(*dict=None*, *keys=None*, *values=None*, *path=None*, *newFlag=0*, *objFlag=0*, *log=0*)

**Create a btree Berkeley database.**

```
    Arguments:
        dict ----- dictionary to be saved
        keys ----- list of keys
        values --- list of values
        path ----- path of the berkeley database file to be created
```

```
newFlag -- indicates if the data base is new (newFlag=1 and in this case if it exists,
           it is erased) or is to be updated (newFlag=0)
objFlag -- indicate if the value must be stored as a string (0) or
           as an object by using cPickle
log ------- handle of a log file for recording messages
```

dbtools.**write_obj_db**(*lines*, *objClass*, *objItems*, *objType*, *path*, *newFlag*, *log=0*)

**Prepare data for creating data base with objects as values.**

```
Arguments:
lines ----- is a list of tuples (line) which is ordered on line[0] used as key
            to construct a dictionary. Several lines may have the same key. In this
            case, class properties are lists.
objClass -- class of the object stored as a  value in the constructed dictionary
objItems -- a string which is evaluated to assign values stored in a dictionary
            (item[1],item[2],...) to the right object property
path ------ name of the btree berkeley data base constructed with the keys and values lists
newFlag  -- indicates if the data base is new (newFlag=1 and in this case if it exists,
            it is erased) or is to be updated (newFlag=0)
log ------- handle of a log file for recording messages
```

dbtools.**write_str_db**(*lines*, *path*, *newFlag*, *log=0*)

**Prepare data for creating data base with strings as values.**

```
Arguments:
lines ----- is a list of tuples (line) which is ordered on line[0] used as key
            to construct a dictionary. All lines have different keys.
path ------ name of the btree berkeley data base constructed with the keys and values lists
newFlag  -- indicates if the data base is new (newFlag=1 and in this case if it exists,
            it is erased) or is to be updated (newFlag=0)
log ------- handle of a log file for recording messages
```

### 2.1.4 MAIN SCRIPT

**Control data import.**

```
Parameters:
actions       -- a list of action designed by numbers
        1 : make chip.bkdb
        2 : make species.bkdb
        3 : make chip_probe.bkdb
mySpeciesName -- species name used inside program
myChipName    -- chip name used inside program
ensVersion    -- Ensembl version to be interrogated

Actions:

1 --
Example:
pscontrol_chip [3] mouse m8 62_37o
```

### 2.1.5 PS_ACEVIEW

**Process AceView data.**

ps_aceview.**ensembl_genes**(*species*, *log=0*)

**Find overlaping genes between Ensembl and AceView.**

```
Arguments:
    species -- species
    log ------ handle of a log file for recording messages

Input:
    %species_genes_by_ensembl_region.bkdb
    %species_genes_by_region.bkdb

Output:
    species_ensembl_genes_by_gene.bkdb
        key ==== AceView gene id
        value == list of Ensembl genes ids
```

ps_aceview.**genes_exons**(*species*, *aceVersion*, *log=0*)

**Process AceView gene files.**

```
Arguments:
    species    -- species
    aceVersion -- AceView version
    log --------- handle of a log file for recording messages

Input:
    %species_region_by_chromosome.bkdb
    x1.genes_gff.%chromosome.gff

Output:
    create %species_genes_by_ensembl_region.bkdb
        key ==== Ensembl region id
        value == GeneList object
    create %species_exons_by_gene.bkdb
        key ==== AceView gene id
        value == ExonList
    update %species_transcript_sequence.bkdb
        key ==== AceView transcript id
        value == Transcript object
    create %species_transcripts_by_gene.bkdb
        key ==== AceView gene id
        value == list of AceView transcript ids
    create %species_transcripts_by_exon.bkdb
        key ==== AceView exon id
        value == list of AceView transcript ids
```

ps_aceview.**transcript_sequence**(*species*, *aceVersion*, *log=0*)

**Read AceView files containing transcript sequences for each chromosomes.**

```
Arguments:
    species ----- species
    aceVersion -- AceView version
    log --------- handle of a log file for recording messages

Input:
    %species_region_by_chromosome.bkdb

Output:
    creates %species_transcripts_sequence.bkdb
        key ==== AceView transcript id
```

```
        value == Transcript object
```

### 2.1.6 PS_ENSEMBL

**Functions for importing Ensembl data.**

ps_ensembl.**chromosome_by_region**(*ensTable*, *species*, *host='ensembldb.ensembl.org'*, *port=5306*, *user='anonymous'*, *pswd=''*, *log=0*)

**Find chromosomes by Ensembl region of a particular species.**

```
Arguments:
    ensTable -- Ensembl table to be interrogated by MySQL
    species --- species
    host ------ Ensembl database address
    port ------ Ensembl port
    user ------ user name
    pswd ------ user pasword
    log ------- handle of a log file for recording messages

Output:
    create %species_chromosomes_by_region.bkdb
        key ==== Ensembl region id
        value == chomosome (str)
    create %species_region_by_chromosome.bkdb
        key ==== chomosome (str)
        value == Ensembl region id
```

ps_ensembl.**exons_by_gene**(*ensTable*, *species*, *host='ensembldb.ensembl.org'*, *port=5306*, *user='anonymous'*, *pswd=''*, *log=0*)

**Find exon(s) of Ensembl genes of a particular species.**

```
Arguments:
    ensTable -- Ensembl table to be interrogated by MySQL
    species --- species
    host ------ Ensembl database address
    port ------ Ensembl port
    user ------ user name
    pswd ------ user pasword
    log ------- handle of a log file for recording messages

Output:
    create %species_exons_by_gene.bkdb
        key ==== Ensembl gene stable id
        value == ExonList
```

ps_ensembl.**exons_by_predicted_transcript**(*ensTable*, *species*, *host='ensembldb.ensembl.org'*, *port=5306*, *user='anonymous'*, *pswd=''*, *log=0*)

**Find exon(s) in Ensembl predicted transcripts of a particular species.**

```
Arguments:
    ensTable -- Ensembl table to be interrogated by MySQL
    species --- species
    host ------ Ensembl database address
    port ------ Ensembl port
    user ------ user name
    pswd ------ user pasword
    log ------- handle of a log file for recording messages
```

```
Output:
    create %species_exons_by_predicted_transcripts.bkdb
        key ==== Ensembl predicted transcript id
        value == ExonList
```

ps_ensembl.**genes_by_region**(*ensTable*, *species*, *host='ensembldb.ensembl.org'*, *port=5306*, *user='anonymous'*, *pswd=''*, *log=0*)

**Find genes(s) in Ensembl regions of a particular species.**

```
Arguments:
    ensTable -- Ensembl table to be interrogated by MySQL
    species --- species
    host ------ Ensembl database address
    port ------ Ensembl port
    user ------ user name
    pswd ------ user pasword
    log ------- handle of a log file for recording messages

Output:
    create %species_genes_by_region.bkdb
        key ==== Ensembl stable region id
        value == GeneList
```

ps_ensembl.**predicted_transcripts_by_region**(*ensTable*, *species*, *host='ensembldb.ensembl.org'*, *port=5306*, *user='anonymous'*, *pswd=''*, *log=0*)

**Find predicted transcripts by Ensembl region of a particular species.**

```
Arguments:
    ensTable -- Ensembl table to be interrogated by MySQL
    species --- species
    host ------ Ensembl database address
    port ------ Ensembl port
    user ------ user name
    pswd ------ user pasword
    log ------- handle of a log file for recording messages

Output:
    create %species_predicted_transcripts_by_region.bkdb
        key ==== Ensembl region id
        value == TranscriptList
```

ps_ensembl.**transcript**(*tscriptFile*, *ncbi*, *species*, *log=0*)

**Extract data from an Ensembl fasta cDNA file.**

```
Arguments:
    tscriptFile -- Ensembl fasta cDNA file
    ncbi --------- ncbi version
    species ------ species
    log ---------- handle of a log file for recording messages

Input:
    %species_region_by_chromosome.bkdb

Output:
    create %species_region_by_chromosome_log.txt
    create %species_transcript_sequence.bkdb
        key ==== Ensembl transcript id
        value == Transcript object
```

ps_ensembl.**transcripts_by_exon**(*ensTable*, *species*, *host='ensembldb.ensembl.org'*, *port=5306*, *user='anonymous'*, *pswd=''*, *log=0*)

**Find transcript(s) targeted by exons of Ensembl genes of a particular species.**

```
Arguments:
    ensTable -- Ensembl table to be interrogated by MySQL
    species --- species
    host ------ Ensembl database address
    port ------ Ensembl port
    user ------ user name
    pswd ------ user pasword
    log ------- handle of a log file for recording messages

Output:
    create %species_transcripts_by_exons.bkdb
        key ==== Ensembl exon stable id
        value == TranscriptList object
```

ps_ensembl.**transcripts_by_gene**(*ensTable*, *species*, *host='ensembldb.ensembl.org'*, *port=5306*, *user='anonymous'*, *pswd=''*, *log=0*)

**Find transcripts by Ensembl region of a particular species.**

```
Arguments:
    ensTable -- Ensembl table to be interrogated by MySQL
    species --- species
    host ------ Ensembl database address
    port ------ Ensembl port
    user ------ user name
    pswd ------ user pasword
    log ------- handle of a log file for recording messages

Output:
    create %species_transcripts_by_region.bkdb
        key ==== Ensembl region id
        value == TranscriptList
```

### 2.1.7 PS_EXPORT

**Write ouptut files.**

ps_export.**write_aceview_genes**(*species*, *log=0*)

**Write several probesets_by_gene files indexed on the number of targeting probes.**

```
Arguments:
    species --- species name
    chipName -- chip name
    log ------- handle of a log file for recording messages

Input:
    %species_genes_by_region.bkdb
    %species_ensembl_genes_by_gene.bkdb

Output:
    %species_ens_by_ace_gene.txt
```

ps_export.**write_probeset_list**(*species*, *chipName*, *log=0*)

**Write several probesets_by_gene files indexed on the number of targeting probes.**

```
Arguments:
    species --- species name
    chipName -- chip name
    log ------- handle of a log file for recording messages

Input:
    %chip_probeset.dump

Output:
    %chip_probesets_ensembl.txt
    %chip_probesets_aceview.txt
```

ps_export.**write_probesets_by_gene**(*species*, *chipName*, *log=0*)

**Write several probesets_by_gene files indexed on the number of targeting probes.**

```
Arguments:
    species --- species name
    chipName -- chip name
    log ------- handle of a log file for recording messages

Input:
    %chip_probesets_by_gene.bkdb
    %chip_probe.bkdb

Output:
    ensembl_%chip_probesets_by_gene_%probeNb.txt
    aceview_%chip_probesets_by_gene_%probeNb.txt
```

### 2.1.8 PS_IMPORT

**Create berkeley databases for chips, species, probes and probe sets.**

ps_import.**fill_probesetdb**(*ensTable*, *ensVersion*, *species*, *ensSpecies*, *chipName*, *ensChipName*, *host='ensembldb.ensembl.org'*, *port=5306*, *user='anonymous'*, *pswd='‘*, *log=0*)

**Update probeset database.**

```
Arguments:
    ensTable ---- Ensembl table to be interrogated by MySQL
    ensVersion -- Ensembl version
    species ----- species name
    host -------- Ensembl database address
    port -------- Ensembl port
    user -------- user name
    pswd -------- user password
    log --------- handle of a log file for recording messages

Output:
    Update %chip_probeset.bkdb
        key ==== probe set id
        value == Probeset object
```

ps_import.**make_chipdb**(*newFlag*, *log=0*)

**Create chips database.**

```
Arguments:
    newFlag  -- indicates if the data base is made from scratch (newFlag=1, existing
                data base is erased) or is to be created or updated (newFlag=0)
```

```
    log ------- handle of a log file for recording messages

Input:
    chip.txt

Output:
    Create chip.bkdb
        key ==== chip name
        value == Chip object
```

ps_import.**make_probedb**(*ensTable*, *ensVersion*, *species*, *chipName*, *ensChipName*, *host='ensembldb.ensembl.org'*, *port=5306*, *user='anonymous'*, *pswd=''*, *log=0*)

**Create probe database.**

```
Arguments:
    ensTable ---- Ensembl table to be interrogated by MySQL
    ensVersion -- Ensembl version
    species ---- species
    host ------- Ensembl database address
    port ------- Ensembl port
    user ------- user name
    pswd ------- user pasword
    log -------- handle of a log file for recording messages

Output:
    Create %chip_probe.bkdb
        key ==== probe id
        value == Probe object
```

ps_import.**make_probesetdb**(*chipList*, *log=0*)

**Create probeset database.**

```
Arguments:
    chipList  -- list the chips to be used for creating probe set databases
    log ------- handle of a log file for recording messages

Input:
    %chip.bkdb
    %chip_probeset.txt

Output:
    Create %chip_probeset.bkdb
        key ==== probe set id
        value == Probeset object
```

ps_import.**make_speciesdb**(*newFlag*, *log=0*)

**Create species database.**

```
Arguments:
    newFlag  -- indicates if the data base is made from scratch (newFlag=1, existing
                data base is erased) or is to be created or updated (newFlag=0)
    log ------- handle of a log file for recording messages

Input:
    species.txt

Output:
    species.bkdb
```

```
        key ==== species name
        value == Species object
```

ps_import.**probeset**(*probeFileName*, *species*, *chipName*, *probeLength*, *log=0*)

**Update probeset database (fill probe information).**

```
Arguments:
    probeFileName -- Affymetrix file describing probes
    species -------- species name
    chipName  ------ chip name
    probeLength ---- probe length
    log ------- handle of a log file for recording messages

Input:
    %chip_probe.bkdb
    %chip_probeset.txt

Output:
    Update %chip_probeset.bkdb
        key ==== probe set id
        value == Probeset object
```

ps_import.**set_probeset_info**(*probeset*, *probeIDs*, *position*, *probeLength*, *type*)

**Update probe set information (calculate target genomic start and end).**

```
Arguments:
    probeset ----- Probeset object
    probeIDs ----- list of probe ids
    position ----- list of probe position in transcript or in genome
    probeLength -- probe length
    type --------- 'transcript' or 'genome'
```

### 2.1.9 PS_POSITION

**Functions for processing positions.**

ps_position.**gop_by_region**(*species*, *log=0*)

**Construct groups of probes (GOPs)**

```
Probes that are less than 2 kb apart and that are outside gene limits +/- 2 kb
are grouped and given an Ensmelb  gene-like name (GOPMUSG00000000001 ...)

Arguments:
    species -- species name
    log -------- handle of a log file for recording messages

Input:
    %species_genes_by_region.bkdb (Ensembl genes positions)
    %species_genes_by_ensembl_region.bkdb (AceView genes positions)
    %species_positions_by_region.bkdb
    %species_chromosomes_by_region.bkdb

Output:
    create %chip_gops_by_region.bkdb (chips used are those used to search positions)
```

ps_position.**merge_positions**(*species*, *chipList*, *region*, *log=0*)

**Merge positions by ensembl region for a species.**

```
creates one database merging the probe position of all the chip sets of a species
+1 (Plus) and -1 (Minus) strands position are separated in order to have position ordered insi
each category

Arguments:
    species --- species name
    chipList -- list of chips
    log -------- handle of a log file for recording messages

Input:
    %chip_positions_by_region.bkdb

Output:
    Create %species_positions_by_region.bkdb
        key ==== Ensembl region id
        value == PositionList object
```

ps_position.**positions_by_region**(*species*, *myChipList*, *ensVersion*, *ensTable*, *ensChipList*, *host='ensembldb.ensembl.org'*, *port=5306*, *user='anonymous'*, *pswd=''*, *log=0*)

**Find positions by Ensembl region for a list of chips.**

```
Arguments:
    species ----- species name
    myChipList -- list of chip names
    ensVersion -- Ensembl version
    ensTable ---- Ensembl table to be interrogated by MySQL
    host ------- Ensembl database address
    port ------- Ensembl port
    user ------- user name
    pswd ------- user pasword
    log -------- handle of a log file for recording messages

Output:
    Create %chip_positions_by_region.bkdb
        key ==== Ensembl region id
        value == PositionList object
```

### 2.1.10 PS_PROBESET

**Functions for assigning probe set to gene(s) according to the number of targeting probes.**

ps_probeset.**assign_probeset**(*species*, *chipName*, *dbType*, *psRange=0*, *aceVersion=''*, *log=<ufunc 'log'>*)

**Assign probe set to gene(s) or GOP(s) according to the number of targeting probes.**

```
Arguments:
    species --- species name
    chipName -- chip name
    dbType ---- either 'ensembl' or 'aceview'
    psRankge -- range of probe set to be processed
    aceVersion -- AceView version
    log -------- handle of a log file for recording messages

Inputs:
    %species_transcripts_by_gene.bkdb
    %species_transcript_sequences.bkdb
    %species_transcripts_by_exon.bkdb
```

```
        %species_ensembl_genes_by_gene.bkdb (if dbType is aceview)


    Outputs:
        update %chip_probeset.bkdb
        update %chip_probe.bkdb

    Log files:
        %chip_ensembl_assign_probeset_log.txt or
        %chip_aceview_assign_probeset_log.txt (according to dbTYpe)
```

ps_probeset.**make_probeset_list**(*species*, *chipName*)

**Make a dump of probesets.**

```
    Atributes:
        species --- species name
        chipName -- chip name

    Input:
        %chip_probeset.bkdb

    Output:
        %chip_probeset.dump
```

ps_probeset.**position_gopmapping**(*species*, *chipName*, *region*, *log=0*)

**Map positions to group of probes (GOPs).**

```
    Arguments:
        species --- species name
        chipName -- chip name
        dbType ---- either 'ensemble' of 'aceview'
        region ---- Ensembl region id
        log -------- handle of a log file for recording messages

    Input:
        %chip_gops__by_region.bkdb
        %chip_positions_by_region.bkdb

    Output:
        update %chip_probe.bkdb (Ensembl or AceView database according to dbType argument)

    Log files:
        %species_ensembl_position_gopmapping_log.txt
```

ps_probeset.**position_mapping**(*species*, *chipName*, *dbType*, *region*, *log=0*)

**Map positions to gene structures (exon, intron, upstream or downstream).**

```
    Arguments:
        species --- species name
        chipName -- chip name
        dbType ---- either 'ensemble' of 'aceview'
        region ---- Ensembl region id
        log -------- handle of a log file for recording messages

    Input:
        %species_genes_by_region.bkdb or
        %species_genes_by_ensembl_region.bkdb (Ensembl or AceView database according to dbType arg
        %chip_positions_by_region.bkdb
```

```
Output:
    update %species_exons_by_gene.bkdb (Ensembl or AceView database according to dbType argume
    update %chip_probe.bkdb (Ensembl or AceView database according to dbType argument)

Log files:
    %chip_ensembl_position_mapping_log.txt or
    %chip_aceview_position_mapping_log.txt (according to dbType argument)
```

`ps_probeset.`**`probesets_by_gene`**(*species*, *chipName*, *log=0*)

**Find probe sets targeting genes.**

```
Arguments:
    species --- species name
    chipName -- chip name

Inputs:
    %chip_probeset_bkdb

Output:
    create %chip_probesets_by_gene.bkdb (for Ensembl)
    create %chip_probesets_by_gene.bkdb (for AceView)
```

### 2.1.11 SETENVIRON

**Set environment variables containing data paths according to the host used.**

## 2.2 PSAWNml

### 2.2.1 CALCULATE_LIMITS

```
============================
 FUNCTION CALCULATE_LIMITS
============================

 CALCULATE_LIMITS uses several small networks (1024 comparisons : 32x32 biol cond)
 to find the distributions of p-value of overlaping between neighbourhood and of
 positive and negative correlation of probesets that target the same groups of transcripts

INPUT PARAMETERS

 1        ModelRank: chip model rank
 2          FigList: indicates figures that are to be displayed (set it to [] in order
 3 FirstNetRankList: first list of networks
 4       PvCorrRank: pv(overlap) is calculated for corr limit >[0,40,50,60]. PvCorrRank
                     indicates the corr limit to be used, by giving its index in
                     the corr list(,[0,40,50,60])
 5         MeanFlag: indicates if limit is calculated from mean of four values (single and
                     multiple targeted genes, and InSim and OutSim) or only from single
                     targeted genes and InSim.
 6          ValFlag: indicates if all values of corr, anti and pv of each pair are used, or
                     only a single derived value (mean - std)
 varargin:
 5     NoQlimitFlag: indicates if the second group of network is a set of no qlimit network
 6   SndNetRankList: second list of networks
```

```
 FIGURES 30 to 39
```

### 2.2.2 CALCULATE_NODESIM

```
============================
 FUNCTION CALCULATE_NODESIM
============================

CALCULATE_NODESIM calculates positive (CORR) and negative (ANTI) correlation,
 and p-value (PV) ofnode neighbourhood similarity between different categories of
 paired probesets that target the same genes (duplicates) in several networks.
 Categories are:
 Pairs of probesets targeting a single gene
    Pairs of probesets inside exons of the same gene (single)
    Pairs of probesets outside exons of the same gene (single_testout)
    Pairs of randomly matched probesets present in ~single and targeting genes with more
      or = than max(1,ProbeNbLimit-2 probes (single_testhigh)
    Pairs of randomly matched probesets, one present ~testhigh and the other in ~testLow
      (single_testlowhigh)
    Pairs of randomly matched probesets present in ~single targeting with genes with less
      than 3 probes (single_testlow)
 Pairs of probesets targeting several genes
    Pairs of probesets inside exons of the same genes (multiple)
    Pairs of probesets outside exons of the same genes (multiple_testout)
    Pairs of randomly matched probesets present in ~multiple and targeting genes with more
      or = than max(1,ProbeNbLimit-2 probes (multiple_testhigh)
    Pairs of randomly matched probesets, one present ~testhigh and the other in ~testLow
      (multiple_testlowhigh)
    Pairs of randomly matched probesets present in ~multiple targeting with genes with less
      than 3 probes (multiple_testlow)
    Pairs of probesets inside exons of the same gene(s)

INPUT PARAMETERS
1       TestFlag: if =1 CORR, ANTI and PV distributions are calculated on all
                  categories to study their differential properties;
                  if =0 CORR, ANTI and PV distributions are calculated only on single
                  category to find corresponding test limits used to determine if a
                  particular pair of probeset must be considered as similar(that is
                  targeting the same group of transcript(s)
 2 NotFoundFlag: indicates that duplicates not found in a first round are processed
                  (TestFlag is equal to 0 in this case)
 3 ProbeNbLimit: minimal number of probes targeting a gene (used to make
                  a bipartition of genes)
 4      ChipRank: rankf of chip model
 5  NetRankList: a list of nets, designed by their rank, used to calculate node neighbourhodd
                          smilarity
 6 PvCorrLimits: Indicates the corr values that must be used to select the
                                  probesets used to calculate Pv of node neighbourhood similarity
 7       AceFlag: indicates if AceView data are used
 8      IdemFlag: indicates if probeset order is identical in file used by PsawnPy et in
                  networks (CVM)

OUTPUT FILES

 For each network, a file containing the Sim variable filled by function NODESIM variable
 is written in a mat file.
```

```
INTERNAL FUNCTION

''''''''''''''''''
 FUNCTION NODESIM
''''''''''''''''''

INPUT PARAMETERS
 1    ChipRank: rank of chip model
 2      NetRank: rank of the current net
 3 ProbeNbLimit: minimal number of probes targeting a gene (used to make a
                 bipartition of genes)
 4          Dup: samples of pairs of probesets targeting the same gene(s)
 5 MultipleFlag: used if ProbeNbLImit==1 => allows to consider probeset with
                 only one target (MultipleFlag=0) or with several targets (MultipleFlag=1)
 6      DupType: indicates different type of duplicate

OUTPUT FILES
 For each type of duplicate a file is written. Example:
 'm%ChipRank_n%NetRank_nodesim_probenb%ProbeNbLimit_multiple.mat'
 This file contains the structured variable Sim with the following fields.

            Sim.corr: positive correlations values for each pair of probesets
            Sim.anti: negative correlations values for each pair of probesets
     Sim.firstPsRank: probeset ranks of the first probeset in each pair
       Sim.sndPsRank: probeset ranks of the second probeset in each pair
 Sim.commonNodeNb{i}: common number of neighbouhrs
  Sim.firstNodeNb{i}: number of neighbouhrs for the first probeset
    Sim.sndNodeNb{i}: number of neighbouhrs for the second probeset
      Sim.overlap{i}: percentage overlap (common number * 100 / min(firstNodeNb,sndNodeNb))
           Sim.pv{i}: p-value calculated with hypergeometric distribution;
```

### 2.2.3 CALCULATE_PEARSON

```
============================
 FUNCTION CALCULATE_PEARSON
============================

 CALCULAT_PEARSON calculates the Pearson's correlation coefficient on all pairs of probe
 sets referenced in dup files

INPUT PARAMETERS
 1      Species: species
 2     ChipRank: chip rank
 3 ProbeNbLimit: minimal number of probes targeting a gene (used to make
                 a bipartition of genes)
 4     TestFlag: if =1 CORR, ANTI and PV distributions are calculated on all
                 categories to study their differential properties;
                 if =0 CORR, ANTI and PV distributions are calculated only on single
                 category to find corresponding test limits used to determine if a
                 particular pair of probeset must be considered as similar(that is
                 targeting the same group of transcript(s)
 5 NotFoundFlag: indicates that duplicates not found in a first round are processed
                 (TestFlag is equal to 0 in this case)
 6     IdemFlag: indicates if probeset order is identical in file used by PsawnPy et in
                 networks (CVM)
```

### 2.2.4 DEMO_PS

```
==================
FUNCTION DEMO_PS
==================

 DEMO_PS load global variables used by other PSAWNml scripts
 demo must be run from inside the main directory:
 'cd .../psawnml/'
 'demo_ps'

 GLOBAL VARIABLES
 K.dir contains directory paths and must be edited to match the existing directories
 K.chip contains information on chips
```

### 2.2.5 DISPLAY_NODESIM

```
==========================
 FUNCTION DISPLAY_NODESIM
==========================

DISPLAY_NODESIM displays figures related to statistics on similarity between different
 pairs of probesets

INPUT PARAMETERS
 1 ProbeNbLimit: minimal number of probes targeting a gene
                 (used to make a bipartition of genes)
 2    ModelRank: chip rank model
 3      NetRank: net rank
 4    FigureNbs: list of figures to be displayed
 5        SimNb:  number of similarity types to be displayed in figure 4 (from 1 to SimNb)
                order is (Sim, OutSim, HighSim, LowHighSim, LowSim)

 FIGURES 19 to 29
```

### 2.2.6 FILL_PSINFO

```
======================
 FUNCTION FILL_PSINFO
======================

FILL_PSINFO: for a given nb of probes (ProbeNbLimit) recover for each probeset information
 on genes targeted by more or equal ProbeNbLimit probes and
 on genes targeted by less than ProbeNbLimit probes
 Then for each couple of probesets referenced in Sim files, calculates
 common and uncommon quantities (e.g. the nb of probes in common exons and
 the number of probes in uncommon exons)

INPUT PARAMETERS
 1       PsInfo: probeset information filled by import_targetinfo
 2 ProbeNbLimit: minimum number of tageting probes
   Statistics on different types of paired probesets
 3          Sim: gene(s) targeted inside exons
 4       OutSim: gene(s) targeted outside exons
 5        LHSim: one probeset targeting gene(s) with a low number of probes and the other
                probeset targeting gene(s) with a high number
```

```
 6         LSim: probeset targeting gene(s) with a low number of probes
 7         HSim: probeset targeting gene(s) with a high number of probes
 8  AceviewFlag: if = 1 process Ensembl and AceView genes; if = 0 process only Ensembl genes.
 9   SingleFlag: if = 1 pairs of probesets in Sim target only one genes;  if =0 they target
                 several genes

OUTPUT PARAMETERS
 1 DupInfo is a structure wich keep information about pairs of probesets tested
           and has the following fields for each Sim type (DupInfo{Sim}):
                 psRank1: first probeset rank
                 psRank2: second probeset rank
   Information on genes targeted inside exons
               comGeneIn: list of commonly targeted genes
           meanComGeneIn: geometric mean of common genes relative to the number of genes
                          targeted by each probeset
             uncomGeneIn: list of genes targeted by only one probeset
            comTscriptIn: list of commonly targeted transcripts
           meanTscriptIn: geometric mean of common transcripts relative to the number of
                          transcripts targeted by each probeset
          uncomTscriptIn: list of transcripts targeted by only one probeset
             maxProbe1In: greatest number of targeting probe for the first probeset
             maxProbe2In: greatest number of targeting probe for the second probeset
             minProbe1In: least number of targeting probe for the first probeset
             minProbe2In: least number of targeting probe for the second probeset
       cMeanGroupProbeIn: geometric mean of common probe nb relative to the number of probe
                          targeting common exons in each probeset
       tMeanGroupProbeIn: geometric mean of common probe nb relative to the number of probe
                          targeting all exons in each probeset
   Information on genes targeted outside exons (same fields with Out in place of In)
              comGeneOut .... tMeanGroupProbeOut
                   isGop: if SingleFlag==1, indicates if the single targeted gene is
                          a group of probes (GOP: colocalized probes, but no gene described)

 2 Ps is a structure with the following fields (Ps{Type}{PsRank} with Type=1 for Ensembl
                                                Type=2 for AceView)
   Information on genes targeted by more than or equal to ProbeNbLimit probes:
         geneNamesSup: name of targeted genes
        groupRanksSup: rank of targeted group of transcripts
     groupProbeNbsSup: number of targeting probes in each group
       transcriptsSup: list of targeted transcripts
    notTranscriptsSup: list of not targeted transcripts
           probeNbSup: number of targeting probes in each gene
      geneNamesOutSup: name of genes targeted outside exons
        probeNbOutSup: number of targeting probes in each outside gene
   Information on genes targeted by less than ProbeNbLimit probes:
   geneNamesInf: name of targeted genes
        groupRanksInf: rank of targeted group of transcripts
     groupProbeNbsInf: number of targeting probes in each group
       transcriptsInf: list of targeted transcripts
    notTranscriptsInf: list of not targeted transcripts
           probeNbInf: number of targeting probes in each gene
      geneNamesOutInf: name of genes targeted outside exons
```

### 2.2.7 FILL_PSMATRIX

```
========================
 FUNCTION FILL_PSMATRIX
========================

FILL_PSMATRIX constructs PsMatrix which summarize information on probesets in a
 numeric table

PsMatrix columns:
  1: rank of the assigned gene to the current probeset
  2: position of the assigned gene in NewPs.geneNames
  3: target type of assigned gene
  4: source type of assigned gene
  5: nb of probe targeting the assigned gene
  6: nb of not assigned genes targeted with the same nb of probes
  7: nb of not assigned genes targeted with less nb of probes
  8: nb of groups of transcripts corresponding to the assigned gene
  9: rank of the parent probeset
 10: Rank of the group of transcripts targeted by the current
     probeset in the assigned gene
 11: Rank of the group(s) of transcripts targeted by the current
     probeset, if it is a pivot
 12: [0,1] indicates if the current probeset is a pivot
 13: [0,1] indicates if the current probeset is paired with a pivot
 14: nb of probesets that do not target the assigned gene but target
     a common gene with the current probeset
 15: nb of genes that are targeted by probesets that do not target the
     assigned gene
 16: nb of genes that are targeted by other probeset with a nb
     of probes higher than the number of probes of the current probeset that
     target the assigned gene
 17: ClassRank
 18: and beyond: nb of  other genes targeted with all possible nb of probes
```

### 2.2.8 HYPERGEOMETRIC

```
==========================
 Function HYPERGEOMETRIC
==========================

 HYPERGEOMETRIC calculates probability p(X<=k) if k is less than the expected number
 or p(X<=k) if k is more than the expected number where X is an hypergeometric
 random variable and k the number of elements of the extracted sample
 which have the property

INPUT
 1 InSampleNb: the number of elements of the sample which
               have the property
 2 SampleSize: the number of elements which are randomly extracted
 3   InPopuNb: the number of elements of the population which
               have the property
 4   PopuSize: the total number of elements of the population

OUTPUT
 1 PVal: probability
```

### 2.2.9 IMPORT_TARGETINFO

```
==============================
 FUNCTION IMPORT_TARGETINFO
==============================

IMPORT_TARGETINFO read a series of text files that indicate for each gene the list of
 probesets that target it, with detailed information about the exons, group of exons
 and transcripts that are targeted.

INPUT PARAMETERS
 ChipRank: chip model rank

EXTERNAL FILES
 Read files 'ensembl_probesets_by_gene_%ProbeNb_m%ChipRank.txt'
 (and eventually files 'aceview_probesets_by_gene_%ProbeNb_m%ChipRank.txt')
 with ProbeNb in range (0,n). File with probe nb 0 lists the genes that are targeted out of
 their exons (i.e. in their introns, or in the 2kb upwards and downwards sequence). n is
 the maximum number of probes found targeting a single gene.

 File format: [Gene ID,...
              list of probesets targeting this gene,
              list of corresponding rank,..
              lists of targeted exons (one for each targeting probeset,...
              lists of number of probes in each exon,...
              list of grouped targeted exons (some exons overlap each other),...
              number of probes in each group,...
              list of targeted transcripts,...
              list of not targeted transcripts,...
              list of number of probe outside exons
              list of number of probe inside gene

          ex: ENSMUSG00000000263
              {'100385_at'}
              [9079]
              {{'ENSMUSE00000662385' 'ENSMUSE00000653296' 'ENSMUSE00000307025'}
              {[6 6 2 6 6]}
              {[8 6 7]}
              {[6 6 2]}
              {[1,2,3]}
              {[]}
              {[0]}
              {[15]}

OUTPUT PARAMETERS
 Write sprintf('m%u_probeset_by_ensembl_gene .mat',ModelRank) and eventually
 sprintf('m%u_probeset_by_aceview_gene .mat',ModelRank)

 This file contains:

 EPsInfo for Ensembl genes (APsInfo for Aceview genes)
 a PsNb x n cell with the following structure:

 EPsInfo{PsRank}{ProbeNb}.exonNames
 EPsInfo{PsRank}{ProbeNb}.exonProbeNbs
 EPsInfo{PsRank}{ProbeNb}.groupRanks
 EPsInfo{PsRank}{ProbeNb}.groupProbeNbs
 EPsInfo{PsRank}{ProbeNb}.transcripts
```

```
EPsInfo{PsRank}{ProbeNb}.notTranscripts
EPsInfo{PsRank}{ProbeNb}.inGeneProbeNbs
EPsInfo{PsRank}{ProbeNb}.notInExonProbeNbs

and the following variables for Ensembl genes (the same prefixed with A for Aceview genes):

        EGeneName: cell(n+1,1) containing the list of Gene ID of each input file
                   (genes targeted by n- 1 probes)
       EGeneNames: a list of unique Gene ID contained in EGeneName
    ETargetedGenes: a PsNbxn matrix containing at position i,j the position of GeneName
                   in EGeneName{j}
ETargetingPsRanks: a PsNbxn matrix containing at position i,j the ranks of probesets
                   that target EGeneName{j} with n-1 probes
         EPsNames: cell(n+1,1) containing the list of probeset names of each input file
  EPsRanks=PsRanks: cell(n+1,1) containing the list of probeset ranks of each input file
       EExonNames: cell(n+1,1) containing the list of exon names of each input file
     EExonProbeNbs: cell(n+1,1) containing the list of probeset names of each input file
       EGroupRanks: cell(n+1,1) containing the list of grouped exons
    EGroupProbeNbs: cell(n+1,1) containing the list of number of probes targeting grouped
                   exons
      ETargetedTs: cell(n+1,1) containing the list of targeted transcripts
   ENotTargetedTs: cell(n+1,1) containing the list of not targeted transcripts
```

### 2.2.10 IMPORT_TARGETNB

```
==========================
 FUNCTION IMPORT_TARGETNB
==========================

IMPORT_TARGETNB read a text file (containing either Ensembl or AceView informations)
 and creates a matrix indicating for each probeset the number of genes that have x
 probes targeting their exons (with x>=1 and x<=n(max(probe nb)))

INPUT PARAMETERS
  ModelRank: rank of chip model

EXTERNAL FILES
 Read file 'm%ChipRank_probesets_ensembl.txt'
 and eventually 'm%ChipRank_probesets_aceview.txt'

 File format: [Ps Rank, number of targeted genes with n probes, n-1 probes, ...,1 probe]
              ex : [54, 0, 0, 1, 0, 0, 1, 0, 1, 0, 0, 0, 2, 0, 0, 2, 0]

 Write 'm%ChipRank_probesets_ensembl.mat' containing variable EnsExonGeneNbs
 and eventually 'm%ChipRank_probesets_aceview.mat'  containing variable AceExonGeneNbs
 In output variable, probe nb are in the direct order (1,2,...,n)

FIGURES 16 to 18
```

### 2.2.11 LOAD_DATA

```
===================
 FUNCTION LOAD_DATA
===================
```

```
LOAD_DATA loads data from a binary file

INPUT PARAMETERS
 1      DataFile: data file name
 2       DataDir: data file directory name
 3        LineNb: number of line of the matrix stored in DataFile
 4         ColNb: number of columns of the matrix stored in DataFile
                  data are stored in column order
 5     Precision: type of data
                  Precisions={'int8','int16','int32','int64','uint8','uint16',
                 'uint32','uint64','float32','float64','double','single'};
 6 MachineFormat: endianess
                  MachineFormat={'ieee-be','ieee-le','b','l'}
 varargin:
 7     LineIndex: the index of lines to be loaded
                  Can be alone (=> ColIndex is then set to 1:ColNb)
 8      ColIndex: the index of columns to be loaded
                  In this case, LineIndex must be indicated even if not
                  particular selection is made (=> LineIndex=1:LineNb)

OUTPUT
Success: indicates success/fail of the process {0,1}
   Data: a {LineNb or length(LineIndex)} x {ColNb or length(ColIndex}) matrix
```

### 2.2.12 MAKE_PSGROUPS

```
========================
 FUNCTION MAKE_PSGROUPS
========================


MAKE_PSGROUPS partitionates a series of probesets in groups that can be considered
 as targeting the same transcript(s), based on their properties in
 a particular network or in a set of networks.

 INPUT
 1        PsRanks: ranks of the currently processed probesets
 2      PairedMat: set of vectors indicating the type of relation between each
                   possible couple of probeset in each networks (0: no correlation,
                   1: not significant correlation, 2: significant correlation)
 3          NetNb: nb of networks used
 4      TestLimit: the minimal number of network where a correlation values must exist
                   (either significative and coded by 2, or not significative and coded by 1
                   in PairedMat
 5      GrpSizeNb: number of groups found in previous call of the function
 6       LinkType: indicates if the number of positive networks is counted in PairedMat by
                   counting values <=1 (=1) or <=2 (=2)


OUTPUT
 1    PsGrp: groups of PsRanks
 2 GrpSizes: the distribution of ps group sizes
 3 LinkType: the type of link between to paired probesets (0: no corr, 1: corr>0 but
           not similar (don't pass the test), 2: similar (pass the test))
 4 BadLinks: properties of pairs that don't pass the test but are in a ps group
           [PsL, GeneL, Node1, Node2, Nb of bad links in the group, Total nb of links in
           the group, Nb of corr>0 (PairedMat=1), Nb of significative corr
```

```
          (pairedMat=2)];
5      Hubs: during merging process of triangle, some transitory groups of probesets
          are split into two new  groups.
          single probesets that are common to these two groups are taken away and
          considered as forming a special group called a hub or a pivot which is in
          relation with the two groups.
          [hub rank,first group rank,second group rank,size of the first group,
          size of the snd group]
```

### 2.2.13 MAKE_PSPAIRS

```
=======================
 FUNCTION MAKE_PSPAIRS
=======================

MAKE_PSPAIRS read info on probesets and construct different kinds of pairs of probesets
 targeting eventually the same gene(s) (duplicates)

INPUT PARAMETERS

INPUT

 1      ProbeNbLimit: is the minimal number of probes that a probeset must have in a gene
 2     TargetedGenes: contains matrix (nb of targeted genes x nb  of probes in the targeted
                      gene) in cell (Ensembl & AceView)
                      The matrix line rank corresponds to a gene of same rank in a list of
                      genes (GeneName, not loaded here)
                      The number contained in the matrix, if not null, is the rank of
                      the gene in partial lists of genes (geneNames, not loaded here)
                      example : if 56 is found at position (45,4) of the matrix,
                      it means that the gene GeneName{45} is the 56th in GeneNames{4}
                      If ProbeNbLimit>1, TargetedGenes is not used
 3 TargetingPsRanks: cel with same dimensions than TargetedGenes
                      Indicates the rank(s) of the probeset(s) that target the gene at
                      a given probe nb. If ProbeNbLimit>1, TargetingPsRanks is not used
 4          TestFlag: if =1 indicates that similarity is calculated to find limits on corr,
                                          anti and pv(node neighbourhodd similarity)); if =0 indica
                  similarity is calculated to merge probesets
 5        SingleFlag: used if ProbeNbLimit==1 and indicates if one makes a difference between
                      probesets which target a single gene and those which target several
                      genes (SingleFlag=1 for single genes or 0 for multiple genes)or if one
                      does not make this difference (ProbeNbLimit>1 <=> SingleFlag=[]);
 6            PsInfo: PsInfo{Type}{PsRank}{ProbeNb+1} gives the genes
                      Type=1 => ENSEMBL, Type=2 => ACEVIEW)targeted by ProbeNb probes
                      of the probeset of rank PsRank
                      PsInfo{Type}{PsRank}{1} GIVES THE GENES THAT ARE TARGETED OUTSIDE EXONS
 7         PsProbeNb: the number of probes in a normal probeset
 8           AceFlag: indicates if AceView is used.

OUTPUT
 If ProbeNbLimit>1 no difference is made between single of multiple targets
 If ProbeNbLimit=1 and SingleFlag=1 only probesets targeting a single gene are considered
 If ProbeNbLimit=1 and SingleFlag=0 only probesets targeting several genes are considered

 1            DupStat : distribution of the number of targeted genes
 2            DupRank : probeset ranks belonging to each partition on the number of
                        targeted genes
```

```
    3  GeneRankDuplicate : a list of gene ranks (indexing GeneName list) that are repeated
                           as much as they are targeted by different probesets. If GeneName 45
                           is targeted by 3 probeset, 45,45,45, is present in this list
                           (Ensembl and AceView genes are respectively at the begining
                           and at the end ot the list)
    4    EnsDuplicateOut : list of couple of probesets targeting the same Ensembl gene(s)
                           outside of their exons
    5    AceDuplicateOut : list of couple of probesets targeting the same AceView gene(s)
                           outside of their exons
    6     EnsGeneNameOut : list of the Ensembl gene names targeted outside of their exons
    7     AceGeneNameOut : list of the AceView gene names targeted outside of their exons
    8          Duplicate : list of couple of probesets targeting the same gene(s)
                           in their exons
    9       DuplicateOut : list of couple of probesets targeting the same gene(s)
                           out of their exons
    If ProbeNbLimit==1:
    11      DuplicateLow : 10000 couples of randomly matched probesets present in Duplicates
                           and targeting with less than 3 probes
    10  DuplicateLowHigh : 10000 couples of randomly matched probesets,
                           one present in DuplicateHigh and the other in DuplicateLow
    12     DuplicateHigh : 10000 couples of randomly matched probesets present in Duplicates
                           and targeting with more or = than max(1,ProbeNbLimit-2 probes)
```

### 2.2.14 MERGE_PS

```
===================
 FUNCTION MERGE_PS
===================

 MERGE_PS finds the group of probe sets which target common transcripts on the basis
 of their similar behavior in several networks : high positive correlation, low
 negative correlation and a highly similar neighbourhood, as measured by pv(overlap) which
 is the p-value of observing a given number of common neighbors under a hypergeometric
 distribution



INPUT PARAMETERS
  1        Species: species
  2       ChipRank: chip rank
  3       NetRanks: ranks of networks used
  4   ProbeNbLimit: minimum number of probes targeting a gene
  5     PvCorrRank: pv(overlap) is calculated for corr limit >[0,40,50,60]. PvCorrRank
                    indicates the corr limit to be used, by giving its index in
                    the corr list([0,40,50,60])
  6      StepRanks: list of merge_ps steps to be processed
  7 NetFrequencies: list of net frequencies that mus be considered ([1,25,50,75,100],
                    recommended)
  8    DisplayFlag: indicates if figures must be displayed
  9        SumFlag: indicates if positive networks are those where probeset pair
                    have positive correlation and are similar (=1) or only those where
                    probesets are similar i.e. satisfy all the tested conditions
                    (positive and negative correlation and p-value of overlaping of
                    their neighbourhood) (=0, recommended)
 10        ValFlag: indicates if all values of corr, anti and pv of each pair are used
                    (=1, recommended), or only a single derived value (mean - std) (=0) to
                    calculate limits used for testing probeset pairs
```

```
11       MeanFlag: indicates if limits are calculated from mean of four values (single and
                   multiple targeted genes, and InSim and OutSim) (=1) or only from single
                   targeted genes and InSim (=0, recommended)
12        AceFlag: indicates if AceView data are available
13       IdemFlag: indicates if probeset order is identical in file used by PsawnPy and in
                   networks (=1 in general case, if =0 a m     u_net.txt file must exist
                   in K.dir.rawdata)

MERGE_PS STEPS (StepRanks parameter)
1 CONSTRUCT NEWPS
2 CALCULATE LIMITS
3 COMPLETE NEWPS
4 CONSTRUCT PSBY
5 STAT ON THE THIRD EDGE IN PROBE SET TRIANGLES
6 SUMMARIZE (CONSTRUCT PSMATRIX)
7 FIGURES 51 TO 53
8 WRITE TXT FILES:

  Write PsMatrix files:

    1: first probeset ID
    2: gene ID assigned to the probesets (either Ensembl ID or eventually AceView or GOP id)
    3: gene name
    4: rank of the assigned gene to the current probeset
    5: position of the assigned gene in NewPs.geneNames
    6: target type of assigned gene (1 for probeset in exon, 0 otherwise)
    7: source type of assigned gene (1 for Ensembl, 2 for AceView, 3 for GOP)
    8: nb of probe targeting the assigned gene
    9: nb of not assigned genes targeted with the same nb of probes
   10: nb of not assigned genes targeted with less nb of probes
   11: nb of groups of transcripts corresponding to the assigned gene
   12: rank of the parent probeset
   13: Rank of the group of transcripts targeted by the current
       probeset in the assigned gene
   14: Rank of the group(s) of transcripts targeted by the current
       probeset, if it is a pivot
   15: 0,1 indicates if the current probeset is a pivot
   16: 0,1 indicates if the current probeset is paired with a pivot
   17: nb of probesets that do not target the assigned gene but target
       a common gene with the current probeset
   18: nb of genes that are targeted by probesets that do not target the
       assigned gene
   19: nb of genes that are targeted by other probeset with a nb
       of probes higher than the number of probes of the current probeset that
       target the assigned gene
   20: ClassRank
   21: and beyond: nb of  other genes targeted with all possible nb of probes (from 1 to ProbeNb)

  Write a file describing all the existing probe set pairs
   1: gene ID assigned to the two probesets forming a pair (either Ensembl ID or eventually AceView
    2: gene name
    3: first probeset ID
    4: second probeset ID
    5: first probeset rank in PsMatrix
    6: second probeset rank in PsMatrix
    7: indicates if the probesets are similar in 1% PsMatrix
    8: indicates if the probesets are similar in 25% PsMatrix
    9: indicates if the probesets are similar in 50% PsMatrix
```

```
    10: indicates if the probesets are similar in 75% PsMatrix
    11: indicates if the probesets are similar in 100% PsMatrix
         pivot information for fields 7 to 10, if the two probesets are similar:
         if first and second probesets are not a pivot => 1
         if only one of them is a pivot => 2
         if both are pivots => 3
    12: probeset class (3=MS, 4=MM, 5=CX, 6=HX)
    13: number of probes of the first probeset targeting the assigned gene
    14: number of genes targeted by the first probeset with the same number of probes
    15: number of genes targeted by the first probeset with an inferior number of probes
    16: number of probes of the second probeset targeting the assigned gene
    17: number of genes targeted by the second probeset with the same number of probes
    18: number of genes targeted by the second probeset with an inferior number of probes
    19: class rank of first probe set
    20: class rank of snd probe set
    21: v: the probeset pair is tested in all the networks,
        *: the probeset pair is absent of at leat one networks (~1 of all pairs)
    22: total number of transcripts targeted by the two probesets
    23: number of transcripts targeted in common by the two probesets
    24: number of transcripts specifically targeted by the first probeset
    25: number of transcripts specifically targeted by the second probeset
    26: total number of exons targeted by the two probesets
    27: number of probes located in exons targeted in common by the two probesets
    28: number of probes located in exons specifically targeted by the first probeset
    29: number of probes located in exons specifically targeted by the second probeset
    30: total number of groups of exons targeted by the two probesets
    31: number of probes located in groups of exons targeted in common by the two probesets
    32: number of probes located in groups of exons specifically targeted by the first probeset
    33: number of probes located in groups of exons specifically targeted by the second probeset
    34: overlaping score for transcripts (column 23*100/column 22)
    35: overlaping score for exons
        for each targeted exon a local weighted overlap score, using the number of probes of the
        first (PNb1) and the second (PNb2) probeset targeting this exon, and the total nb
        of probes targeting an exon (PNb)
        (PNb1+PNb2)/PNb)*(min(PNb1,PNb2)/max(PNb1,PNb2)
        The overlaping score is the mean of all local scores
    36: overlaping score for groups of exons
        same method used for the overlaping score for exons
    37: percentage of first probeset probes located in the last exon
    38: percentage of second probeset probes located in the last exon
    39: indicates (1/0) if all probes of the first probeset are located in a single exon
    40: indicates (1/0) if all probes of the second probeset are located in a single exon
    41: percentage of first probeset probes located in the last group
    42: percentage of second probeset probes located in the last group
    43: indicates (1/0) if all probes of the first probeset are located in a single group
    44: indicates (1/0) if all probes of the second probeset are located in a single group
    45 to 67: iAceView information, idem to 22 to 44

  Plots FIGURE 56

SUB FUNCTIONS

   LOADSIM
     Load sim
     INPUT PARAMETERS
      1   ChipRank : the rank of chip set model
      2   NetRanks : a list of network rank
      3 PvCorrRanks: five series of p-values are calculated (on edges with
```

```
                  corr>=0,10,20,30,40,50)
    4   FileName : the common part of or file name to be loaded

   OUTPUT
    AllVal : all CORR,ANTI and PV (p-values) of the networks
       Sim : The last loaded Sim

CONSTRUCT_NEWPS
   construct a new structure (NewPs) containing information about relationships
   between probe sets
   one record per probe set within this structure:
      NewPs{PsL,1}.geneNames: EnsGeneID or Ace gene names targeted by
                                the probeset
        NewPs{PsL,1}.probeNb: nb of probes in each targeted gene
        NewPs{PsL,1}.psRanks: rank ot other probe set which target the
                              same genes
         NewPs{PsL,1}.source: 1=Ensembl genes; 2=AceView genes (not found
                              in Ensembl)
         NewPs{PsL,1}.target: 0= in GOP (group of probe sets that do not overlap a
                              1=in exon or splice; 2=in up, intron or down
   constructs also
        Genes.name: all the gene names targetted by at least one probe set;
      Genes.source: source of the gene (1=Ensembl; 2=AceView)
   and
      PsBy.gene: for each position of Genes, indcates the ranks of the
                 probe sets that target that gene

FILL_PAIRED
   add information in NewPs
      NewPs{PsL,1}.psRanks: other probeset targetting the sames genes
         NewPs{PsL,1}.corr: mean corr between the current probeset and
                            the other probe sets
         NewPs{PsL,1}.anti: mean anti ...
           NewPs{PsL,1}.pv: mean pv ...
        NewPs{PsL,1}.repnb: number of network in which there are
                            significative corr or anti values
      NewPs{PsL,1}.stdcorr: std corr ...
      NewPs{PsL,1}.stdanti: std anti ...
        NewPs{PsL,1}.stdpv: std pv ...
    new pairs of probe sets are recovered at this step which needs
    to calculate their corr, anti, pv, repnb, stdcorr, stdanti & stdpv
    in the networks used

CONSTRUCT_PSBY
    for each targeted gene recover all the probe set that target this
    gene and construct the matrix of their interactions (corr, anti,
    pv, repnb, stdcorr, stdanti, stdpv)

STAT
   OUTPUT

     Stat, a structure containing information about genes targeted by probe sets
        Stat.singleTargNb: The number of genes that are targetted only
                           by the current probe set
        Stat.doubleTargNb: The number of genes that are targetted by the current probe
                           set plus a single other one
      Stat.multipleTargNb: The number of genes that are are targetted by the current
                           probe set plus two or more another probe sets
```

```
    Stat.maxPsNb=zeros: The maximum nb of probe sets targetting a gene targeted
                        by the current probe set
         Stat.grpSizes: the distribution of ps group sizes
        Stat.linkTypes: the type of link (0: no corr, 1:don't pass the test,
                        2 pass the test)
         Stat.badLinks: Properties of pairs that don't pass the test but are in
                        a ps group:
                        PsL,GeneL,Node1,Node2,Nb of bad links,                    Nb
             Stat.hubs: During merging process of triangle, some transitory groups
                        of probe sets are split into two new  groups. Single probe
                        sets that are common to these two groups are taken away and
                        considered as forming a hub which is in relation with
                        the two groups.

  LinkedPs gives information on pairs of probe set that belong to the same group
     LinkedPs{1}: they target a gene with a number of probe smaller than
                  the number of probes of the current ps targeting the assigned gene
     LinkedPs{2}: they target a gene with a number of probe equal to the number
                  of probes of the current ps targeting the assigned gene
                  [Ps1,Ps2,Target type of targeted gene,
                  Nb of probes of Ps1 targeting the gene
                  Nb of probes of Ps2 targeting the gene,
                  Source type of the targeted gene,
                  maximum nb of probes of Ps1 that target a gene,
                  maximum nb of probes of Ps1 that target a gene];
     LinkedPs{1}(PairPos,:): [Ps1,Ps2,NewPs{Ps1}.target(GenePos1),NewPs{Ps1}.
                             probeNb(GenePos1), NewPs{Ps2}.probeNb(GenePos2),
                             NewPs{Ps1}.source(GenePos1),max(NewPs{Ps1}.probeNb),
                             max(NewPs{Ps2}.probeNb)];

TRIANGLE_STAT
   calculate statistics on probe set triangles

SUMMARIZE
    fill PsMatrix:
       1: rank of the assigned gene to the current probe set
       2: position of the assigned gene in NewPs.geneNames
       3: target type of assigned gene
       4: source type of assigned gene
       5: nb of probe targetting the assigned gene
       6: nb of not assigned genes targetted with the same nb of probes
       7: nb of not assigned genes targetted with less nb of probes
       8: nb of groups of transcripts corresponding to the assigned gene
       9: rank of the parent probe set
      10: Rank of the group of transcripts targetted by the current
          probe set in the assigned gene
      11: Rank of the group(s) of transcripts targetted by the current
          probe set, if it is a pivot
      12: [0,1] indicates if the current probe set is a pivot
      13: [0,1] indicates if the current probe set is paired with a pivot
      14: nb of probe sets that do not target the assigned gene but target
          a common gene with the current probe set
      15: nb of genes that are targetted by probe sets that do not target the
          assigned gene
      16: nb of genes that are targetted by other probe set with a nb
          of probes higher than the number of probes of the current probe set that
          target the assigned gene
      17: ClassRank
```

```
      18: and beyond: nb of  other genes targetted with all possible nb of probes

DISPLAY_PS
   display FIG25a,FIG25b

DIST_PROBENB
   calculate the percentage of different source and gene types
```

# PYTHON MODULE INDEX


## b

bintools, 77

## d

dbtools, 78

## p

ps_aceview, 79
ps_class, 71
ps_ensembl, 81
ps_export, 83
ps_import, 84
ps_position, 86
ps_probeset, 87
psawn, 79

## s

setenviron, 89

# INDEX


assign_probeset() (in module ps_probeset), 87

bintools (module), 77

Chip (class in ps_class), 71
chromosome_by_region() (in module ps_ensembl), 81

dbtools (module), 78

ensembl_genes() (in module ps_aceview), 79
exon() (ps_class.Position method), 72
ExonList (class in ps_class), 71
exons_by_gene() (in module ps_ensembl), 81
exons_by_predicted_transcript() (in module ps_ensembl), 81

fill_probesetdb() (in module ps_import), 84

GeneList (class in ps_class), 71
genes_by_region() (in module ps_ensembl), 82
genes_exons() (in module ps_aceview), 80
gop_by_region() (in module ps_position), 86
GopList (class in ps_class), 72

intron() (ps_class.Position method), 72
isInStructure() (in module bintools), 77
isOverlapStructure() (in module bintools), 77

make_chipdb() (in module ps_import), 84
make_probedb() (in module ps_import), 85
make_probeset_list() (in module ps_probeset), 88
make_probesetdb() (in module ps_import), 85
make_speciesdb() (in module ps_import), 85
makebt() (in module dbtools), 78
merge_positions() (in module ps_position), 86

owningStructure() (in module bintools), 78

Position (class in ps_class), 72
position_gopmapping() (in module ps_probeset), 88
position_mapping() (in module ps_probeset), 88
PositionList (class in ps_class), 72
positions_by_region() (in module ps_position), 87
predicted_transcripts_by_region() (in module ps_ensembl), 82
Probe (class in ps_class), 73
Probeset (class in ps_class), 73
probeset() (in module ps_import), 86
ProbesetList (class in ps_class), 74
probesets_by_gene() (in module ps_probeset), 89
process_exons() (in module bintools), 78
ps_aceview (module), 79
ps_class (module), 71
ps_ensembl (module), 81
ps_export (module), 83
ps_import (module), 84
ps_position (module), 86
ps_probeset (module), 87
psawn (module), 79

set_probeset_info() (in module ps_import), 86
setenviron (module), 89
Species (class in ps_class), 75
StructureList (class in ps_class), 75

TargetedGene (class in ps_class), 75
TargetingProbeset (class in ps_class), 76
Targets (class in ps_class), 76
Transcript (class in ps_class), 77
transcript() (in module ps_ensembl), 82
transcript_sequence() (in module ps_aceview), 80
TranscriptList (class in ps_class), 77
transcripts_by_exon() (in module ps_ensembl), 82
transcripts_by_gene() (in module ps_ensembl), 83

up_or_down() (ps_class.Position method), 72

write_aceview_genes() (in module ps_export), 83
write_obj_db() (in module dbtools), 79
write_probeset_list() (in module ps_export), 83
write_probesets_by_gene() (in module ps_export), 84
write_str_db() (in module dbtools), 79
